# Global distribution of Chelonid fibropapilloma-associated herpesvirus among clinically healthy sea turtles

**DOI:** 10.1186/s12862-014-0206-z

**Published:** 2014-10-25

**Authors:** Alonzo Alfaro-Núñez, Mads Frost Bertelsen, Anders Miki Bojesen, Isabel Rasmussen, Lisandra Zepeda-Mendoza, Morten Tange Olsen, Marcus Thomas Pius Gilbert

**Affiliations:** Centre for GeoGenetics, Section for Evolutionary Genomics, Natural History Museum of Denmark, Øster Voldgade 5-7, 1350 Copenhagen K, Denmark; Centre for Zoo and Wild Animal Health, Copenhagen Zoo, Frederiksberg, Denmark; Department of Veterinary Disease Biology, Veterinary Clinical Microbiology, Faculty of Health and Medical Sciences, University of Copenhagen, Copenhagen, Denmark; Trace and Environmental DNA Laboratory, School of Environment and Agriculture, Curtin University, Perth, Western Australia 6845 Australia

**Keywords:** Co-evolution, Sea turtles, Herpesvirus, Fibropapillomatosis, Tumours, Latency, CFPHV

## Abstract

**Background:**

Fibropapillomatosis (FP) is a neoplastic disease characterized by cutaneous tumours that has been documented to infect all sea turtle species. Chelonid fibropapilloma-associated herpesvirus (CFPHV) is believed to be the aetiological agent of FP, based principally on consistent PCR-based detection of herpesvirus DNA sequences from FP tumours. We used a recently described PCR-based assay that targets 3 conserved CFPHV genes, to survey 208 green turtles (Chelonia mydas). This included both FP tumour exhibiting and clinically healthy individuals. An additional 129 globally distributed clinically healthy individual sea turtles; representing four other species were also screened.

**Results:**

CFPHV DNA sequences were obtained from 37/37 (100%) FP exhibiting green turtles, and 45/300 (15%) clinically healthy animals spanning all five species. Although the frequency of infected individuals per turtle population varied considerably, most global populations contained at least one CFPHV positive individual, with the exception of various turtle species from the Arabian Gulf, Northern Indian Ocean and Puerto Rico.

Haplotype analysis of the different gene markers clustered the CFPHV DNA sequences for two of the markers (UL18 and UL22) in turtles from Turks and Caicos separate to all others, regardless of host species or geographic origin.

**Conclusion:**

Presence of CFPHV DNA within globally distributed samples for all five species of sea turtle was confirmed. While 100% of the FP exhibiting green turtles yielded CFPHV sequences, surprisingly, so did 15% of the clinically healthy turtles. We hypothesize that turtle populations with zero (0%) CFPHV frequency may be attributed to possible environmental differences, diet and/or genetic resistance in these individuals. Our results provide first data on the prevalence of CFPHV among seemingly healthy turtles; a factor that may not be directly correlated to the disease incidence, but may suggest of a long-term co-evolutionary latent infection interaction between CFPHV and its turtle-host across species. Finally, computational analysis of amino acid variants within the Turks and Caicos samples suggest potential functional importance in a substitution for marker UL18 that encodes the major capsid protein gene, which potentially could explain differences in pathogenicity. Nevertheless, such a theory remains to be validated by further research.

**Electronic supplementary material:**

The online version of this article (doi:10.1186/s12862-014-0206-z) contains supplementary material, which is available to authorized users.

## Background

Studies on the evolution of pathogen virulence and host resistance have shown that within populations, both pathogen and host are able to adapt in response to the interactions [1,2]. However, there is much debate on how these micro-evolutionary scale changes can influence the patterns of speciation of the interacting species at macro-evolutionary levels [2]. Co-evolution does not necessarily lead to the co-speciation of the interacting species [3]. However, co-adaptation theory suggests a general trend of parasite specialization for their hosts [4], regardless of the age of the association. Retroviruses and herpesviruses, with their vertebrate hosts, are good examples of specialist pathogens for which co-evolution leading to a host-specific occurrence over long periods of time and are exquisitely well adapted to their host [5-7]. Thus, herpesviruses are well adapted to their hosts, most likely as a consequence of prolonged co-evolution [6-8]. Herpesviruses are generally characterized by their variable host range, short replication cycle, and the ability to destroy infected cells and establish latent infection [9]. Within these features of herpesviruses pathogenesis, we particularly emphasize on latency, which is defined as persistent life-long infection of a host with restricted, but recurrent, virus replication [10].

Fibropapillomatosis (FP) is a debilitating neoplastic disease that globally affects sea turtles, and is characterized by the presence of epithelial fibropapillomas and internal fibromas [11,12]. Originally described in green turtles (Chelonia mydas), FP has subsequently been documented among all seven species of sea turtles [8,11,13-18] in all major oceans, and thus has a circumtropical distribution [19]. Prevalence estimates based upon FP records varies among locations, ranging from as low as 1-2% to as high as 90% [11], thus FP has been described as an emerging disease with sporadic, but generally increasing, occurrence [12]. While environmental aspects are suspected to be cofactors for the outbreaks [20,21], the Chelonid fibropapilloma-associated herpesvirus (CFPHV) has been proposed as the etiologic agent responsible [14,20]. As with most other herpesvirus infections, CFPHV infections are believed to result in a long, balanced interaction with the turtle host, thus allowing efficient virus transmission for years: lifelong latent infection is established only interrupted by episodes of viral reactivation and potential replication [22,23]. As such, sea turtles may be infected, thus carrying entire segments of viral DNA and/or even the entire viral particle, even when no FP tumours can be observed.

Despite the consistent PCR-based detection of herpesvirus DNA sequences in FP tumour samples [14,15,24], attempts to cultivate CFPHV in vitro have been unsuccessful [24]. Consequently the role of CFPHV as the causative agent for FP tumours remains inconclusive.

Given CFPHV’s putative causal role in FP, to date most studies into the epidemiology, pathology, DNA detection, prevalence and phylogeography of CFPHV have been performed using DNA extracted from tumour tissue [8,19,24,25], thus cannot be used to estimate the prevalence of latent CFPHV infections. While a small number of studies have attempted to detect and quantify viral DNA from tumour free tissue [12,26-28], the samples used were either from known FP infected animals, or from localized populations known to be infected by the virus. Thus, the global viral prevalence in turtles that do not exhibit tumours remains unknown. Furthermore, to date only a limited number of studies have attempted to investigate amino acid substitutions within CFPHV sequences, whether for determining phylogenetic relationships of different CFPHV strains or long-term virus/host co-speciation [8,29].

In this study, we used a recently validated PCR assay that targets three short regions of conserved genes [18] within the herpesvirus, to provide insights and evidence into the prevalence, co-evolution, phylogeography and genetic variation of CFPHV in tumour and non-tumour containing samples of sea turtles. The dataset studied included FP tumours from green turtles sampled from three different distant populations; Hawaii in the Pacific, Turks and Caicos [30] in the Caribbean, and Principe Island [31] off the Western Coast of Africa, as well as tissue samples from clinically healthy (not FP exhibiting) green, loggerhead (Caretta caretta), olive Ridley (Lepidochelys olivacea), hawksbill (Eretmochelys imbricata) and leatherback (Dermochelys coriacea) sea turtles from globally-distributed populations.

## Results

### Viral detection and prevalence

A total of 398 DNA extracts from 337 individual sea turtles were screened for herpesvirus by PCR. In turtles that presented more than one area of suspected FP tumour, samples were collected from each suspected site. CFPHV sequences were obtained from 132 samples derived from 82 individual turtles, representing 24% of the total sea turtles analysed. 100% of the 66 FP tumour biopsy extracts (representing 37 individual green turtles) were positive for CFPHV. Viral sequences were also detected in 88% of the non-tumour DNA extractions from the same FP infected green turtles. The average detection level among the clinically healthy sea turtles (including green turtles) was 15% (Table 1).

**Table 1:** **Percentage of CFPHV detection by turtle species**

**Turtle species common name**		**Number of turtle individuals analysed**	**Results from PCR CFPHV detection by type of tissue**			**Total turtles detected CFPHV positive**	**% of CFPHV detection by turtle species**
			**FP exhibiting**	**healthy + CFPHV**	**CFPHV free**		
**Green (** ***Cm*** **)**		208	37	24	147	61	29.3%
**Loggerhead (** ***Cc*** **)**		61	0	7	54	7	11.5%
**Leatherback (** ***Dc*** **)**		20	0	4	16	4	20.0%
**Hawksbill (** ***Ei*** **)**		28	0	7	21	7	25.0%
**Olive Ridley (** ***Lo*** **)**		20	0	3	17	3	15.0%
**Total sea turtle individuals analysed**		337	37	45	255	82	24.3%

Summary table of number of turtle species individuals analysed for Chelonid fibropapilloma-associated herpesvirus (CFPHV) DNA. Results are listed as either CFPHV positive in FP exhibiting turtles clinically healthy carrying CFPHV DNA, or CFPHV free (CFPHV negative) in clinically healthy turtles. For detailed description of population species see Additional file 2.

Of the 208 total green turtles analysed (FP exhibiting and clinically healthy), 61 (29%) were PCR positive for CFPHV. For the other species, all of which were represented by clinically healthy animals only, 20% of the 20 leatherback turtle samples, 12% of the 61 loggerhead turtles, 15% of the 20 olive Ridley turtles and 25% out of 28 hawksbill turtle samples were CFPHV positive. Summarized PCR results, population origin of turtle species DNA extracts analysed, minimum CFPHV prevalence estimated and detailed average detection per populations species are listed in Additional files 1 and 2.

### Sequencing, evolutionary models and haplotype analysis

All 132 viral positive DNA extractions were sequenced for one or more of the three loci. Detailed list of haplotype sequence SNPs per marker can be found in Additional file 3. CFPHV DNA and amino acid sequences and haplotype SNPs detected were deposited in Dryad repository, with reference number DOI:10.5061/dryad.8r082 [32]. The final 98 nucleotide alignment of the 58 sequences for UL18 locus exhibited five different variable sites (position 6 R, position 12 Y, position 40 Y, position 69 R and position 78 R). The 132 nucleotide alignment of the 95 sequences for UL22 locus contained two variables sites (position 42 R and position 90 W). Finally, the 144 nucleotide alignment of the 75 sequences for UL27 locus yielded two variables sites (position 23 R and position 127 Y).

Using the Bayesian Information Criterion (BIC), the Kimura (K80) substitution model was selected for the alignment in UL18, whereas Jukes & Cantor (JC69) was determined for the alignments in UL22 and UL27 (Additional file 4).

Topologies for UL18 and UL22 phylogenetic trees followed a similar pattern clustering most viral sequences originated from Turks and Caicos into a separate branch, while the rest of sequences across species and locations were clustered into a main branch. Moreover, one unique green turtle viral sequence from Portugal, respectively for UL18 and UL22, was also clustered into this alternative branch. The phylogeny for UL27 did not provide geographical or turtle species structure spreading the sequences into three distinct branches showing no pattern (Additional file 5).

**Figure 1:**
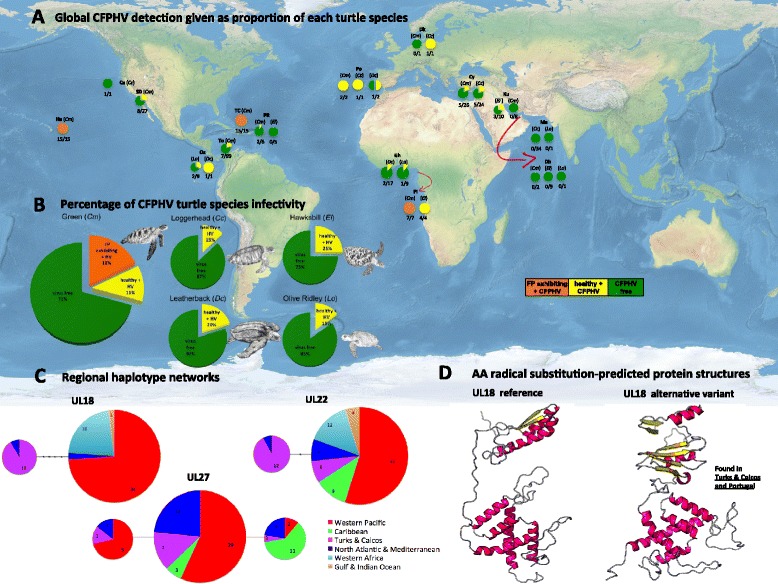
**CFPHV global detection, haplotype networks and AA protein structures. (A)** Global CFPHV detection given as proportion of each turtle species’ populations. Different colours represent category of tissue sample and infectivity status as follows; Orange = FP exhibiting turtles found CFPHV positive, Yellow = clinically healthy turtles also CFPHV positive, and Green = clinically healthy turtles CFPHV negative (CFPHV free), **(B)** Percentage of CFPHV turtle infectivity grouped by different turtle species (same colour code as panel **A**), **(C)** Regional haplotype networks for markers UL18, UL22 and UL27 clustered by regional sample origins, and showing the respective number of haplotypes found per marker, and **(D)** Amino acid radical substitution-predicted protein structure models for marker UL18, where globally distributed samples have the reference structure, while green turtles from Turks and Caicos, plus one green turtle from Portugal correspond to the alternative variant structure.

Haplotype networks for UL18 and UL22 loci were in agreement with the phylogenetic trees, showing a separate regional unique haplotype for samples originated from Turks and Caicos in the Caribbean and one unique sample from Portugal in the North Atlantic (See Figure 1C). However, no geographic structure was discernable for the three different haplotypes detected for marker UL27.

### Predicted effect of amino acid changes

The UL18-Viral Capsid exhibited a Y/C amino acid variant at position 100 of the protein reference sequence (protein_id: AHA93343.1). All 11 of the Turks and Caicos green sea turtle samples, and one of the two Portuguese samples exhibited the Y form representing a 20.7% of the total UL18 viral sequences, while all other samples studied exhibited the C (79.3%). The structural effect of the substitution in comparison to the reference sequence was analysed using in silico 3D modelling. The amino acid substitution may fall in a determined protein domain with or without a reference template from Protein Data Bank (PDB) [33] for the 3D modelling and the resulting model comes from a different number of predictions.

According to the structures modelled for the entire protein sequence, the reference amino acid was positioned in a beta SS3 domain in 73.5% of the RaptorX predictions, in 11% of the predictions it fell in a helix and in 15.5% in a loop. This domain was modelled without the use of a template from PDB due to lack of them. The solvent accessibility for the reference amino acid was labelled as buried (53.6%), medium (36.6%), and exposed (10.1%). The alternative amino acid also was placed in a beta structure with 81.3% (4.3% as helix and 14.2% loop) according to the labelling of the whole sequence almost entirely modelled using a template from PDB (93% of the residues were modelled using a template, the alternative Y amino acid included). In the 3D domain predicted from PDB templates with a p value of 1.31^e-02^ 100Y fell at the end of a loop, right before the beginning of a beta. The solvent accessibility for Y was labelled as buried (48%), medium (38.2%), and exposed (13.9%). The predicted structures of the reference and the sequence associated with the radical amino acidic substitution were different, with the first predicting two domains and the later predicting three domains (See Figure 1D).

Marker UL22-Glycoprotein H also contains variation of the protein reference sequence (protein_id: AHA93347.1), with a variant codon (E/D) at position 126; 126 E mainly corresponds to samples from Turks and Caicos, 126 D to the rest of the world. 78% of the residues could be modelled with a PDB template. The reference amino acid form this conservative amino acidic change fell in a loop with 64.3%, with no PDB template. 126 D was positioned very close to a modelled with PDB template in a region with a long loop, further supporting its loop classification.

Finally as UL27-Glycoprotein B, a codon variant was identified as T/M at position 837 also of the protein reference sequence (protein_id: AAU93326.1). This amino acid variant exhibits no obvious phylogeographic distribution by species or population. 93% of the residues could be modelled with a PBD template, not including 837 T, but it fell very close to a modelled long loop region with PDB template, further supporting its loop classification.

## Discussion

### Prevalence, co-evolution and latency

Although previous analyses have assayed the prevalence of the FP disease among turtles, our results provide first data of the prevalence of CFPHV among apparently healthy turtles, something that may not be directly correlated to the disease incidence, but directly provides evidence suggesting a long-term co-evolutionary latent infection interaction between CFPHV and its turtle-host across species. This is not surprising, as the host-specific occurrence of herpesviruses has been well documented, suggesting they have evolved with their hosts over long periods of time [5,6]. A specific host response to infectious agents is suspected to be highly heritable, but recognizing the genetic variants that may responsible for the disease susceptibility and pathogenicity has proven challenging [34]. A main goal in infectious disease research is to identify the host-pathogen genetic variants that may explain differences in pathogenesis. Disrupted co-evolution between host-pathogen interactions have recently been proposed to explain the variation in disease outcomes, which until now have not been able to be proven adequate to explain the heterogeneity of disease pathology [34]. This clearly incorporates the limiting factor that both host and pathogen genetic profile must be taken into account when studying disease aetiology in order to draw conclusions. Co-evolutionary theories support the hypothesis that the processes of co-adaptations would lead to a general trend of parasite specialization for their hosts [4], regardless of the age of the association. However, it is important to bare in mind that co-evolution does not necessarily lead to the co-speciation of the interacting species [3]. This long term co-divergence between CFPHV with its turtle hosts has been addressed and analysed previously, estimating that virus and host have co-evolved for at least 8.9 million of years (Ma) [8]. A subsequent study that reported the sequence of a large proportion of the CFPHV genome, for use in investigating its phylogenetic structure across the global distribution of marine turtle species, similarly concluded that co-evolution happened over a scale of millions of years [35]. We acknowledge that the dataset presented in this study is small in terms of length of sequence analysed uniquely for the herpesvirus. Additionally, DNA sequences of the same length were downloaded from the database at GenBank in order to analyse the phylogeny more in detail. For UL18 and UL22 markers only two other sequences of the same length position were found. Nonetheless the sequences could not be used as for origin location of the samples was missing. For UL27 marker, 21 other viral sequences were found and tested. K80 was selected as substitution model for phylogeographic analysis of the new dataset. Nevertheless the phylogenetic results of alignment did not provide species or geographic structure distributing the sequences across five branches without a distinctive pattern (see Additional file 6). Consequently, we caution the general interpretation of the phylogenetic results presented here, which may not reflect the real genetic structure and distribution of the CFPHV. However, our data includes a large number of samples that span a considerable number of different geographic locations, across five turtle species. Thus our data points to the fact that the emergence of fibropapillomatosis epizootics at multiple locations around the world during the past decades is unlikely to be due to recent virulence mutations in the virus, because it is highly improbable that such mutations could occur independently in lineages [13].

Another aspect playing an important role in this research is latency. Herpesviruses have the ability to establish latent infections in their host, during which viral gene expression is minimized [5]. Moreover, some oncogenic herpesviruses induce tumour formation in specific tissues [20], while being absent in other tissues of the same individual. Latency is generally maintained by viral genes and sites expressed primarily during latency [36]. Expression of these latency-associated genes may function to keep the viral genome from being digested by cellular ribozymes or being found out by the immune system. Thus, CFPHV DNA may be unequally distributed within same animal with large concentrations of viral DNA in the affected tissue only. Given this, it is not surprising that CFPHV viral DNA sequences were detected in 100% of FP tumours. Although only 88% of the samples derived from non-tumour areas of FP exhibiting turtles were CFPHV positive, this is also unsurprising - while in the active viraemic phase, herpesvirus DNA is prone to be detected throughout the body, it is likely that in chronic infections viral activity may be confined to nervous tissue [5,37] and tumour sites.

Lastly, in the globally distributed samples of clinically healthy individuals belonging to 5 species of sea turtles, the average detection rate was substantially lower; 15%. While we observed a great variation in the ratio of infected individuals in the populations investigated, nearly all sea turtle populations analysed counted some individuals carrying CFPHV (see Figure 1A), which seems to support the panzootic status of FP [11,38]. Excluding location samples from which only a single individual was analysed - e.g. loggerhead sample from Northern California or green turtle from Danish aquarium -, unique exceptions seem to be grouped in the small sample sizes originated from Kuwait, Dubai and Oman in the Arabian Gulf and Northern Indian Ocean, as well as hawksbill turtles from Puerto Rico which remained CFPHV free in our global PCR analysis (see Figure 1A). This could indicate that the specific turtle individuals analysed in these areas could be resistant to CFPHV and/or suggest that certain environmental conditions possibly in combination with immune system modulators may influence the persistence and severity of the disease [11,39-42]. There are no historic records for turtles exhibiting FB tumours for the entire Arabian Gulf region, nor for the hawksbill population in Puerto Rico. However, CFPHV presence was detected in three-hawksbill turtles from Kuwait. Thus, apparently CFPHV-free turtle populations should be observed individually and not as a regional phenomenon. Moreover, there is no genetic data available for green turtles nesting at Qaru Island in Kuwait, however, satellite telemetry data suggests this population derive from a larger stock nesting in Saudi Arabia [43]. In order to analyse the large geographical distribution of Masirah Island’s loggerhead population, a total of 34 turtle individuals were collected over 80 kilometres coastline from the Omani population. This population stock in combination with the rest of the Indian Ocean has been estimated to be second largest loggerhead population in the world [44,45], as well as possessing one of the unique haplotypes in their clade, which diverges from the remaining Atlantic and Indian Ocean haplogroup IB lineages [46]. It is important however, to acknowledge the small sample sizes used in this study for both FP infected and non infected turtle individuals, as this may not fully represent the actual CFPHV infectivity prevalence.

Herpesviruses are frequently detected in tortoises and terrapins of zoological collections mainly because of the advances in diagnostic methods, and several species, mainly tortoise herpesvirus 1 to 4 (THV1-4), have been reported and characterized [39,47-49]. As our PCR primers used were all based on published sequences of conserved genes [6,50] in the CFPHV genome, we speculated they may potentially anneal successfully to related herpesviruses. However, a preliminary result from a small data set of 11 DNA extracts derived from 9 different species of tortoises and terrapins (Additional files 3 and 7) yielded no positive results. Whether this indicates that CFPHV is unique to sea turtles, or that tortoise and terrapin herpesvirus is too divergent for the primers to bind to remains uncertain, and warrants future investigation.

Herpesviruses are well known to be transmitted horizontally from one infected organism to the other by direct physical contact via saliva, mucus, blood or semen [6]. Viral particles can also be transported and survive in salt water for relatively short periods of time before the proteins in the virus capsid degrade. This has been reported for the herpesvirus associated with lung-eye-trachea disease (LETD) in sea turtles, which has been shown to survive in seawater for about 2 weeks [51]. Sea turtles have been observed to develop FP tumours as juveniles and it has been suggested that infection occurs in feeding grounds where animals conglomerate in large numbers [13,52]. It is possible to hypothesize, therefore, that the transmission of the virus occurs mainly during this period in the life cycle of sea turtles. Thus, animals may either develop infection or simply remain passively infected in latency while viral DNA establishes into the animal tissue for the rest of their life. Moreover, we speculate that severity of infection may depend on co-infections and the immunological status of the individual animal, which also may explain why not all animals in a population develop FP [38].

We hypothesize that there is little or no chance for vertical disease transmission from mother to offspring as it has been suggested before for sea turtles. However, there is a possibility of heritable-genetic disease susceptibility prior exposure to whatever the infectious agent is, as previously proposed by Herbst et al. in 1995 [20]. The only population that we obtained neonate samples from was Tortuguero green turtles in the Caribbean of Costa Rica. Samples from this stock were analysed for nesting females (n = 41) and offspring (n = 59) from 6 different clutches. One nesting female, which was the mother of one clutch analysed, was CFPHV positive (UL22), 10 of its offspring however, were PCR negative. CFPHV sequences were detected only from six females in this population; all offspring neonates resulted in zero prevalence. While our data is not conclusive, future studies should examine the genetic signature mother-offspring in viral disease transmission and tumour development.

### Amino acid variation in the Turks and Caicos population

From the different amino acid substitutions found in the haplotypes, only the C > Y radical substitution at position 100 in the UL18 marker gene is of potential functional importance. All other substitutions observed were either conservative, or part of a loop. Although no high-resolution crystallographic structure exists for the protein encoded by this gene, thus limiting the potential for functional prediction, it was possible to use RaptorX to predict structural changes with other suitable templates. Modelling of the 100 YC substitution indicates a significant difference between the reference and the alternative sequence, with the alternative sequence having an extra domain compared to the reference (See Figure 1D). This could be due to the availability of more PDB templates for the alternative sequence, or reflect an actual change in the protein folding due to this particular residue which has high probabilities of forming part of a beta ladder or at least be right before the beginning of one, suggesting its importance to the protein structure. This is of increased interest when taking into account the characteristics of these two amino acids, as the cysteine has a thiol side chain group that often engages in enzymatic reactions, and its oxidized form, cysteine, has an important structural role in many proteins. On the other hand, the tyrosine has a phenol side chain group, which allows it to receive a phosphate from kinases in signal transduction cascades, changing the activity of the protein. Given that the function of the major capsid protein is to protect the viral genome from damaging agents such as proteolytic and nucleolytic enzymes, such an amino acidic change could have functional as well as structural implications.

FP tumours have previously been reported for green turtles in Turks and Caicos Islands (TCI’s) [30,53], but no genetic characterization has been done of the infected animals at this location. Mixed-stock analysis of conglomerating green turtles from TCI’s, have shown the mitochondrial haplotype CM-A3 as the most common, a widespread haplotype in Caribbean rookeries, including Costa Rica (Tortuguero), Florida and Mexico [30]. This indicates that TCI’s green turtle aggregations may receive important contributions from all over the Caribbean and possibly also from the rest of the Atlantic. However, there is no evidence suggesting a genetic flow between these and populations from far Northeast or from the other side of the Atlantic e.g. in Portuguese waters. Both haplotype and amino acids characterization performed in this study indicate a unique genetic signature for the CFPHV in TCI’s, shared only by one sample originated in Portugal, which may suggest this virus strain has spread across the Atlantic. It is important to notice that all turtles from TCI’s containing this virus strain were FP exhibiting, while the unique turtle from Portugal was a clinically healthy animal with no signs of disease. Furthermore, the amino acid substitution described for UL18 gene may as well suggest an important protein structure modification that could potentially result in the viral capsid being more resistant to the host immune system. As has been shown before, even a single amino acid substitution can be the cause of viral drug resistance and enhanced virulence [54-56]. Even though its positioning in the protein structure is based only on computational modelling and the validity of its importance is subject to different conditions, such as the amino acid being at least moderately exposed instead of buried, it is worth noting it as a target for future deeper analysis with laboratory experimental techniques.

## Conclusions

Our data reveals the presence of CFPHV DNA within globally distributed samples for all five species of sea turtle (See Figure 1A-B). While the 100% incidence of CFPHV in samples taken from tumour exhibiting green turtles is in concordance with the findings of previous studies [27,28,51,52], the key new observation is a non-negligible prevalence among clinically healthy animals of all 5 species. Surprisingly this yielded an average of 15% of CFPHV infected individuals, and evidences the latent infectivity co-evolutionary process across virus and host.

While the frequency of infected turtle individuals from the different sites varied considerably, as well as the sample sizes studied, most global sample sets contained at least one CFPHV positive individual. One notable exception is that of the various turtle species from the Arabian Gulf and Northern Indian Ocean, as well as hawksbill turtles (Eretmochelys imbricata) from Puerto Rico, which were CFPHV free. We hypothesize that this may suggest a possible environmental difference and/or genetic resistance in these individuals.

Haplotype analysis results were in agreement for markers UL18, UL22 (See Figure 1C); clustering CFPHV DNA sequences in turtles from Turks and Caicos separate to all others, regardless of host species or geographic origin. Larger nucleotide amps to full genomic data are advised to be used to address phylogeography and genetic relationships within the CFPHV clade.

Finally, computational analysis of amino acid radical substitution variants within the Turks and Caicos samples suggest potential functional importance in a substitution for marker UL18 (See Figure 1D) that encodes the major capsid protein gene. This could potentially explain differences in pathogenicity as well as the presence of different CFPHV genotypes at different locations. Further research is however advised in order to validate our suggested theory.

## Methods

### Samples

Tissue sampling design and protocols varied among the different collaborators who provided samples from across the world. Overall, 398 tissue samples from 337 individual turtles of five species of sea turtles were obtained, taken from skin, FP tumours or blood. Sample material obtained were categorized into three different groups for analysis; 1) tumours from turtles exhibiting FP, 2) any other type of tissue with macroscopic absence of tumour (non-tumour) taken from FP exhibiting turtles; and 3) any type of tissue or blood from clinically healthy (not FP exhibiting) turtles. FP tumour samples derived from three populations of green turtles: Hawaii in the Pacific, Turks and Caicos in the Caribbean, and Principe Island off the Atlantic coast of Africa. For other tissue samples from clinically healthy turtles (thus not FP exhibiting) see details in Additional file 7. All samples were collected and exported under CITES permits (host institute permit DK03).

Skin samples were preserved in either RNA later® (Qiagen), DMSO 20% saturated with NaCl or in 70-96-100% ethanol at -20°C; blood samples were preserved in PAXgene™ (Qiagen).

### DNA extraction of skin and tumour samples

DNA was extracted from approximately 25 mg tissue using the DNeasy Kit (Qiagen, Valencia, CA) following the instructions of the manufacturer. Samples were incubated with agitation for 24 h at 56°C. The samples were then centrifuged for 5 min at 10,000 g to pellet any remaining cellular material and the supernatant was transferred into a 2 ml DNeasy Mini spin column placed in a 2 ml collection tube and centrifuged for 1 min at 8,000 g. Finally purified genomic DNA was eluted in 100 μl of Qiagen EB buffer. Extraction blanks were included to monitor for contamination.

### DNA extraction from blood samples

DNA was extracted from approximately 10-20 μl anticoagulated blood by proteinase K digestion using the DNeasy Blood & Tissue Kit (Qiagen, Valencia, CA) following the manufacturer’s instructions.

Samples were incubated with agitation for 10 min at 56°C. The samples were then centrifuged for 1 min at 10,000 g, ethanol 96% was added to each sample and the supernatant was transferred into a 2 ml the DNeasy Mini spin column placed in a 2 ml collection tube, and then centrifuged for 1 min at 8,000 g and washed two times with Qiagen AW1 and AW2 buffers and finally purified genomic DNA was eluted in 200 μl of Qiagen AE buffer. Extraction blanks were included to monitor for contamination.

### PCR assay

PCR protocol specifications from a recently developed PCR-assay [18] were followed. The assay uses singleplex primers designed to target highly conserved regions for three different genes [6,50] in the herpesvirus genome (Glycoprotein B gene UL27, Glycoprotein H gene UL22 and Mayor capsid protein gene UL18-UL19) (primer sequences are listed in Additional file 8). To minimise the risk of over estimating the prevalence, a conservative approach was taken with regards to positive result acceptance. Firstly, all PCR results were confirmed through repeating the PCR at least twice. Secondly, every positive viral amplicon identified was confirmed by Sanger sequencing; any amplification that lacked sequence verification was considered a false positive and excluded from the analysis.

PCR amplicons of the expected size were purified using QIAquick columns (Qiagen) following the manufacture’s protocol, then sequenced in both directions by the commercial service at Macrogen Inc., Europe. Negative controls were included in each batch of PCR amplifications and next sequencing reactions to detect contamination.

### Data analysis

A total of 287 purified PCR amplicons were sequenced using BigDye v3.1 (Applied Biosystems, USA) and 3730XL DNA Analyzer (Applied Biosystems, USA) at Macrogen Inc., Europe.

DNA sequences were edited and aligned using Geneious Pro 6.1.7 (Biomatters Ltd.) to give a consensus sequence for each amplicon. Thereafter, sequences were blasted against NCBI GenBank and assigned to herpesvirus based on minimum 95% pairwise identity. For any BLAST result yielding a lower identity, the sequence was manually re-checked, and considered false positive if the sequence was not obviously derived from the herpesvirus target and thus excluded from further analysis. Sequences were truncated to alignments for each gene marker. mafft v6.814b as implemented within geneious was used to align the sequences for each amplicon marker, using a scoring matrix of 200PAM/k2, gap open penalty: 1.53 and offset value: 0.123).

### Phylogenetic analyses

Substitution model selection for each of the three aligned fragment sets was inferred in jmodeltest v2.1.6 [57,58]. The best-fitting model our of the 88 evolutionary models were selected according to the BIC (Additional file 4) were used for the phylogenetic analyses since this method is known to perform better than other criteria [59]. Maximum likelihood trees were generated using phyml v3.0 [60] with the most suitable model for each target region (1000 non-parametric bootstrapped data sets) to illustrate the genetic relationship between the different sequence samples and plotted in figtree v1.4.2 [61] (Additional file 5).

### Haplotype analysis

Number of variable sites was determined and analysed for each alignment for each gene short-fragment marker (UL18, UL22 and UL27). Spatial distribution of virus strains was visualized by creating haplotype networks using TempNet [62], an R-script for summarizing heterochronous genetic data implemented in r version 3.0.2 [63] with rstudio version 0.98.490.

### Amino acid analysis

In order to identify any amino acid substitution that may have functional implications between different virus haplotypes, the sequences from each marker were aligned to the corresponding gene sequence downloaded from NCBI GenBank, then translated in the corresponding frame using the standard genetic code. Subsequently, the sequence structures of the marker proteins with and without the amino acidic substitution were predicted with RaptorX [64] and the protein secondary structure in which the changed amino acid fell was identified from the reported structure labelling for the whole sequence as well as from the 3D model for the corresponding domain when possible. RaptorX refers to a domain in the sense of a model unit. Furthermore, the predicted structure model of the whole sequence with the reference and alternative amino acid were compared, as well as the predicted solvent accessibility of the reference and alternative amino acid that reports a probability of the amino acid being buried, exposed, or moderately exposed.

### Availability of supporting data

CFPHV DNA and amino acid sequences and haplotype SNPs detected were deposited in Dryad in the Availability of Supporting Data section of this article, with reference number DOI:10.5061/dryad.8r082 [32]. The haplotype and amino acid sequences list data set supporting the results of this article are also included in the Additional file 3.

## Additional files

Additional file 1:
**Summarized list of DNA extractions analysed grouped by population and turtle species; and Description of data.** Summarized results list of DNA extractions analysed grouped by population and turtle species presented by each PCR assay per locus marker, total viral detection and proportion of CFPHV infected turtles calculated by the total number of DNA extracts. Additional file 2:
**Detailed number of turtle species individuals analysed for the CFPHV DNA detection (see Additional file** 1 **); and Description of data.** Listed results per turtle species as either CFPHV positive in FP exhibiting turtles (orange colour) and clinically healthy carrying CFPHV DNA (yellow colour), or CFPHV free (CFPHV negative) uniquely for clinically healthy turtles (green colour). Additional file 3:
**Haplotype and amino acid sequences list; and Description of data.** Detailed list of haplotype DNA sequences and amino acid variants found for the highly conserved regions for three short-fragments markers (UL18, UL22 and UL27) within the CFPHV genome. Additional file 4:
**Table list with the best inferred substitution models; and Description of data.** Detailed table list with the best inferred substitution models using JModelTest software for the alignments of the sequence data generated in this study for UL18, UL22, UL27 markers and for the alignment of the same dataset for UL27 plus all the available sequences at GenBank database. Additional file 5:
**Circular maximum likelihood phylogenetic trees; and Description of data.** Circular maximum likelihood phylogenetic trees for (A) UL18, (B) UL22 and (C) UL27 sequence alignments generated in this study. Additional file 6:
**Circular maximum likelihood phylogenetic tree for UL27 + GenBank; and Description of data.** Circular maximum likelihood phylogenetic tree of combined alignment generated from UL27 sequences produced in this study and available sequences found at GenBank database. Additional file 7:
**Detailed sample material analysed for CFPHV detection; and Description of data.** List of DNA extracts analysed for DNA viral detection of the Chelonid fibropapilloma-associated herpesvirus (CFPHV) including, sample ID, species, type of tissue, and population origin. Moreover, PCR assay results by individual marker and confirmation of CFPHV DNA by Sanger sequencing. Additional file 8:
**Primer sequences; and Description of data.** Singleplex primer sequences designed to target highly conserved regions for three different genes in the herpesvirus genome [18].
