# Supplementary material for: Global distribution of Chelonid fibropapilloma-associated herpesvirus among clinically healthy sea turtles
Source: BMC Evol Biol. 2014 Oct 25;14:206. doi: 10.1186/s12862-014-0206-z (PMC4219010; doi:10.1186/s12862-014-0206-z)
Supplement: Additional file 1: — Summarized list of DNA extractions analysed grouped by population and turtle species; and Description of data. Summarized results list of DNA extractions analysed grouped by population and turtle species presented by each PCR assay per locus marker, total viral detection and proportion of CFPHV infected turtles calculated by the total number of DNA extracts. [file 12862_2014_206_MOESM1_ESM.pdf]

Sea turtle population or location of sample source	Species	Number of analysed DNA extracts	Number of turtles analysed	Results from PCR CFPHV detection per locus, confirmed by sequencing			Total CFPHV detection in DNA extracts	% of CFPHV per DNA extracts
				UL18 (n)	UL22 (n)	UL27 (n)		
Denmark, Danmarks Aquarium	*Caretta caretta*	**1**	**1**	1	0	1	**1**	**100,0%**
Denmark, Danmarks Aquarium	*Chelonia mydas*	**5**	**1**	0	0	0	**0**	**0,0%**
Dubai, EAU	*Chelonia mydas*	**2**	**2**	0	0	0	**0**	**0,0%**
Dubai, EAU	*Eretmochelys imbricata*	**9**	**9**	0	0	0	**0**	**0,0%**
Dubai, EAU	*Lepidochelys olivacea*	**1**	**1**	0	0	0	**0**	**0,0%**
Ghana, Western Africa	*Dermochelys coriacea*	**17**	**17**	0	0	2	**2**	**11,8%**
Ghana, Western Africa	*Lepidochelys olivacea*	**9**	**9**	0	0	1	**1**	**11,1%**
Hawaii, USA, Northern Pacific	*Chelonia mydas agassizi*	**51**	**15**	33	37	32	**50**	**98,0%**
Masirah Island, Oman	*Caretta caretta*	**37**	**34**	0	0	0	**0**	**0,0%**
Masirah Island, Oman	*Lepidochelys olivacea*	**1**	**1**	0	0	0	**0**	**0,0%**
North Pacific, California	*Caretta caretta*	**1**	**1**	0	0	0	**0**	**0,0%**
Northen Cyprus, Mediterranean	*Caretta caretta*	**24**	**24**	0	3	2	**5**	**20,8%**
Northen Cyprus, Mediterranean	*Chelonia mydas*	**26**	**26**	0	3	2	**5**	**19,2%**
Ostional, Costa Rican Pacific coast	*Dermochelys coriacea*	**1**	**1**	1	0	1	**1**	**100,0%**
Ostional, Costa Rican Pacific coast	*Lepidochelys olivacea*	**9**	**9**	0	1	1	**2**	**22,2%**
Portugal, Northern Atlantic	*Caretta caretta*	**1**	**1**	0	0	1	**1**	**100,0%**
Portugal, Northern Atlantic	*Chelonia mydas*	**2**	**2**	1	1	1	**2**	**100,0%**
Portugal, Northern Atlantic	*Dermochelys coriacea*	**2**	**2**	0	0	1	**1**	**50,0%**
Principe Island, Western Africa	*Chelonia mydas*	**14**	**7**	7	10	10	**14**	**100,0%**
Principe Island, Western Africa	*Eretmochelys imbricata*	**4**	**4**	3	2	4	**4**	**100,0%**
Puerto Rico, Caribbean	*Chelonia mydas*	**6**	**6**	0	2	0	**2**	**33,3%**
Puerto Rico, Caribbean	*Eretmochelys imbricata*	**5**	**5**	0	0	0	**0**	**0,0%**
Qaru Island, Kuwait, Persian Gulf	*Chelonia mydas*	**8**	**8**	0	0	0	**0**	**0,0%**
Qaru Island, Kuwait, Persian Gulf	*Eretmochelys imbricata*	**10**	**10**	1	3	0	**3**	**30,0%**
San Diego Bay, California, Pacific	*Chelonia mydas*	**28**	**27**	0	6	2	**8**	**28,6%**
Tortuguero, Costa Rican Caribbean coast	*Chelonia mydas*	**99**	**99**	0	7	0	**7**	**7,1%**
Turks & Caicos Islands, Caribbean Sea	*Chelonia mydas*	**25**	**15**	11	18	14	**23**	**92,0%**
**Total for sea turtle DNA extracts**		**398**	**337**	**58**	**93**	**75**	**132**	**33,2%**
**Tortoises and terrapins (non sea turtles)**								
Denmark, Copenhagen Zoo	*Agroniemus horsfieldii* (tortoise)	**1**		0	0	0	**0**	**0,0%**
Denmark, Copenhagen Zoo	*Carettochelys insculpta* (terrapin)	**1**		0	0	0	**0**	**0,0%**
Denmark, Copenhagen Zoo	*Cuora amboinensis* (semi-terrapin)	**1**		0	0	0	**0**	**0,0%**
Denmark, Copenhagen Zoo	*Geochelone carbonaria* (tortoise)	**1**		0	0	0	**0**	**0,0%**
Denmark, Copenhagen Zoo	*Geochelone pardalis* (tortoise)	**1**		0	0	0	**0**	**0,0%**
Denmark, Copenhagen Zoo	*Geochelone radiata* (tortoise)	**1**		0	0	0	**0**	**0,0%**
Denmark, Copenhagen Zoo	*Indotestudo elongata* (tortoise)	**2**		0	0	0	**0**	**0,0%**
Denmark, Copenhagen Zoo	*Podocnemis unifilis* (terrapin)	**2**		0	0	0	**0**	**0,0%**
Denmark, Copenhagen Zoo	*Testudo greca* (tortoise)	**1**		0	0	0	**0**	**0,0%**

Note: Please notice that the percentage (%) of CFPHV has been calculated upon the number of DNA extracts analysed and not the total number of individual turtles as estimated in Table 2, therefore the increase in the final percentage value
