# Supplementary material for: Global distribution of Chelonid fibropapilloma-associated herpesvirus among clinically healthy sea turtles
Source: BMC Evol Biol. 2014 Oct 25;14:206. doi: 10.1186/s12862-014-0206-z (PMC4219010; doi:10.1186/s12862-014-0206-z)
Supplement: Additional file 2: — Detailed number of turtle species individuals analysed for the CFPHV DNA detection (see Additional file 1 ); and Description of data. Listed results per turtle species as either CFPHV positive in FP exhibiting turtles (orange colour) and clinically healthy carrying CFPHV DNA (yellow colour), or CFPHV free (CFPHV negative) uniquely for clinically healthy turtles (green colour). [file 12862_2014_206_MOESM2_ESM.pdf]

Species common name	Sea turtle population or location of sample source	Number of turtle individuals analysed	Results from PCR CFPHV detection by type of tissue			Total turtles detected CFPHV positive	% of CFPHV detection by turtle species
			FP exhibiting	healthy + CFPHV	CFPHV free		
Chelonia mydas	Denmark, Danmarks Aquarium	**1**	0	0	1	**0**	**0,0%**
Chelonia mydas	Dubai, EAU	**2**	0	0	2	**0**	**0,0%**
Chelonia mydas	Northen Cyprus, Mediterranean	**26**	0	5	21	**5**	**19,2%**
Chelonia mydas	Portugal, Northern Atlantic	**2**	0	2	0	**2**	**100,0%**
Chelonia mydas	Principe Island, Western Africa	**7**	7	0	0	**7**	**100,0%**
Chelonia mydas	Puerto Rico, Caribbean	**6**	0	2	4	**2**	**33,3%**
Chelonia mydas	Qaru Island, Kuwait, Persian Gulf	**8**	0	0	8	**0**	**0,0%**
Chelonia mydas	San Diego Bay, California, Pacific	**27**	0	8	19	**8**	**29,6%**
Chelonia mydas	Tortuguero, Costa Rican Caribbean coast	**99**	0	7	92	**7**	**7,1%**
Chelonia mydas	Turks & Caicos Islands, Caribbean Sea	**15**	15	0	0	**15**	**100,0%**
Chelonia mydas agassizi	Hawaii, USA, Northern Pacific	**15**	15	0	0	**15**	**100,0%**
**Total per Green (*Cm*) species**		**208**	**37**	**24**	**147**	**61**	**29,3%**
Caretta caretta	Denmark, Danmarks Aquarium	**1**	0	1	0	**1**	**100,0%**
Caretta caretta	Masirah Island, Oman	**34**	0	0	34	**0**	**0,0%**
Caretta caretta	North Pacific, California	**1**	0	0	1	**0**	**0,0%**
Caretta caretta	Northen Cyprus, Mediterranean	**24**	0	5	19	**5**	**20,8%**
Caretta caretta	Portugal, Northern Atlantic	**1**	0	1	0	**1**	**100,0%**
**Total per Loggerhead (*Cc*) species**		**61**	**0**	**7**	**54**	**7**	**11,5%**
Dermochelys coriacea	Ghana, Western Africa	**17**	0	2	15	**2**	**11,8%**
Dermochelys coriacea	Ostional, Costa Rican Pacific coast	**1**	0	1	0	**1**	**100,0%**
Dermochelys coriacea	Portugal, Northern Atlantic	**2**	0	1	1	**1**	**50,0%**
**Total per Leatherback (*Dc*) species**		**20**	**0**	**4**	**16**	**4**	**20,0%**
Eretmochelys imbricata	Dubai, EAU	**9**	0	0	9	**0**	**0,0%**
Eretmochelys imbricata	Principe Island, Western Africa	**4**	0	4	0	**4**	**100,0%**
Eretmochelys imbricata	Puerto Rico, Caribbean	**5**	0	0	5	**0**	**0,0%**
Eretmochelys imbricata	Qaru Island, Kuwait, Persian Gulf	**10**	0	3	7	**3**	**30,0%**
**Total per Hawksbill (*Ei*) species**		**28**	**0**	**7**	**21**	**7**	**25,0%**
Lepidochelys olivacea	Dubai, EAU	**1**	0	0	1	**0**	**0,0%**
Lepidochelys olivacea	Ghana, Western Africa	**9**	0	1	8	**1**	**11,1%**
Lepidochelys olivacea	Masirah Island, Oman	**1**	0	0	1	**0**	**0,0%**
Lepidochelys olivacea	Ostional, Costa Rican Pacific coast	**9**	0	2	7	**2**	**22,2%**
**Total per Olive Ridley (*Lo*) species**		**20**	**0**	**3**	**17**	**3**	**15,0%**
**Grand total sea turtle individuals analysed**		**337**	**37**	**45**	**255**	**82**	**24,3%**
