# Supplementary material for: Global distribution of Chelonid fibropapilloma-associated herpesvirus among clinically healthy sea turtles
Source: BMC Evol Biol. 2014 Oct 25;14:206. doi: 10.1186/s12862-014-0206-z (PMC4219010; doi:10.1186/s12862-014-0206-z)
Supplement: Additional file 3: — Haplotype and amino acid sequences list; and Description of data. Detailed list of haplotype DNA sequences and amino acid variants found for the highly conserved regions for three short-fragments markers (UL18, UL22 and UL27) within the CFPHV genome. [file 12862_2014_206_MOESM3_ESM.pdf]

Haplotype and amino acid sequences list:

Detailed list of haplotype DNA sequences and amino acid variants found for the highly conserved regions for three short-fragments markers (UL18, UL22 and UL27) within the CFPHV genome.

UL18

<UL18 haplotype1, most common haplotype found all over the world; 98 nucleotides, 46 samples>
CCGTTAACCCACTCGAAGGGTCCCGTGTTTCGAATTTTGCAGAGCGCCGCGCTGGTACTCATTTCCAAAACGGAGAGGATTACCCGGCGGGGTTCCAC
<UL18 amino acid sequence variable sites, only functional site variantion Y/C>
RRLPAVIFAVEPRRVILSVLEMSTSAAL**Y**KIRNTGPFEWVNGDRLALVPP

<UL18 haplotype2, found only in turtle from Turks and Caicos and unique individual from Portugal; 98 nucleotides, 12 samples>
CCGTTGACCCATTCGAAGGGTCCCGTGTTTCGAATTTTGTAGAGCGCCGCGCTGGTACTCATTTCCAAGACGGAGAGAATTACCCGGCGGGGTTCCAC
<UL18 amino acid sequence variable sites, only functional site variantion Y/C>
RRLPAVIFAVEPRRVILSVLEMSTSAAL**C**KIRNTGPFEWVNGDRLALVPP

UL22

<UL22 haplotype1, most common haplotype found all over the world; 132 nucleotides, 82 samples>
ATCGCTGGTCATAGACTTGCCGTGTTTGCGGGTTTAACCAAAGATTCAGTGCGCACCAGCACGACGTTTGGTTCGTGTTTCGCAAAAGTATCAGATCCGAACTCTACAATATGGGTCGGAGGAAAGGGCGTT
<UL22 amino acid sequence variable sites, only functional site variantion D/E>
APQNATPFPPTHIVEFGS**D**TFAKHEPNVVLVRTESLVKPANTASLLTSDLFYPVQFE

<UL22 haplotype2, found only in turtle from Turks and Caicos and unique individual from Portugal; 132 nucleotides, 13 samples>
ATCGCTGGTCATAGACTTGCCGTGTTTGCGGGTTTAACCAAGGATTCAGTGCGCACCAGCACGACGTTTGGTTCGTGTTTCGCAAAAGTTTCAGATCCGAACTCTACAATATGGGTCGGAGGAAAGGGCGTT
<UL22 amino acid sequence variable sites, only functional site variantion D/E>
APQNATPFPPTHIVEFGS**E**TFAKHEPNVVLVRTESLVKPANTASLLTSDLFYPVQFE

UL27

<UL27 haplotype1, most common haplotype found all over the world; 144 nucleotides, 50 samples>
CCTCCGGATGGTCGCTGGCCAAAGAAAAACTATCTAACGTCAAAGACAACTTTACGGACGCTAAAAATAAAATGATGAAATCTTTACGCCACCGCGGAAACGGATACGCTCCCGTTTCGACGAGCATGGCCAGTATGTATCTAG
<UL27 amino acid sequence variable sites, only functional site variantions K/E and T/M>
KKAASSSGWSLAK**K**KLSNVKDNFTDAKNKMMKSLRHRGNGYAPVSTS**T**ASMYLDDELSTYSQ

<UL27 haplotype2, 144 nucleotides, 7 samples>
CCTCCGGATGGTCGCTGGCCAAGGAAAAACTATCTAACGTCAAAGACAACTTTACGGACGCTAAAAATAAAATGATGAAATCTTTACGCCACCGCGGAAACGGATACGCTCCCGTTTCGACGAGCATGGCCAGTATGTATCTAG
<UL27 amino acid sequence variable sites, only functional site variantions K/E and T/M>
KKAASSSGWSLAK**E**KLSNVKDNFTDAKNKMMKSLRHRGNGYAPVSTS**T**ASMYLDDELSTYSQ

<UL27 haplotype3, 144 nucleotides, 18 samples>
CCTCCGGATGGTCGCTGGCCAAAGAAAAACTATCTAACGTCAAAGACAACTTTACGGACGCTAAAAATAAAATGATGAAATCTTTACGCCACCGCGGAAACGGATACGCTCCCGTTTCGACGAGCACGGCCAGTATGTATCTAG
<UL27 amino acid sequence variable sites, only functional site variantions K/E and T/M>
KKAASSSGWSLAK**K**KLSNVKDNFTDAKNKMMKSLRHRGNGYAPVSTS**M**ASMYLDDELSTYSQ

<UL18_common_haplotype1>

>Ei-PiT-85_UL18_2R.ab1
CCGTTAACCCACTCGAAGGGTCCCGTGTTTCGAATTTTGCAGAGCGCCGCGCTGGTACTCATTTCCAAAACGGAGAGGATTACCCGGCGGGGTTCCAC
>Cc-DkT-01_UL18_R.ab1
CCGTTAACCCACTCGAAGGGTCCCGTGTTTCGAATTTTGCAGAGCGCCGCGCTGGTACTCATTTCCAAAACGGAGAGGATTACCCGGCGGGGTTCCAC
>Cm-HaFP-1RMOUTH_UL18_R.ab1
CCGTTAACCCACTCGAAGGGTCCCGTGTTTCGAATTTTGCAGAGCGCCGCGCTGGTACTCATTTCCAAAACGGAGAGGATTACCCGGCGGGGTTCCAC
>Cm-HaFP-2LFF_UL18_R.ab1
CCGTTAACCCACTCGAAGGGTCCCGTGTTTCGAATTTTGCAGAGCGCCGCGCTGGTACTCATTTCCAAAACGGAGAGGATTACCCGGCGGGGTTCCAC
>Cm-HaFP-2LHF_UL18_R.ab1
CCGTTAACCCACTCGAAGGGTCCCGTGTTTCGAATTTTGCAGAGCGCCGCGCTGGTACTCATTTCCAAAACGGAGAGGATTACCCGGCGGGGTTCCAC
>Cm-HaFP-3NECK_UL18_R.ab1
CCGTTAACCCACTCGAAGGGTCCCGTGTTTCGAATTTTGCAGAGCGCCGCGCTGGTACTCATTTCCAAAACGGAGAGGATTACCCGGCGGGGTTCCAC
>Cm-HaFP-4NECK_UL18_R.ab1
CCGTTAACCCACTCGAAGGGTCCCGTGTTTCGAATTTTGCAGAGCGCCGCGCTGGTACTCATTTCCAAAACGGAGAGGATTACCCGGCGGGGTTCCAC
>Cm-HaFP-5LFF_UL18_R.ab1
CCGTTAACCCACTCGAAGGGTCCCGTGTTTCGAATTTTGCAGAGCGCCGCGCTGGTACTCATTTCCAAAACGGAGAGGATTACCCGGCGGGGTTCCAC
>Cm-HaFP-6NECK1_UL18_R.ab1
CCGTTAACCCACTCGAAGGGTCCCGTGTTTCGAATTTTGCAGAGCGCCGCGCTGGTACTCATTTCCAAAACGGAGAGGATTACCCGGCGGGGTTCCAC
>Cm-HaFP-6NECK_UL18_R.ab1
CCGTTAACCCACTCGAAGGGTCCCGTGTTTCGAATTTTGCAGAGCGCCGCGCTGGTACTCATTTCCAAAACGGAGAGGATTACCCGGCGGGGTTCCAC
>Cm-HaFP-6RFLIPPER_UL18_R.ab1
CCGTTAACCCACTCGAAGGGTCCCGTGTTTCGAATTTTGCAGAGCGCCGCGCTGGTACTCATTTCCAAAACGGAGAGGATTACCCGGCGGGGTTCCAC
>Cm-HaFP-7LEYE_UL18_R.ab1
CCGTTAACCCACTCGAAGGGTCCCGTGTTTCGAATTTTGCAGAGCGCCGCGCTGGTACTCATTTCCAAAACGGAGAGGATTACCCGGCGGGGTTCCAC
>Cm-HaFP-7LHF_UL18_R.ab1
CCGTTAACCCACTCGAAGGGTCCCGTGTTTCGAATTTTGCAGAGCGCCGCGCTGGTACTCATTTCCAAAACGGAGAGGATTACCCGGCGGGGTTCCAC
>Cm-HaFP-7REYE_UL18_R.ab1
CCGTTAACCCACTCGAAGGGTCCCGTGTTTCGAATTTTGCAGAGCGCCGCGCTGGTACTCATTTCCAAAACGGAGAGGATTACCCGGCGGGGTTCCAC
>Cm-HaFP-8LFF_UL18_R.ab1
CCGTTAACCCACTCGAAGGGTCCCGTGTTTCGAATTTTGCAGAGCGCCGCGCTGGTACTCATTTCCAAAACGGAGAGGATTACCCGGCGGGGTTCCAC
>Cm-HaFP-8MAXILLA_UL18_R.ab1
CCGTTAACCCACTCGAAGGGTCCCGTGTTTCGAATTTTGCAGAGCGCCGCGCTGGTACTCATTTCCAAAACGGAGAGGATTACCCGGCGGGGTTCCAC
>Cm-HaFP-8RHF_UL18_R.ab1
CCGTTAACCCACTCGAAGGGTCCCGTGTTTCGAATTTTGCAGAGCGCCGCGCTGGTACTCATTTCCAAAACGGAGAGGATTACCCGGCGGGGTTCCAC
>Cm-HaFP-9LFF_UL18_R.ab1
CCGTTAACCCACTCGAAGGGTCCCGTGTTTCGAATTTTGCAGAGCGCCGCGCTGGTACTCATTTCCAAAACGGAGAGGATTACCCGGCGGGGTTCCAC
>Cm-HaFP-9RFF_UL18_R.ab1
CCGTTAACCCACTCGAAGGGTCCCGTGTTTCGAATTTTGCAGAGCGCCGCGCTGGTACTCATTTCCAAAACGGAGAGGATTACCCGGCGGGGTTCCAC
>Cm-HaFP-10LFF_UL18_R.ab1
CCGTTAACCCACTCGAAGGGTCCCGTGTTTCGAATTTTGCAGAGCGCCGCGCTGGTACTCATTTCCAAAACGGAGAGGATTACCCGGCGGGGTTCCAC
>Cm-HaFP-10LHF_UL18_R.ab1
CCGTTAACCCACTCGAAGGGTCCCGTGTTTCGAATTTTGCAGAGCGCCGCGCTGGTACTCATTTCCAAAACGGAGAGGATTACCCGGCGGGGTTCCAC
>Cm-HaFP-10RFF_UL18_R.ab1
CCGTTAACCCACTCGAAGGGTCCCGTGTTTCGAATTTTGCAGAGCGCCGCGCTGGTACTCATTTCCAAAACGGAGAGGATTACCCGGCGGGGTTCCAC
>Cm-HaFP-11NECK_UL18_R.ab1
CCGTTAACCCACTCGAAGGGTCCCGTGTTTCGAATTTTGCAGAGCGCCGCGCTGGTACTCATTTCCAAAACGGAGAGGATTACCCGGCGGGGTTCCAC
>Cm-HaFP-11RHF_UL18_R.ab1
CCGTTAACCCACTCGAAGGGTCCCGTGTTTCGAATTTTGCAGAGCGCCGCGCTGGTACTCATTTCCAAAACGGAGAGGATTACCCGGCGGGGTTCCAC
>Cm-HaFP-12LFF_UL18_R.ab1
CCGTTAACCCACTCGAAGGGTCCCGTGTTTCGAATTTTGCAGAGCGCCGCGCTGGTACTCATTTCCAAAACGGAGAGGATTACCCGGCGGGGTTCCAC
>Cm-HaFP-12LHF_UL18_R.ab1
CCGTTAACCCACTCGAAGGGTCCCGTGTTTCGAATTTTGCAGAGCGCCGCGCTGGTACTCATTTCCAAAACGGAGAGGATTACCCGGCGGGGTTCCAC
>Cm-HaFP-12TAIL_UL18_R.ab1
CCGTTAACCCACTCGAAGGGTCCCGTGTTTCGAATTTTGCAGAGCGCCGCGCTGGTACTCATTTCCAAAACGGAGAGGATTACCCGGCGGGGTTCCAC
>Cm-HaFP-14RHF_UL18_R.ab1
CCGTTAACCCACTCGAAGGGTCCCGTGTTTCGAATTTTGCAGAGCGCCGCGCTGGTACTCATTTCCAAAACGGAGAGGATTACCCGGCGGGGTTCCAC
>Cm-HaFP-14TAIL_UL18_R.ab1
CCGTTAACCCACTCGAAGGGTCCCGTGTTTCGAATTTTGCAGAGCGCCGCGCTGGTACTCATTTCCAAAACGGAGAGGATTACCCGGCGGGGTTCCAC
>Cm-HaFP-15NECK_UL18_R.ab1
CCGTTAACCCACTCGAAGGGTCCCGTGTTTCGAATTTTGCAGAGCGCCGCGCTGGTACTCATTTCCAAAACGGAGAGGATTACCCGGCGGGGTTCCAC
>Cm-HaFP-15REYE_UL18_R.ab1
CCGTTAACCCACTCGAAGGGTCCCGTGTTTCGAATTTTGCAGAGCGCCGCGCTGGTACTCATTTCCAAAACGGAGAGGATTACCCGGCGGGGTTCCAC
>Cm-HaT-2_UL18_R.ab1
CCGTTAACCCACTCGAAGGGTCCCGTGTTTCGAATTTTGCAGAGCGCCGCGCTGGTACTCATTTCCAAAACGGAGAGGATTACCCGGCGGGGTTCCAC
>Cm-HaT-8_UL18_2R.ab1
CCGTTAACCCACTCGAAGGGTCCCGTGTTTCGAATTTTGCAGAGCGCCGCGCTGGTACTCATTTCCAAAACGGAGAGGATTACCCGGCGGGGTTCCAC
>Cm-HaT-11_UL18_R.ab1
CCGTTAACCCACTCGAAGGGTCCCGTGTTTCGAATTTTGCAGAGCGCCGCGCTGGTACTCATTTCCAAAACGGAGAGGATTACCCGGCGGGGTTCCAC
>Cm-HaT-14_UL18_R.ab1
CCGTTAACCCACTCGAAGGGTCCCGTGTTTCGAATTTTGCAGAGCGCCGCGCTGGTACTCATTTCCAAAACGGAGAGGATTACCCGGCGGGGTTCCAC
>Cm-PiFF-80_UL18_2R.ab1
CCGTTAACCCACTCGAAGGGTCCCGTGTTTCGAATTTTGCAGAGCGCCGCGCTGGTACTCATTTCCAAAACGGAGAGGATTACCCGGCGGGGTTCCAC
>Cm-PiFP-04_UL18_R.ab1
CCGTTAACCCACTCGAAGGGTCCCGTGTTTCGAATTTTGCAGAGCGCCGCGCTGGTACTCATTTCCAAAACGGAGAGGATTACCCGGCGGGGTTCCAC
>Cm-PiFP-51_UL18_R.ab1
CCGTTAACCCACTCGAAGGGTCCCGTGTTTCGAATTTTGCAGAGCGCCGCGCTGGTACTCATTTCCAAAACGGAGAGGATTACCCGGCGGGGTTCCAC
>Cm-PiFP-86_UL18_R.ab1
CCGTTAACCCACTCGAAGGGTCCCGTGTTTCGAATTTTGCAGAGCGCCGCGCTGGTACTCATTTCCAAAACGGAGAGGATTACCCGGCGGGGTTCCAC
>Cm-PiFP-92_UL18_R.ab1
CCGTTAACCCACTCGAAGGGTCCCGTGTTTCGAATTTTGCAGAGCGCCGCGCTGGTACTCATTTCCAAAACGGAGAGGATTACCCGGCGGGGTTCCAC
>Cm-PiT-20_UL18_R.ab1
CCGTTAACCCACTCGAAGGGTCCCGTGTTTCGAATTTTGCAGAGCGCCGCGCTGGTACTCATTTCCAAAACGGAGAGGATTACCCGGCGGGGTTCCAC
>Cm-PiT-51_UL18_R.ab1
CCGTTAACCCACTCGAAGGGTCCCGTGTTTCGAATTTTGCAGAGCGCCGCGCTGGTACTCATTTCCAAAACGGAGAGGATTACCCGGCGGGGTTCCAC
>Dc-OsT-01_UL18_2R.ab1
CCGTTAACCCACTCGAAGGGTCCCGTGTTTCGAATTTTGCAGAGCGCCGCGCTGGTACTCATTTCCAAAACGGAGAGGATTACCCGGCGGGGTTCCAC
>Ei-KuT-19_UL18_R.ab1
CCGTTAACCCACTCGAAGGGTCCCGTGTTTCGAATTTTGCAGAGCGCCGCGCTGGTACTCATTTCCAAAACGGAGAGGATTACCCGGCGGGGTTCCAC
>Ei-PiT-46_UL18_R.ab1
CCGTTAACCCACTCGAAGGGTCCCGTGTTTCGAATTTTGCAGAGCGCCGCGCTGGTACTCATTTCCAAAACGGAGAGGATTACCCGGCGGGGTTCCAC
>Ei-PiT-71_UL18_R.ab1
CCGTTAACCCACTCGAAGGGTCCCGTGTTTCGAATTTTGCAGAGCGCCGCGCTGGTACTCATTTCCAAAACGGAGAGGATTACCCGGCGGGGTTCCAC

<UL18_T&C_haplotype2>

>Cm-TCFP-15_UL18_R.ab1
CCGTTGACCCATTCGAAGGGTCCCGTGTTTCGAATTTTGTAGAGCGCCGCGCTGGTACTCATTTCCAAGACGGAGAGAATTACCCGGCGGGGTTCCAC
>Cm-PoT-01_UL18_2R.ab1
CCGTTGACCCATTCGAAGGGTCCCGTGTTTCGAATTTTGTAGAGCGCCGCGCTGGTACTCATTTCCAAGACGGAGAGAATTACCCGGCGGGGTTCCAC
>Cm-TCFP-1cloaca2_UL18_R.ab1
CCGTTGACCCATTCGAAGGGTCCCGTGTTTCGAATTTTGTAGAGCGCCGCGCTGGTACTCATTTCCAAGACGGAGAGAATTACCCGGCGGGGTTCCAC
>Cm-TCFP-1cloaca4_UL18_R.ab1
CCGTTGACCCATTCGAAGGGTCCCGTGTTTCGAATTTTGTAGAGCGCCGCGCTGGTACTCATTTCCAAGACGGAGAGAATTACCCGGCGGGGTTCCAC
>Cm-TCFP-3_UL18_R.ab1
CCGTTGACCCATTCGAAGGGTCCCGTGTTTCGAATTTTGTAGAGCGCCGCGCTGGTACTCATTTCCAAGACGGAGAGAATTACCCGGCGGGGTTCCAC
>Cm-TCFP-4_UL18_R.ab1
CCGTTGACCCATTCGAAGGGTCCCGTGTTTCGAATTTTGTAGAGCGCCGCGCTGGTACTCATTTCCAAGACGGAGAGAATTACCCGGCGGGGTTCCAC
>Cm-TCFP-5_UL18_R.ab1
CCGTTGACCCATTCGAAGGGTCCCGTGTTTCGAATTTTGTAGAGCGCCGCGCTGGTACTCATTTCCAAGACGGAGAGAATTACCCGGCGGGGTTCCAC
>Cm-TCFP-8_UL18_R.ab1
CCGTTGACCCATTCGAAGGGTCCCGTGTTTCGAATTTTGTAGAGCGCCGCGCTGGTACTCATTTCCAAGACGGAGAGAATTACCCGGCGGGGTTCCAC
>Cm-TCFP-9_UL18_R.ab1
CCGTTGACCCATTCGAAGGGTCCCGTGTTTCGAATTTTGTAGAGCGCCGCGCTGGTACTCATTTCCAAGACGGAGAGAATTACCCGGCGGGGTTCCAC
>Cm-TCFP-12_UL18_R.ab1
CCGTTGACCCATTCGAAGGGTCCCGTGTTTCGAATTTTGTAGAGCGCCGCGCTGGTACTCATTTCCAAGACGGAGAGAATTACCCGGCGGGGTTCCAC
>Cm-TCFP-13_UL18_R.ab1
CCGTTGACCCATTCGAAGGGTCCCGTGTTTCGAATTTTGTAGAGCGCCGCGCTGGTACTCATTTCCAAGACGGAGAGAATTACCCGGCGGGGTTCCAC
>Cm-TCFP-14_UL18_R.ab1
CCGTTGACCCATTCGAAGGGTCCCGTGTTTCGAATTTTGTAGAGCGCCGCGCTGGTACTCATTTCCAAGACGGAGAGAATTACCCGGCGGGGTTCCAC

<UL22_common_haplotype1>

>Lo-OsT-07_UL22_R.ab1
ATCGCTGGTCATAGACTTGCCGTGTTTGCGGGTTTAACCAAAGATTCAGTGCGCACCAGCACGACGTTTGGTTCGTGTTTCGCAAAAGTATCAGATCCGAACTCTACAATATGGGTCGGAGGAAAGGGCGTT
>Cc-CyT-281_UL22_R.ab1
ATCGCTGGTCATAGACTTGCCGTGTTTGCGGGTTTAACCAAAGATTCAGTGCGCACCAGCACGACGTTTGGTTCGTGTTTCGCAAAAGTATCAGATCCGAACTCTACAATATGGGTCGGAGGAAAGGGCGTT
>Cc-CyT-288_UL22_R.ab1
ATCGCTGGTCATAGACTTGCCGTGTTTGCGGGTTTAACCAAAGATTCAGTGCGCACCAGCACGACGTTTGGTTCGTGTTTCGCAAAAGTATCAGATCCGAACTCTACAATATGGGTCGGAGGAAAGGGCGTT
>Cc-CyT-346_UL22_R.ab1
ATCGCTGGTCATAGACTTGCCGTGTTTGCGGGTTTAACCAAAGATTCAGTGCGCACCAGCACGACGTTTGGTTCGTGTTTCGCAAAAGTATCAGATCCGAACTCTACAATATGGGTCGGAGGAAAGGGCGTT
>Cma-SDT-05_UL22_R.ab1
ATCGCTGGTCATAGACTTGCCGTGTTTGCGGGTTTAACCAAAGATTCAGTGCGCACCAGCACGACGTTTGGTTCGTGTTTCGCAAAAGTATCAGATCCGAACTCTACAATATGGGTCGGAGGAAAGGGCGTT
>Cma-SDT-06_UL22_R.ab1
ATCGCTGGTCATAGACTTGCCGTGTTTGCGGGTTTAACCAAAGATTCAGTGCGCACCAGCACGACGTTTGGTTCGTGTTTCGCAAAAGTATCAGATCCGAACTCTACAATATGGGTCGGAGGAAAGGGCGTT
>Cma-SDT-07_UL22_R.ab1
ATCGCTGGTCATAGACTTGCCGTGTTTGCGGGTTTAACCAAAGATTCAGTGCGCACCAGCACGACGTTTGGTTCGTGTTTCGCAAAAGTATCAGATCCGAACTCTACAATATGGGTCGGAGGAAAGGGCGTT
>Cma-SDT-08_UL22_R.ab1
ATCGCTGGTCATAGACTTGCCGTGTTTGCGGGTTTAACCAAAGATTCAGTGCGCACCAGCACGACGTTTGGTTCGTGTTTCGCAAAAGTATCAGATCCGAACTCTACAATATGGGTCGGAGGAAAGGGCGTT
>Cma-SDT-10_UL22_R.ab1
ATCGCTGGTCATAGACTTGCCGTGTTTGCGGGTTTAACCAAAGATTCAGTGCGCACCAGCACGACGTTTGGTTCGTGTTTCGCAAAAGTATCAGATCCGAACTCTACAATATGGGTCGGAGGAAAGGGCGTT
>Cma-SDT-11_UL22_R.ab1
ATCGCTGGTCATAGACTTGCCGTGTTTGCGGGTTTAACCAAAGATTCAGTGCGCACCAGCACGACGTTTGGTTCGTGTTTCGCAAAAGTATCAGATCCGAACTCTACAATATGGGTCGGAGGAAAGGGCGTT
>Cm-CyT-156_UL22_R.ab1
ATCGCTGGTCATAGACTTGCCGTGTTTGCGGGTTTAACCAAAGATTCAGTGCGCACCAGCACGACGTTTGGTTCGTGTTTCGCAAAAGTATCAGATCCGAACTCTACAATATGGGTCGGAGGAAAGGGCGTT
>Cm-CyT-226_UL22_R.ab1
ATCGCTGGTCATAGACTTGCCGTGTTTGCGGGTTTAACCAAAGATTCAGTGCGCACCAGCACGACGTTTGGTTCGTGTTTCGCAAAAGTATCAGATCCGAACTCTACAATATGGGTCGGAGGAAAGGGCGTT
>Cm-CyT-228_UL22_R.ab1
ATCGCTGGTCATAGACTTGCCGTGTTTGCGGGTTTAACCAAAGATTCAGTGCGCACCAGCACGACGTTTGGTTCGTGTTTCGCAAAAGTATCAGATCCGAACTCTACAATATGGGTCGGAGGAAAGGGCGTT
>Cm-HaFB-12LHF_UL22_R.ab1
ATCGCTGGTCATAGACTTGCCGTGTTTGCGGGTTTAACCAAAGATTCAGTGCGCACCAGCACGACGTTTGGTTCGTGTTTCGCAAAAGTATCAGATCCGAACTCTACAATATGGGTCGGAGGAAAGGGCGTT
>Cm-HaFP-3LHF_UL22_R.ab1
ATCGCTGGTCATAGACTTGCCGTGTTTGCGGGTTTAACCAAAGATTCAGTGCGCACCAGCACGACGTTTGGTTCGTGTTTCGCAAAAGTATCAGATCCGAACTCTACAATATGGGTCGGAGGAAAGGGCGTT
>Cm-HaFP-5LFF_UL22_R.ab1
ATCGCTGGTCATAGACTTGCCGTGTTTGCGGGTTTAACCAAAGATTCAGTGCGCACCAGCACGACGTTTGGTTCGTGTTTCGCAAAAGTATCAGATCCGAACTCTACAATATGGGTCGGAGGAAAGGGCGTT
>Cm-HaFP-6NECK1_UL22_R.ab1
ATCGCTGGTCATAGACTTGCCGTGTTTGCGGGTTTAACCAAAGATTCAGTGCGCACCAGCACGACGTTTGGTTCGTGTTTCGCAAAAGTATCAGATCCGAACTCTACAATATGGGTCGGAGGAAAGGGCGTT
>Cm-HaFP-6NECK_UL22_R.ab1
ATCGCTGGTCATAGACTTGCCGTGTTTGCGGGTTTAACCAAAGATTCAGTGCGCACCAGCACGACGTTTGGTTCGTGTTTCGCAAAAGTATCAGATCCGAACTCTACAATATGGGTCGGAGGAAAGGGCGTT
>Cm-HaFP-6RFLIPPER_UL22_R.ab1
ATCGCTGGTCATAGACTTGCCGTGTTTGCGGGTTTAACCAAAGATTCAGTGCGCACCAGCACGACGTTTGGTTCGTGTTTCGCAAAAGTATCAGATCCGAACTCTACAATATGGGTCGGAGGAAAGGGCGTT
>Cm-HaFP-7LEYE_UL22_R.ab1
ATCGCTGGTCATAGACTTGCCGTGTTTGCGGGTTTAACCAAAGATTCAGTGCGCACCAGCACGACGTTTGGTTCGTGTTTCGCAAAAGTATCAGATCCGAACTCTACAATATGGGTCGGAGGAAAGGGCGTT
>Cm-HaFP-7LHF_UL22_R.ab1
ATCGCTGGTCATAGACTTGCCGTGTTTGCGGGTTTAACCAAAGATTCAGTGCGCACCAGCACGACGTTTGGTTCGTGTTTCGCAAAAGTATCAGATCCGAACTCTACAATATGGGTCGGAGGAAAGGGCGTT
>Cm-HaFP-7REYE_UL22_R.ab1
ATCGCTGGTCATAGACTTGCCGTGTTTGCGGGTTTAACCAAAGATTCAGTGCGCACCAGCACGACGTTTGGTTCGTGTTTCGCAAAAGTATCAGATCCGAACTCTACAATATGGGTCGGAGGAAAGGGCGTT
>Cm-HaFP-8LFF_UL22_R.ab1
ATCGCTGGTCATAGACTTGCCGTGTTTGCGGGTTTAACCAAAGATTCAGTGCGCACCAGCACGACGTTTGGTTCGTGTTTCGCAAAAGTATCAGATCCGAACTCTACAATATGGGTCGGAGGAAAGGGCGTT
>Cm-HaFP-8MAXILLA_UL22_R.ab1
ATCGCTGGTCATAGACTTGCCGTGTTTGCGGGTTTAACCAAAGATTCAGTGCGCACCAGCACGACGTTTGGTTCGTGTTTCGCAAAAGTATCAGATCCGAACTCTACAATATGGGTCGGAGGAAAGGGCGTT
>Cm-HaFP-8RHF_UL22_R.ab1
ATCGCTGGTCATAGACTTGCCGTGTTTGCGGGTTTAACCAAAGATTCAGTGCGCACCAGCACGACGTTTGGTTCGTGTTTCGCAAAAGTATCAGATCCGAACTCTACAATATGGGTCGGAGGAAAGGGCGTT
>Cm-HaFP-9CLOACA_UL22_R.ab1
ATCGCTGGTCATAGACTTGCCGTGTTTGCGGGTTTAACCAAAGATTCAGTGCGCACCAGCACGACGTTTGGTTCGTGTTTCGCAAAAGTATCAGATCCGAACTCTACAATATGGGTCGGAGGAAAGGGCGTT
>Cm-HaFP-9LFF_UL22_R.ab1
ATCGCTGGTCATAGACTTGCCGTGTTTGCGGGTTTAACCAAAGATTCAGTGCGCACCAGCACGACGTTTGGTTCGTGTTTCGCAAAAGTATCAGATCCGAACTCTACAATATGGGTCGGAGGAAAGGGCGTT
>Cm-HaFP-9RFF_UL22_R.ab1
ATCGCTGGTCATAGACTTGCCGTGTTTGCGGGTTTAACCAAAGATTCAGTGCGCACCAGCACGACGTTTGGTTCGTGTTTCGCAAAAGTATCAGATCCGAACTCTACAATATGGGTCGGAGGAAAGGGCGTT
>Cm-HaFP-10LFF_UL22_R.ab1
ATCGCTGGTCATAGACTTGCCGTGTTTGCGGGTTTAACCAAAGATTCAGTGCGCACCAGCACGACGTTTGGTTCGTGTTTCGCAAAAGTATCAGATCCGAACTCTACAATATGGGTCGGAGGAAAGGGCGTT
>Cm-HaFP-10LHF_UL22_R.ab1
ATCGCTGGTCATAGACTTGCCGTGTTTGCGGGTTTAACCAAAGATTCAGTGCGCACCAGCACGACGTTTGGTTCGTGTTTCGCAAAAGTATCAGATCCGAACTCTACAATATGGGTCGGAGGAAAGGGCGTT
>Cm-HaFP-10RFF_UL22_R.ab1
ATCGCTGGTCATAGACTTGCCGTGTTTGCGGGTTTAACCAAAGATTCAGTGCGCACCAGCACGACGTTTGGTTCGTGTTTCGCAAAAGTATCAGATCCGAACTCTACAATATGGGTCGGAGGAAAGGGCGTT
>Cm-HaFP-11LHF_UL22_R.ab1
ATCGCTGGTCATAGACTTGCCGTGTTTGCGGGTTTAACCAAAGATTCAGTGCGCACCAGCACGACGTTTGGTTCGTGTTTCGCAAAAGTATCAGATCCGAACTCTACAATATGGGTCGGAGGAAAGGGCGTT
>Cm-HaFP-11NECK_UL22_R.ab1
ATCGCTGGTCATAGACTTGCCGTGTTTGCGGGTTTAACCAAAGATTCAGTGCGCACCAGCACGACGTTTGGTTCGTGTTTCGCAAAAGTATCAGATCCGAACTCTACAATATGGGTCGGAGGAAAGGGCGTT
>Cm-HaFP-11RHF_UL22_R.ab1
ATCGCTGGTCATAGACTTGCCGTGTTTGCGGGTTTAACCAAAGATTCAGTGCGCACCAGCACGACGTTTGGTTCGTGTTTCGCAAAAGTATCAGATCCGAACTCTACAATATGGGTCGGAGGAAAGGGCGTT
>Cm-HaFP-12LFF_UL22_R.ab1
ATCGCTGGTCATAGACTTGCCGTGTTTGCGGGTTTAACCAAAGATTCAGTGCGCACCAGCACGACGTTTGGTTCGTGTTTCGCAAAAGTATCAGATCCGAACTCTACAATATGGGTCGGAGGAAAGGGCGTT
>Cm-HaFP-12TAIL_UL22_R.ab1
ATCGCTGGTCATAGACTTGCCGTGTTTGCGGGTTTAACCAAAGATTCAGTGCGCACCAGCACGACGTTTGGTTCGTGTTTCGCAAAAGTATCAGATCCGAACTCTACAATATGGGTCGGAGGAAAGGGCGTT
>Cm-HaFP-14NECK_UL22_R.ab1
ATCGCTGGTCATAGACTTGCCGTGTTTGCGGGTTTAACCAAAGATTCAGTGCGCACCAGCACGACGTTTGGTTCGTGTTTCGCAAAAGTATCAGATCCGAACTCTACAATATGGGTCGGAGGAAAGGGCGTT
>Cm-HaFP-14RHF_UL22_R.ab1
ATCGCTGGTCATAGACTTGCCGTGTTTGCGGGTTTAACCAAAGATTCAGTGCGCACCAGCACGACGTTTGGTTCGTGTTTCGCAAAAGTATCAGATCCGAACTCTACAATATGGGTCGGAGGAAAGGGCGTT
>Cm-HaFP-14TAIL_UL22_R.ab1
ATCGCTGGTCATAGACTTGCCGTGTTTGCGGGTTTAACCAAAGATTCAGTGCGCACCAGCACGACGTTTGGTTCGTGTTTCGCAAAAGTATCAGATCCGAACTCTACAATATGGGTCGGAGGAAAGGGCGTT
>Cm-HaFP-15NECK_UL22_R.ab1
ATCGCTGGTCATAGACTTGCCGTGTTTGCGGGTTTAACCAAAGATTCAGTGCGCACCAGCACGACGTTTGGTTCGTGTTTCGCAAAAGTATCAGATCCGAACTCTACAATATGGGTCGGAGGAAAGGGCGTT
>Cm-HaFP-15REYE_UL22_R.ab1
ATCGCTGGTCATAGACTTGCCGTGTTTGCGGGTTTAACCAAAGATTCAGTGCGCACCAGCACGACGTTTGGTTCGTGTTTCGCAAAAGTATCAGATCCGAACTCTACAATATGGGTCGGAGGAAAGGGCGTT
>Cm-HaFP-15RHF_UL22_R.ab1
ATCGCTGGTCATAGACTTGCCGTGTTTGCGGGTTTAACCAAAGATTCAGTGCGCACCAGCACGACGTTTGGTTCGTGTTTCGCAAAAGTATCAGATCCGAACTCTACAATATGGGTCGGAGGAAAGGGCGTT
>Cm-HaT-6_UL22_R.ab1
ATCGCTGGTCATAGACTTGCCGTGTTTGCGGGTTTAACCAAAGATTCAGTGCGCACCAGCACGACGTTTGGTTCGTGTTTCGCAAAAGTATCAGATCCGAACTCTACAATATGGGTCGGAGGAAAGGGCGTT
>Cm-HaT-7_UL22_R.ab1
ATCGCTGGTCATAGACTTGCCGTGTTTGCGGGTTTAACCAAAGATTCAGTGCGCACCAGCACGACGTTTGGTTCGTGTTTCGCAAAAGTATCAGATCCGAACTCTACAATATGGGTCGGAGGAAAGGGCGTT
>Cm-HaT-8_UL22_R.ab1
ATCGCTGGTCATAGACTTGCCGTGTTTGCGGGTTTAACCAAAGATTCAGTGCGCACCAGCACGACGTTTGGTTCGTGTTTCGCAAAAGTATCAGATCCGAACTCTACAATATGGGTCGGAGGAAAGGGCGTT
>Cm-HaT-9_UL22_R.ab1
ATCGCTGGTCATAGACTTGCCGTGTTTGCGGGTTTAACCAAAGATTCAGTGCGCACCAGCACGACGTTTGGTTCGTGTTTCGCAAAAGTATCAGATCCGAACTCTACAATATGGGTCGGAGGAAAGGGCGTT
>Cm-HaT-10_UL22_R.ab1
ATCGCTGGTCATAGACTTGCCGTGTTTGCGGGTTTAACCAAAGATTCAGTGCGCACCAGCACGACGTTTGGTTCGTGTTTCGCAAAAGTATCAGATCCGAACTCTACAATATGGGTCGGAGGAAAGGGCGTT
>Cm-HaT-11_UL22_R.ab1
ATCGCTGGTCATAGACTTGCCGTGTTTGCGGGTTTAACCAAAGATTCAGTGCGCACCAGCACGACGTTTGGTTCGTGTTTCGCAAAAGTATCAGATCCGAACTCTACAATATGGGTCGGAGGAAAGGGCGTT
>Cm-HaT-13_UL22_R.ab1
ATCGCTGGTCATAGACTTGCCGTGTTTGCGGGTTTAACCAAAGATTCAGTGCGCACCAGCACGACGTTTGGTTCGTGTTTCGCAAAAGTATCAGATCCGAACTCTACAATATGGGTCGGAGGAAAGGGCGTT
>Cm-HaT-14_UL22_R.ab1
ATCGCTGGTCATAGACTTGCCGTGTTTGCGGGTTTAACCAAAGATTCAGTGCGCACCAGCACGACGTTTGGTTCGTGTTTCGCAAAAGTATCAGATCCGAACTCTACAATATGGGTCGGAGGAAAGGGCGTT
>Cm-HaT-15_UL22_R.ab1
ATCGCTGGTCATAGACTTGCCGTGTTTGCGGGTTTAACCAAAGATTCAGTGCGCACCAGCACGACGTTTGGTTCGTGTTTCGCAAAAGTATCAGATCCGAACTCTACAATATGGGTCGGAGGAAAGGGCGTT
>Cm-PiFP-04_UL22_R.ab1
ATCGCTGGTCATAGACTTGCCGTGTTTGCGGGTTTAACCAAAGATTCAGTGCGCACCAGCACGACGTTTGGTTCGTGTTTCGCAAAAGTATCAGATCCGAACTCTACAATATGGGTCGGAGGAAAGGGCGTT
>Cm-PiFP-20_UL22_R.ab1
ATCGCTGGTCATAGACTTGCCGTGTTTGCGGGTTTAACCAAAGATTCAGTGCGCACCAGCACGACGTTTGGTTCGTGTTTCGCAAAAGTATCAGATCCGAACTCTACAATATGGGTCGGAGGAAAGGGCGTT
>Cm-PiFP-51_UL22_R.ab1
ATCGCTGGTCATAGACTTGCCGTGTTTGCGGGTTTAACCAAAGATTCAGTGCGCACCAGCACGACGTTTGGTTCGTGTTTCGCAAAAGTATCAGATCCGAACTCTACAATATGGGTCGGAGGAAAGGGCGTT
>Cm-PiFP-82_UL22_R.ab1
ATCGCTGGTCATAGACTTGCCGTGTTTGCGGGTTTAACCAAAGATTCAGTGCGCACCAGCACGACGTTTGGTTCGTGTTTCGCAAAAGTATCAGATCCGAACTCTACAATATGGGTCGGAGGAAAGGGCGTT
>Cm-PiFP-86_UL22_R.ab1
ATCGCTGGTCATAGACTTGCCGTGTTTGCGGGTTTAACCAAAGATTCAGTGCGCACCAGCACGACGTTTGGTTCGTGTTTCGCAAAAGTATCAGATCCGAACTCTACAATATGGGTCGGAGGAAAGGGCGTT
>Cm-PiFP-92_UL22_R.ab1
ATCGCTGGTCATAGACTTGCCGTGTTTGCGGGTTTAACCAAAGATTCAGTGCGCACCAGCACGACGTTTGGTTCGTGTTTCGCAAAAGTATCAGATCCGAACTCTACAATATGGGTCGGAGGAAAGGGCGTT
>Cm-PiT-20_UL22_R.ab1
ATCGCTGGTCATAGACTTGCCGTGTTTGCGGGTTTAACCAAAGATTCAGTGCGCACCAGCACGACGTTTGGTTCGTGTTTCGCAAAAGTATCAGATCCGAACTCTACAATATGGGTCGGAGGAAAGGGCGTT
>Cm-PiT-51_UL22_R.ab1
ATCGCTGGTCATAGACTTGCCGTGTTTGCGGGTTTAACCAAAGATTCAGTGCGCACCAGCACGACGTTTGGTTCGTGTTTCGCAAAAGTATCAGATCCGAACTCTACAATATGGGTCGGAGGAAAGGGCGTT
>Cm-PiT-82_UL22_R.ab1
ATCGCTGGTCATAGACTTGCCGTGTTTGCGGGTTTAACCAAAGATTCAGTGCGCACCAGCACGACGTTTGGTTCGTGTTTCGCAAAAGTATCAGATCCGAACTCTACAATATGGGTCGGAGGAAAGGGCGTT
>Cm-PiT-86_UL22_R.ab1
ATCGCTGGTCATAGACTTGCCGTGTTTGCGGGTTTAACCAAAGATTCAGTGCGCACCAGCACGACGTTTGGTTCGTGTTTCGCAAAAGTATCAGATCCGAACTCTACAATATGGGTCGGAGGAAAGGGCGTT

>Cm-PRT-04_UL22_R.ab1
ATCGCTGGTCATAGACTTGCCGTGTTTGCGGGTTTAACCAAAGATTCAGTGCGCACCAGCACGACGTTTGGTTCGTGTTTCGCAAAAGTATCAGATCCGAACTCTACAATATGGGTCGGAGGAAAGGGCGTT
>Cm-PRT-06_UL22_R.ab1
ATCGCTGGTCATAGACTTGCCGTGTTTGCGGGTTTAACCAAAGATTCAGTGCGCACCAGCACGACGTTTGGTTCGTGTTTCGCAAAAGTATCAGATCCGAACTCTACAATATGGGTCGGAGGAAAGGGCGTT
>Cm-TCFP-1cloaca1_UL22_R.ab1
ATCGCTGGTCATAGACTTGCCGTGTTTGCGGGTTTAACCAAAGATTCAGTGCGCACCAGCACGACGTTTGGTTCGTGTTTCGCAAAAGTATCAGATCCGAACTCTACAATATGGGTCGGAGGAAAGGGCGTT
>Cm-TCFP-1HLflipper_UL22_R.ab1
ATCGCTGGTCATAGACTTGCCGTGTTTGCGGGTTTAACCAAAGATTCAGTGCGCACCAGCACGACGTTTGGTTCGTGTTTCGCAAAAGTATCAGATCCGAACTCTACAATATGGGTCGGAGGAAAGGGCGTT
>Cm-TCFP-1Lflipper1_UL22_R.ab1
ATCGCTGGTCATAGACTTGCCGTGTTTGCGGGTTTAACCAAAGATTCAGTGCGCACCAGCACGACGTTTGGTTCGTGTTTCGCAAAAGTATCAGATCCGAACTCTACAATATGGGTCGGAGGAAAGGGCGTT
>Cm-TCFP-1RFflipper_UL22_R.ab1
ATCGCTGGTCATAGACTTGCCGTGTTTGCGGGTTTAACCAAAGATTCAGTGCGCACCAGCACGACGTTTGGTTCGTGTTTCGCAAAAGTATCAGATCCGAACTCTACAATATGGGTCGGAGGAAAGGGCGTT
>Cm-TCFP-1Rteye_UL22_R.ab1
ATCGCTGGTCATAGACTTGCCGTGTTTGCGGGTTTAACCAAAGATTCAGTGCGCACCAGCACGACGTTTGGTTCGTGTTTCGCAAAAGTATCAGATCCGAACTCTACAATATGGGTCGGAGGAAAGGGCGTT
>Cm-TCFP-2_UL22_R.ab1
ATCGCTGGTCATAGACTTGCCGTGTTTGCGGGTTTAACCAAAGATTCAGTGCGCACCAGCACGACGTTTGGTTCGTGTTTCGCAAAAGTATCAGATCCGAACTCTACAATATGGGTCGGAGGAAAGGGCGTT
>Cm-ToT-09_UL22_R.ab1
ATCGCTGGTCATAGACTTGCCGTGTTTGCGGGTTTAACCAAAGATTCAGTGCGCACCAGCACGACGTTTGGTTCGTGTTTCGCAAAAGTATCAGATCCGAACTCTACAATATGGGTCGGAGGAAAGGGCGTT
>Cm-ToT-35_UL22_R.ab1
ATCGCTGGTCATAGACTTGCCGTGTTTGCGGGTTTAACCAAAGATTCAGTGCGCACCAGCACGACGTTTGGTTCGTGTTTCGCAAAAGTATCAGATCCGAACTCTACAATATGGGTCGGAGGAAAGGGCGTT
>Cm-ToT-36_UL22_R.ab1
ATCGCTGGTCATAGACTTGCCGTGTTTGCGGGTTTAACCAAAGATTCAGTGCGCACCAGCACGACGTTTGGTTCGTGTTTCGCAAAAGTATCAGATCCGAACTCTACAATATGGGTCGGAGGAAAGGGCGTT
>Cm-ToT-37_UL22_R.ab1
ATCGCTGGTCATAGACTTGCCGTGTTTGCGGGTTTAACCAAAGATTCAGTGCGCACCAGCACGACGTTTGGTTCGTGTTTCGCAAAAGTATCAGATCCGAACTCTACAATATGGGTCGGAGGAAAGGGCGTT
>Cm-ToT-38_UL22_R.ab1
ATCGCTGGTCATAGACTTGCCGTGTTTGCGGGTTTAACCAAAGATTCAGTGCGCACCAGCACGACGTTTGGTTCGTGTTTCGCAAAAGTATCAGATCCGAACTCTACAATATGGGTCGGAGGAAAGGGCGTT
>Cm-ToT-39_UL22_R.ab1
ATCGCTGGTCATAGACTTGCCGTGTTTGCGGGTTTAACCAAAGATTCAGTGCGCACCAGCACGACGTTTGGTTCGTGTTTCGCAAAAGTATCAGATCCGAACTCTACAATATGGGTCGGAGGAAAGGGCGTT
>Cm-ToT-40_UL22_R.ab1
ATCGCTGGTCATAGACTTGCCGTGTTTGCGGGTTTAACCAAAGATTCAGTGCGCACCAGCACGACGTTTGGTTCGTGTTTCGCAAAAGTATCAGATCCGAACTCTACAATATGGGTCGGAGGAAAGGGCGTT
>Ei-KuT-03_UL22_R.ab1
ATCGCTGGTCATAGACTTGCCGTGTTTGCGGGTTTAACCAAAGATTCAGTGCGCACCAGCACGACGTTTGGTTCGTGTTTCGCAAAAGTATCAGATCCGAACTCTACAATATGGGTCGGAGGAAAGGGCGTT
>Ei-KuT-14_UL22_R.ab1
ATCGCTGGTCATAGACTTGCCGTGTTTGCGGGTTTAACCAAAGATTCAGTGCGCACCAGCACGACGTTTGGTTCGTGTTTCGCAAAAGTATCAGATCCGAACTCTACAATATGGGTCGGAGGAAAGGGCGTT
>Ei-KuT-16_UL22_R.ab1
ATCGCTGGTCATAGACTTGCCGTGTTTGCGGGTTTAACCAAAGATTCAGTGCGCACCAGCACGACGTTTGGTTCGTGTTTCGCAAAAGTATCAGATCCGAACTCTACAATATGGGTCGGAGGAAAGGGCGTT
>Ei-KuT-19_UL22_R.ab1
ATCGCTGGTCATAGACTTGCCGTGTTTGCGGGTTTAACCAAAGATTCAGTGCGCACCAGCACGACGTTTGGTTCGTGTTTCGCAAAAGTATCAGATCCGAACTCTACAATATGGGTCGGAGGAAAGGGCGTT
>Ei-PiT-46_UL22_R.ab1
ATCGCTGGTCATAGACTTGCCGTGTTTGCGGGTTTAACCAAAGATTCAGTGCGCACCAGCACGACGTTTGGTTCGTGTTTCGCAAAAGTATCAGATCCGAACTCTACAATATGGGTCGGAGGAAAGGGCGTT
>Ei-PiT-71_UL22_R.ab1
ATCGCTGGTCATAGACTTGCCGTGTTTGCGGGTTTAACCAAAGATTCAGTGCGCACCAGCACGACGTTTGGTTCGTGTTTCGCAAAAGTATCAGATCCGAACTCTACAATATGGGTCGGAGGAAAGGGCGTT

<UL22_T&C_haplotype2>

>Cm-TCFP-15_UL22_R.ab1
ATCGCTGGTCATAGACTTGCCGTGTTTGCGGGTTTAACCAAGGATTCAGTGCGCACCAGCACGACGTTTGGTTCGTGTTTCGCAAAAGTTTCAGATCCGAACTCTACAATATGGGTCGGAGGAAAGGGCGTT
>Cm-PoT-02_UL22_R.ab1
ATCGCTGGTCATAGACTTGCCGTGTTTGCGGGTTTAACCAAGGATTCAGTGCGCACCAGCACGACGTTTGGTTCGTGTTTCGCAAAAGTTTCAGATCCGAACTCTACAATATGGGTCGGAGGAAAGGGCGTT
>Cm-TCFP-1Lflipper2_UL22_R.ab1
ATCGCTGGTCATAGACTTGCCGTGTTTGCGGGTTTAACCAAGGATTCAGTGCGCACCAGCACGACGTTTGGTTCGTGTTTCGCAAAAGTTTCAGATCCGAACTCTACAATATGGGTCGGAGGAAAGGGCGTT
>Cm-TCFP-3_UL22_R.ab1
ATCGCTGGTCATAGACTTGCCGTGTTTGCGGGTTTAACCAAGGATTCAGTGCGCACCAGCACGACGTTTGGTTCGTGTTTCGCAAAAGTTTCAGATCCGAACTCTACAATATGGGTCGGAGGAAAGGGCGTT
>Cm-TCFP-4_UL22_R.ab1
ATCGCTGGTCATAGACTTGCCGTGTTTGCGGGTTTAACCAAGGATTCAGTGCGCACCAGCACGACGTTTGGTTCGTGTTTCGCAAAAGTTTCAGATCCGAACTCTACAATATGGGTCGGAGGAAAGGGCGTT
>Cm-TCFP-5_UL22_R.ab1
ATCGCTGGTCATAGACTTGCCGTGTTTGCGGGTTTAACCAAGGATTCAGTGCGCACCAGCACGACGTTTGGTTCGTGTTTCGCAAAAGTTTCAGATCCGAACTCTACAATATGGGTCGGAGGAAAGGGCGTT
>Cm-TCFP-6_UL22_R.ab1
ATCGCTGGTCATAGACTTGCCGTGTTTGCGGGTTTAACCAAGGATTCAGTGCGCACCAGCACGACGTTTGGTTCGTGTTTCGCAAAAGTTTCAGATCCGAACTCTACAATATGGGTCGGAGGAAAGGGCGTT
>Cm-TCFP-8_UL22_R.ab1
ATCGCTGGTCATAGACTTGCCGTGTTTGCGGGTTTAACCAAGGATTCAGTGCGCACCAGCACGACGTTTGGTTCGTGTTTCGCAAAAGTTTCAGATCCGAACTCTACAATATGGGTCGGAGGAAAGGGCGTT
>Cm-TCFP-9_UL22_R.ab1
ATCGCTGGTCATAGACTTGCCGTGTTTGCGGGTTTAACCAAGGATTCAGTGCGCACCAGCACGACGTTTGGTTCGTGTTTCGCAAAAGTTTCAGATCCGAACTCTACAATATGGGTCGGAGGAAAGGGCGTT
>Cm-TCFP-11_UL22_R.ab1
ATCGCTGGTCATAGACTTGCCGTGTTTGCGGGTTTAACCAAGGATTCAGTGCGCACCAGCACGACGTTTGGTTCGTGTTTCGCAAAAGTTTCAGATCCGAACTCTACAATATGGGTCGGAGGAAAGGGCGTT
>Cm-TCFP-12_UL22_R.ab1
ATCGCTGGTCATAGACTTGCCGTGTTTGCGGGTTTAACCAAGGATTCAGTGCGCACCAGCACGACGTTTGGTTCGTGTTTCGCAAAAGTTTCAGATCCGAACTCTACAATATGGGTCGGAGGAAAGGGCGTT
>Cm-TCFP-13_UL22_R.ab1
ATCGCTGGTCATAGACTTGCCGTGTTTGCGGGTTTAACCAAGGATTCAGTGCGCACCAGCACGACGTTTGGTTCGTGTTTCGCAAAAGTTTCAGATCCGAACTCTACAATATGGGTCGGAGGAAAGGGCGTT
>Cm-TCFP-14_UL22_R.ab1
ATCGCTGGTCATAGACTTGCCGTGTTTGCGGGTTTAACCAAGGATTCAGTGCGCACCAGCACGACGTTTGGTTCGTGTTTCGCAAAAGTTTCAGATCCGAACTCTACAATATGGGTCGGAGGAAAGGGCGTT

<UL27_haplotype1>

>Cc-CyT-012_UL27_C.ab1
CCTCCGGATGGTCGCTGGCCAAAGAAAAACTATCTAACGTCAAAGACAACTTTACGGACGCTAAAAATAAAATGATGAAATCTTTACGCCACCGCGGAAACGGATACGCTCCCGTTTCGACGAGCATGGCCAGTATGTATCTAG
>Lo-OsT-01_UL27_C.ab1
CCTCCGGATGGTCGCTGGCCAAAGAAAAACTATCTAACGTCAAAGACAACTTTACGGACGCTAAAAATAAAATGATGAAATCTTTACGCCACCGCGGAAACGGATACGCTCCCGTTTCGACGAGCATGGCCAGTATGTATCTAG
>Lo-GhT-38A_UL27_C.ab1
CCTCCGGATGGTCGCTGGCCAAAGAAAAACTATCTAACGTCAAAGACAACTTTACGGACGCTAAAAATAAAATGATGAAATCTTTACGCCACCGCGGAAACGGATACGCTCCCGTTTCGACGAGCATGGCCAGTATGTATCTAG
>Ei-PiT-85_UL27_C.ab1
CCTCCGGATGGTCGCTGGCCAAAGAAAAACTATCTAACGTCAAAGACAACTTTACGGACGCTAAAAATAAAATGATGAAATCTTTACGCCACCGCGGAAACGGATACGCTCCCGTTTCGACGAGCATGGCCAGTATGTATCTAG
>Ei-PiT-71_UL27_C.ab1
CCTCCGGATGGTCGCTGGCCAAAGAAAAACTATCTAACGTCAAAGACAACTTTACGGACGCTAAAAATAAAATGATGAAATCTTTACGCCACCGCGGAAACGGATACGCTCCCGTTTCGACGAGCATGGCCAGTATGTATCTAG
>Ei-PiT-46_UL27_R.ab1
CCTCCGGATGGTCGCTGGCCAAAGAAAAACTATCTAACGTCAAAGACAACTTTACGGACGCTAAAAATAAAATGATGAAATCTTTACGCCACCGCGGAAACGGATACGCTCCCGTTTCGACGAGCATGGCCAGTATGTATCTAG
>Ei-PiT-25_UL27_C.ab1
CCTCCGGATGGTCGCTGGCCAAAGAAAAACTATCTAACGTCAAAGACAACTTTACGGACGCTAAAAATAAAATGATGAAATCTTTACGCCACCGCGGAAACGGATACGCTCCCGTTTCGACGAGCATGGCCAGTATGTATCTAG
>Dc-PoT-1A_UL27_C.ab1
CCTCCGGATGGTCGCTGGCCAAAGAAAAACTATCTAACGTCAAAGACAACTTTACGGACGCTAAAAATAAAATGATGAAATCTTTACGCCACCGCGGAAACGGATACGCTCCCGTTTCGACGAGCATGGCCAGTATGTATCTAG
>Dc-GhT-23_UL27_C.ab1
CCTCCGGATGGTCGCTGGCCAAAGAAAAACTATCTAACGTCAAAGACAACTTTACGGACGCTAAAAATAAAATGATGAAATCTTTACGCCACCGCGGAAACGGATACGCTCCCGTTTCGACGAGCATGGCCAGTATGTATCTAG
>Dc-GhT-17_UL27_C.ab1
CCTCCGGATGGTCGCTGGCCAAAGAAAAACTATCTAACGTCAAAGACAACTTTACGGACGCTAAAAATAAAATGATGAAATCTTTACGCCACCGCGGAAACGGATACGCTCCCGTTTCGACGAGCATGGCCAGTATGTATCTAG
>Cm-TCFP-14_UL27_C.ab1
CCTCCGGATGGTCGCTGGCCAAAGAAAAACTATCTAACGTCAAAGACAACTTTACGGACGCTAAAAATAAAATGATGAAATCTTTACGCCACCGCGGAAACGGATACGCTCCCGTTTCGACGAGCATGGCCAGTATGTATCTAG
>Cm-TCFP-13_UL27_R.ab1
CCTCCGGATGGTCGCTGGCCAAAGAAAAACTATCTAACGTCAAAGACAACTTTACGGACGCTAAAAATAAAATGATGAAATCTTTACGCCACCGCGGAAACGGATACGCTCCCGTTTCGACGAGCATGGCCAGTATGTATCTAG
>Cm-TCFP-7_UL27_C.ab1
CCTCCGGATGGTCGCTGGCCAAAGAAAAACTATCTAACGTCAAAGACAACTTTACGGACGCTAAAAATAAAATGATGAAATCTTTACGCCACCGCGGAAACGGATACGCTCCCGTTTCGACGAGCATGGCCAGTATGTATCTAG
>Cm-PiFP-92_UL27_R.ab1
CCTCCGGATGGTCGCTGGCCAAAGAAAAACTATCTAACGTCAAAGACAACTTTACGGACGCTAAAAATAAAATGATGAAATCTTTACGCCACCGCGGAAACGGATACGCTCCCGTTTCGACGAGCATGGCCAGTATGTATCTAG
>Cm-PiFP-82_UL27_R.ab1
CCTCCGGATGGTCGCTGGCCAAAGAAAAACTATCTAACGTCAAAGACAACTTTACGGACGCTAAAAATAAAATGATGAAATCTTTACGCCACCGCGGAAACGGATACGCTCCCGTTTCGACGAGCATGGCCAGTATGTATCTAG
>Cm-PiFP-51_UL27_F.ab1
CCTCCGGATGGTCGCTGGCCAAAGAAAAACTATCTAACGTCAAAGACAACTTTACGGACGCTAAAAATAAAATGATGAAATCTTTACGCCACCGCGGAAACGGATACGCTCCCGTTTCGACGAGCATGGCCAGTATGTATCTAG
>Cm-PiFP-20_UL27_C.ab1
CCTCCGGATGGTCGCTGGCCAAAGAAAAACTATCTAACGTCAAAGACAACTTTACGGACGCTAAAAATAAAATGATGAAATCTTTACGCCACCGCGGAAACGGATACGCTCCCGTTTCGACGAGCATGGCCAGTATGTATCTAG
>Cm-PiFP-04_UL27_C.ab1
CCTCCGGATGGTCGCTGGCCAAAGAAAAACTATCTAACGTCAAAGACAACTTTACGGACGCTAAAAATAAAATGATGAAATCTTTACGCCACCGCGGAAACGGATACGCTCCCGTTTCGACGAGCATGGCCAGTATGTATCTAG
>Cm-HaT-14_UL27_C.ab1
CCTCCGGATGGTCGCTGGCCAAAGAAAAACTATCTAACGTCAAAGACAACTTTACGGACGCTAAAAATAAAATGATGAAATCTTTACGCCACCGCGGAAACGGATACGCTCCCGTTTCGACGAGCATGGCCAGTATGTATCTAG
>Cm-HaT-13_UL27_F.ab1
CCTCCGGATGGTCGCTGGCCAAAGAAAAACTATCTAACGTCAAAGACAACTTTACGGACGCTAAAAATAAAATGATGAAATCTTTACGCCACCGCGGAAACGGATACGCTCCCGTTTCGACGAGCATGGCCAGTATGTATCTAG
>Cm-HaT-12_UL27_F.ab1
CCTCCGGATGGTCGCTGGCCAAAGAAAAACTATCTAACGTCAAAGACAACTTTACGGACGCTAAAAATAAAATGATGAAATCTTTACGCCACCGCGGAAACGGATACGCTCCCGTTTCGACGAGCATGGCCAGTATGTATCTAG
>Cm-HaT-11_UL27_C.ab1
CCTCCGGATGGTCGCTGGCCAAAGAAAAACTATCTAACGTCAAAGACAACTTTACGGACGCTAAAAATAAAATGATGAAATCTTTACGCCACCGCGGAAACGGATACGCTCCCGTTTCGACGAGCATGGCCAGTATGTATCTAG
>Cm-HaT-10_UL27_R.ab1
CCTCCGGATGGTCGCTGGCCAAAGAAAAACTATCTAACGTCAAAGACAACTTTACGGACGCTAAAAATAAAATGATGAAATCTTTACGCCACCGCGGAAACGGATACGCTCCCGTTTCGACGAGCATGGCCAGTATGTATCTAG
>Cm-HaT-8_UL27_C.ab1
CCTCCGGATGGTCGCTGGCCAAAGAAAAACTATCTAACGTCAAAGACAACTTTACGGACGCTAAAAATAAAATGATGAAATCTTTACGCCACCGCGGAAACGGATACGCTCCCGTTTCGACGAGCATGGCCAGTATGTATCTAG

>Cm-HaT-5_UL27_C.ab1
CCTCCGGATGGTCGCTGGCCAAAGAAAAACTATCTAACGTCAAAGACAACTTTACGGACGCTAAAAATAAAATGATGAAATCTTTACGCCACCGCGGAAACGGATACGCTCCCGTTTCGACGAGCATGGCCAGTATGTATCTAG
>Cm-HaT-4_UL27_C.ab1
CCTCCGGATGGTCGCTGGCCAAAGAAAAACTATCTAACGTCAAAGACAACTTTACGGACGCTAAAAATAAAATGATGAAATCTTTACGCCACCGCGGAAACGGATACGCTCCCGTTTCGACGAGCATGGCCAGTATGTATCTAG
>Cm-HaT-3_UL27_C.ab1
CCTCCGGATGGTCGCTGGCCAAAGAAAAACTATCTAACGTCAAAGACAACTTTACGGACGCTAAAAATAAAATGATGAAATCTTTACGCCACCGCGGAAACGGATACGCTCCCGTTTCGACGAGCATGGCCAGTATGTATCTAG
>Cm-HaFP-15NECK_UL27_R.ab1
CCTCCGGATGGTCGCTGGCCAAAGAAAAACTATCTAACGTCAAAGACAACTTTACGGACGCTAAAAATAAAATGATGAAATCTTTACGCCACCGCGGAAACGGATACGCTCCCGTTTCGACGAGCATGGCCAGTATGTATCTAG
>Cm-HaFP-14TAIL_UL27_C.ab1
CCTCCGGATGGTCGCTGGCCAAAGAAAAACTATCTAACGTCAAAGACAACTTTACGGACGCTAAAAATAAAATGATGAAATCTTTACGCCACCGCGGAAACGGATACGCTCCCGTTTCGACGAGCATGGCCAGTATGTATCTAG
>Cm-HaFP-14RHF_UL27_F.ab1
CCTCCGGATGGTCGCTGGCCAAAGAAAAACTATCTAACGTCAAAGACAACTTTACGGACGCTAAAAATAAAATGATGAAATCTTTACGCCACCGCGGAAACGGATACGCTCCCGTTTCGACGAGCATGGCCAGTATGTATCTAG
>Cm-HaFP-14NECK_UL27_C.ab1
CCTCCGGATGGTCGCTGGCCAAAGAAAAACTATCTAACGTCAAAGACAACTTTACGGACGCTAAAAATAAAATGATGAAATCTTTACGCCACCGCGGAAACGGATACGCTCCCGTTTCGACGAGCATGGCCAGTATGTATCTAG
>Cm-HaFP-12LHF_UL27_C.ab1
CCTCCGGATGGTCGCTGGCCAAAGAAAAACTATCTAACGTCAAAGACAACTTTACGGACGCTAAAAATAAAATGATGAAATCTTTACGCCACCGCGGAAACGGATACGCTCCCGTTTCGACGAGCATGGCCAGTATGTATCTAG
>Cm-HaFP-12LFF_UL27_R.ab1
CCTCCGGATGGTCGCTGGCCAAAGAAAAACTATCTAACGTCAAAGACAACTTTACGGACGCTAAAAATAAAATGATGAAATCTTTACGCCACCGCGGAAACGGATACGCTCCCGTTTCGACGAGCATGGCCAGTATGTATCTAG
>Cm-HaFP-11LHF_UL27_F.ab1
CCTCCGGATGGTCGCTGGCCAAAGAAAAACTATCTAACGTCAAAGACAACTTTACGGACGCTAAAAATAAAATGATGAAATCTTTACGCCACCGCGGAAACGGATACGCTCCCGTTTCGACGAGCATGGCCAGTATGTATCTAG
>Cm-HaFP-10RFF_UL27_C.ab1
CCTCCGGATGGTCGCTGGCCAAAGAAAAACTATCTAACGTCAAAGACAACTTTACGGACGCTAAAAATAAAATGATGAAATCTTTACGCCACCGCGGAAACGGATACGCTCCCGTTTCGACGAGCATGGCCAGTATGTATCTAG
>Cm-HaFP-10LFF_UL27_R.ab1
CCTCCGGATGGTCGCTGGCCAAAGAAAAACTATCTAACGTCAAAGACAACTTTACGGACGCTAAAAATAAAATGATGAAATCTTTACGCCACCGCGGAAACGGATACGCTCCCGTTTCGACGAGCATGGCCAGTATGTATCTAG
>Cm-HaFP-9RFF_UL27_F.ab1
CCTCCGGATGGTCGCTGGCCAAAGAAAAACTATCTAACGTCAAAGACAACTTTACGGACGCTAAAAATAAAATGATGAAATCTTTACGCCACCGCGGAAACGGATACGCTCCCGTTTCGACGAGCATGGCCAGTATGTATCTAG
>Cm-HaFP-5RFF_UL27_C.ab1
CCTCCGGATGGTCGCTGGCCAAAGAAAAACTATCTAACGTCAAAGACAACTTTACGGACGCTAAAAATAAAATGATGAAATCTTTACGCCACCGCGGAAACGGATACGCTCCCGTTTCGACGAGCATGGCCAGTATGTATCTAG
>Cm-HaFP-5LFF_UL27_C.ab1
CCTCCGGATGGTCGCTGGCCAAAGAAAAACTATCTAACGTCAAAGACAACTTTACGGACGCTAAAAATAAAATGATGAAATCTTTACGCCACCGCGGAAACGGATACGCTCCCGTTTCGACGAGCATGGCCAGTATGTATCTAG
>Cm-HaFP-3NECK_UL27_C.ab1
CCTCCGGATGGTCGCTGGCCAAAGAAAAACTATCTAACGTCAAAGACAACTTTACGGACGCTAAAAATAAAATGATGAAATCTTTACGCCACCGCGGAAACGGATACGCTCCCGTTTCGACGAGCATGGCCAGTATGTATCTAG
>Cm-HaFP-3LHF_UL27_C.ab1
CCTCCGGATGGTCGCTGGCCAAAGAAAAACTATCTAACGTCAAAGACAACTTTACGGACGCTAAAAATAAAATGATGAAATCTTTACGCCACCGCGGAAACGGATACGCTCCCGTTTCGACGAGCATGGCCAGTATGTATCTAG
>Cm-HaFP-2LHF_UL27_C.ab1
CCTCCGGATGGTCGCTGGCCAAAGAAAAACTATCTAACGTCAAAGACAACTTTACGGACGCTAAAAATAAAATGATGAAATCTTTACGCCACCGCGGAAACGGATACGCTCCCGTTTCGACGAGCATGGCCAGTATGTATCTAG
>Cm-HaFP-2LFF_UL27_C.ab1
CCTCCGGATGGTCGCTGGCCAAAGAAAAACTATCTAACGTCAAAGACAACTTTACGGACGCTAAAAATAAAATGATGAAATCTTTACGCCACCGCGGAAACGGATACGCTCCCGTTTCGACGAGCATGGCCAGTATGTATCTAG
>Cm-HaFP-1RMOUTH_UL27_C.ab1
CCTCCGGATGGTCGCTGGCCAAAGAAAAACTATCTAACGTCAAAGACAACTTTACGGACGCTAAAAATAAAATGATGAAATCTTTACGCCACCGCGGAAACGGATACGCTCCCGTTTCGACGAGCATGGCCAGTATGTATCTAG
>Cm-HaFP-1LHF_UL27_C.ab1
CCTCCGGATGGTCGCTGGCCAAAGAAAAACTATCTAACGTCAAAGACAACTTTACGGACGCTAAAAATAAAATGATGAAATCTTTACGCCACCGCGGAAACGGATACGCTCCCGTTTCGACGAGCATGGCCAGTATGTATCTAG
>Cm-CyT-070_UL27_F.ab1
CCTCCGGATGGTCGCTGGCCAAAGAAAAACTATCTAACGTCAAAGACAACTTTACGGACGCTAAAAATAAAATGATGAAATCTTTACGCCACCGCGGAAACGGATACGCTCCCGTTTCGACGAGCATGGCCAGTATGTATCTAG
>Cm-CyT-015_UL27_F.ab1
CCTCCGGATGGTCGCTGGCCAAAGAAAAACTATCTAACGTCAAAGACAACTTTACGGACGCTAAAAATAAAATGATGAAATCTTTACGCCACCGCGGAAACGGATACGCTCCCGTTTCGACGAGCATGGCCAGTATGTATCTAG
>Cma-SDT-02_UL27_C.ab1
CCTCCGGATGGTCGCTGGCCAAAGAAAAACTATCTAACGTCAAAGACAACTTTACGGACGCTAAAAATAAAATGATGAAATCTTTACGCCACCGCGGAAACGGATACGCTCCCGTTTCGACGAGCATGGCCAGTATGTATCTAG
>Cc-DkT-01_UL27_C.ab1
CCTCCGGATGGTCGCTGGCCAAAGAAAAACTATCTAACGTCAAAGACAACTTTACGGACGCTAAAAATAAAATGATGAAATCTTTACGCCACCGCGGAAACGGATACGCTCCCGTTTCGACGAGCATGGCCAGTATGTATCTAG
>Cc-CyT-275_UL27_C.ab1
CCTCCGGATGGTCGCTGGCCAAAGAAAAACTATCTAACGTCAAAGACAACTTTACGGACGCTAAAAATAAAATGATGAAATCTTTACGCCACCGCGGAAACGGATACGCTCCCGTTTCGACGAGCATGGCCAGTATGTATCTAG

<UL27_haplotype2>

>Cm-PiT-92_UL27_C.ab1
CCTCCGGATGGTCGCTGGCCAAAGAAAAACTATCTAACGTCAAAGACAACTTTACGGACGCTAAAAATAAAATGATGAAATCTTTACGCCACCGCGGAAACGGATACGCTCCCGTTTCGACGAGCACGGCCAGTATGTATCTAG
>Cm-HaT-6_UL27_C.ab1
CCTCCGGATGGTCGCTGGCCAAAGAAAAACTATCTAACGTCAAAGACAACTTTACGGACGCTAAAAATAAAATGATGAAATCTTTACGCCACCGCGGAAACGGATACGCTCCCGTTTCGACGAGCACGGCCAGTATGTATCTAG
>Cm-HaT-2_UL27_C.ab1
CCTCCGGATGGTCGCTGGCCAAAGAAAAACTATCTAACGTCAAAGACAACTTTACGGACGCTAAAAATAAAATGATGAAATCTTTACGCCACCGCGGAAACGGATACGCTCCCGTTTCGACGAGCACGGCCAGTATGTATCTAG
>Cm-HaFP-8RHF_UL27_R.ab1
CCTCCGGATGGTCGCTGGCCAAAGAAAAACTATCTAACGTCAAAGACAACTTTACGGACGCTAAAAATAAAATGATGAAATCTTTACGCCACCGCGGAAACGGATACGCTCCCGTTTCGACGAGCACGGCCAGTATGTATCTAG
>Cm-HaFP-8MAXILLA_UL27_R.ab1
CCTCCGGATGGTCGCTGGCCAAAGAAAAACTATCTAACGTCAAAGACAACTTTACGGACGCTAAAAATAAAATGATGAAATCTTTACGCCACCGCGGAAACGGATACGCTCCCGTTTCGACGAGCACGGCCAGTATGTATCTAG
>Cm-HaFP-7LHF_UL27_R.ab1
CCTCCGGATGGTCGCTGGCCAAAGAAAAACTATCTAACGTCAAAGACAACTTTACGGACGCTAAAAATAAAATGATGAAATCTTTACGCCACCGCGGAAACGGATACGCTCCCGTTTCGACGAGCACGGCCAGTATGTATCTAG
>Cc-PoT-01_UL27_C.ab1
CCTCCGGATGGTCGCTGGCCAAAGAAAAACTATCTAACGTCAAAGACAACTTTACGGACGCTAAAAATAAAATGATGAAATCTTTACGCCACCGCGGAAACGGATACGCTCCCGTTTCGACGAGCACGGCCAGTATGTATCTAG

<UL27_haplotype3>

>Cma-SDT-01_UL27_C.ab1
CCTCCGGATGGTCGCTGGCCAAGGAAAAACTATCTAACGTCAAAGACAACTTTACGGACGCTAAAAATAAAATGATGAAATCTTTACGCCACCGCGGAAACGGATACGCTCCCGTTTCGACGAGCATGGCCAGTATGTATCTAG
>Dc-OsT-01_UL27_C.ab1
CCTCCGGATGGTCGCTGGCCAAGGAAAAACTATCTAACGTCAAAGACAACTTTACGGACGCTAAAAATAAAATGATGAAATCTTTACGCCACCGCGGAAACGGATACGCTCCCGTTTCGACGAGCATGGCCAGTATGTATCTAG
>Cm-TCFP-15_UL27_C.ab1
CCTCCGGATGGTCGCTGGCCAAGGAAAAACTATCTAACGTCAAAGACAACTTTACGGACGCTAAAAATAAAATGATGAAATCTTTACGCCACCGCGGAAACGGATACGCTCCCGTTTCGACGAGCATGGCCAGTATGTATCTAG
>Cm-TCFP-12_UL27_R.ab1
CCTCCGGATGGTCGCTGGCCAAGGAAAAACTATCTAACGTCAAAGACAACTTTACGGACGCTAAAAATAAAATGATGAAATCTTTACGCCACCGCGGAAACGGATACGCTCCCGTTTCGACGAGCATGGCCAGTATGTATCTAG
>Cm-TCFP-10_UL27_C.ab1
CCTCCGGATGGTCGCTGGCCAAGGAAAAACTATCTAACGTCAAAGACAACTTTACGGACGCTAAAAATAAAATGATGAAATCTTTACGCCACCGCGGAAACGGATACGCTCCCGTTTCGACGAGCATGGCCAGTATGTATCTAG
>Cm-TCFP-8_UL27_F.ab1
CCTCCGGATGGTCGCTGGCCAAGGAAAAACTATCTAACGTCAAAGACAACTTTACGGACGCTAAAAATAAAATGATGAAATCTTTACGCCACCGCGGAAACGGATACGCTCCCGTTTCGACGAGCATGGCCAGTATGTATCTAG
>Cm-TCFP-5_UL27_R.ab1
CCTCCGGATGGTCGCTGGCCAAGGAAAAACTATCTAACGTCAAAGACAACTTTACGGACGCTAAAAATAAAATGATGAAATCTTTACGCCACCGCGGAAACGGATACGCTCCCGTTTCGACGAGCATGGCCAGTATGTATCTAG
>Cm-TCFP-4_UL27_R.ab1
CCTCCGGATGGTCGCTGGCCAAGGAAAAACTATCTAACGTCAAAGACAACTTTACGGACGCTAAAAATAAAATGATGAAATCTTTACGCCACCGCGGAAACGGATACGCTCCCGTTTCGACGAGCATGGCCAGTATGTATCTAG
>Cm-TCFP-3_UL27_C.ab1
CCTCCGGATGGTCGCTGGCCAAGGAAAAACTATCTAACGTCAAAGACAACTTTACGGACGCTAAAAATAAAATGATGAAATCTTTACGCCACCGCGGAAACGGATACGCTCCCGTTTCGACGAGCATGGCCAGTATGTATCTAG
>Cm-TCFP-2_UL27_C.ab1
CCTCCGGATGGTCGCTGGCCAAGGAAAAACTATCTAACGTCAAAGACAACTTTACGGACGCTAAAAATAAAATGATGAAATCTTTACGCCACCGCGGAAACGGATACGCTCCCGTTTCGACGAGCATGGCCAGTATGTATCTAG
>Cm-TCFP-1RFflipper_UL27_C.ab1
CCTCCGGATGGTCGCTGGCCAAGGAAAAACTATCTAACGTCAAAGACAACTTTACGGACGCTAAAAATAAAATGATGAAATCTTTACGCCACCGCGGAAACGGATACGCTCCCGTTTCGACGAGCATGGCCAGTATGTATCTAG
>Cm-TCFP-1HLFlipper_UL27_F.ab1
CCTCCGGATGGTCGCTGGCCAAGGAAAAACTATCTAACGTCAAAGACAACTTTACGGACGCTAAAAATAAAATGATGAAATCTTTACGCCACCGCGGAAACGGATACGCTCCCGTTTCGACGAGCATGGCCAGTATGTATCTAG
>Cm-TCFP-1Carapace_UL27_F.ab1
CCTCCGGATGGTCGCTGGCCAAGGAAAAACTATCTAACGTCAAAGACAACTTTACGGACGCTAAAAATAAAATGATGAAATCTTTACGCCACCGCGGAAACGGATACGCTCCCGTTTCGACGAGCATGGCCAGTATGTATCTAG
>Cm-PoT-01_UL27_C.ab1
CCTCCGGATGGTCGCTGGCCAAGGAAAAACTATCTAACGTCAAAGACAACTTTACGGACGCTAAAAATAAAATGATGAAATCTTTACGCCACCGCGGAAACGGATACGCTCCCGTTTCGACGAGCATGGCCAGTATGTATCTAG
>Cm-PiT-80_UL27_C.ab1
CCTCCGGATGGTCGCTGGCCAAGGAAAAACTATCTAACGTCAAAGACAACTTTACGGACGCTAAAAATAAAATGATGAAATCTTTACGCCACCGCGGAAACGGATACGCTCCCGTTTCGACGAGCATGGCCAGTATGTATCTAG
>Cm-PiT-51_UL27_C.ab1
CCTCCGGATGGTCGCTGGCCAAGGAAAAACTATCTAACGTCAAAGACAACTTTACGGACGCTAAAAATAAAATGATGAAATCTTTACGCCACCGCGGAAACGGATACGCTCCCGTTTCGACGAGCATGGCCAGTATGTATCTAG
>Cm-PiT-04_UL27_F.ab1
CCTCCGGATGGTCGCTGGCCAAGGAAAAACTATCTAACGTCAAAGACAACTTTACGGACGCTAAAAATAAAATGATGAAATCTTTACGCCACCGCGGAAACGGATACGCTCCCGTTTCGACGAGCATGGCCAGTATGTATCTAG
>Cm-PiFP-80_UL27_C.ab1
CCTCCGGATGGTCGCTGGCCAAGGAAAAACTATCTAACGTCAAAGACAACTTTACGGACGCTAAAAATAAAATGATGAAATCTTTACGCCACCGCGGAAACGGATACGCTCCCGTTTCGACGAGCATGGCCAGTATGTATCTAG
