# Supplementary figures and images for: Global distribution of Chelonid fibropapilloma-associated herpesvirus among clinically healthy sea turtles
Source: BMC Evol Biol. 2014 Oct 25;14:206. doi: 10.1186/s12862-014-0206-z (PMC4219010; doi:10.1186/s12862-014-0206-z)

- UL18
- 0
  - 1
  - 2
  - 5
  - 6
  - 8
  - 16
  - 17
  - 90
  - 993

A

# UL18 global

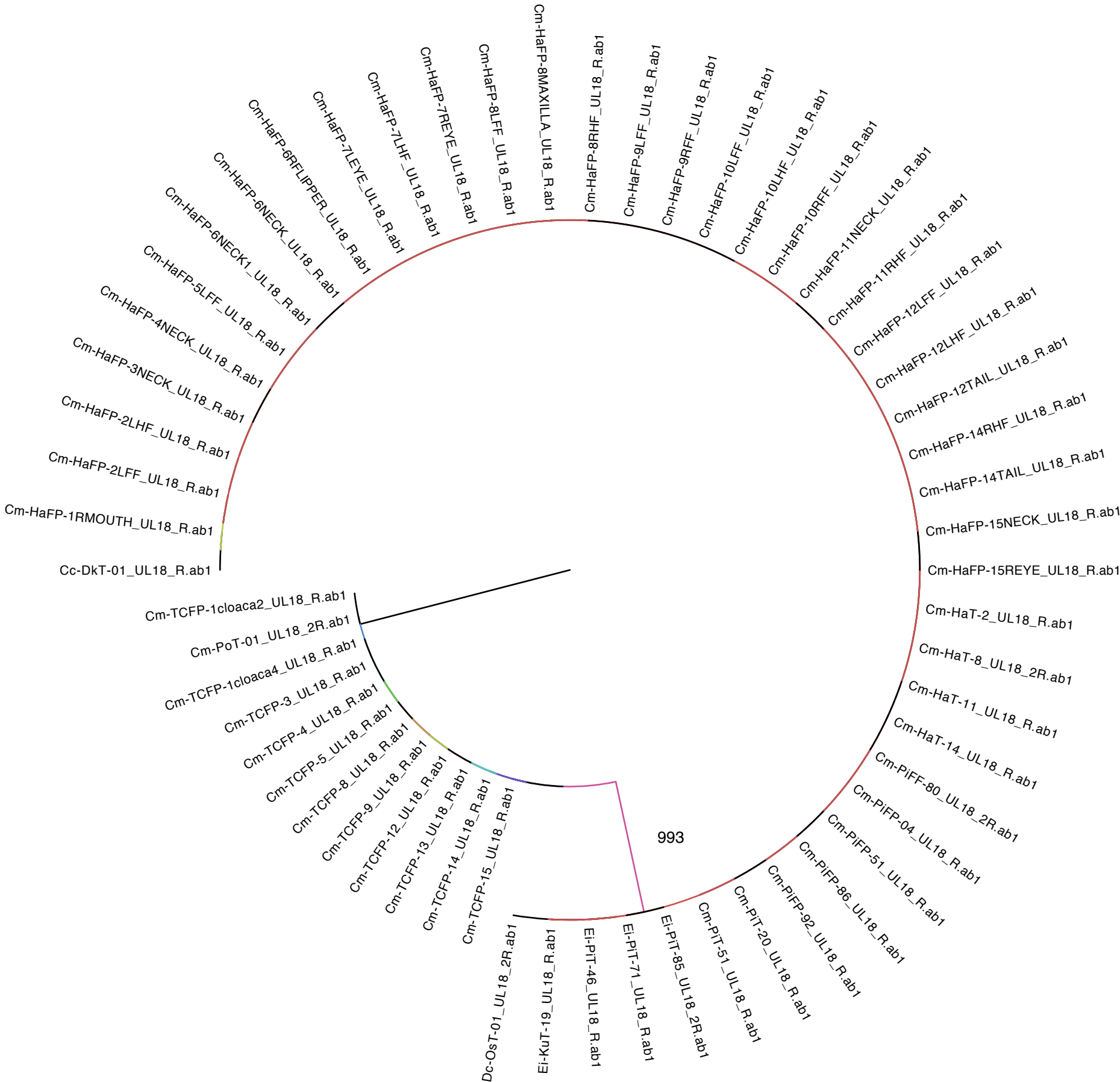

UL22

- 0
- 1
- 2
- 3
- 4
- 9
- 13
- 71
- 864

B

# UL22 global

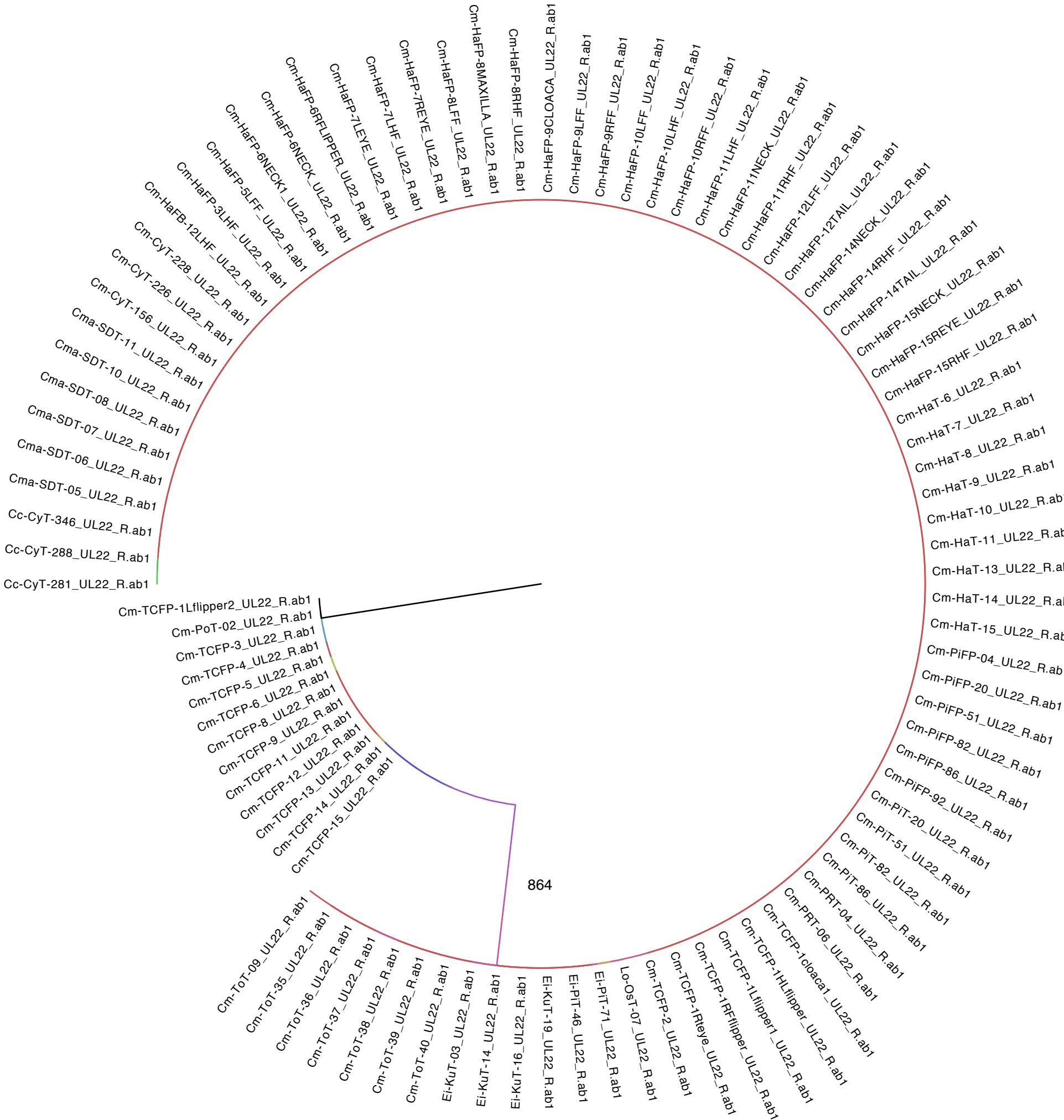

# UL27 global

C

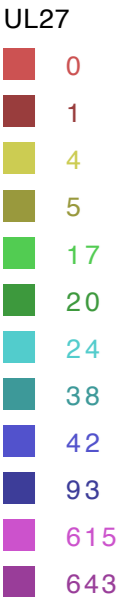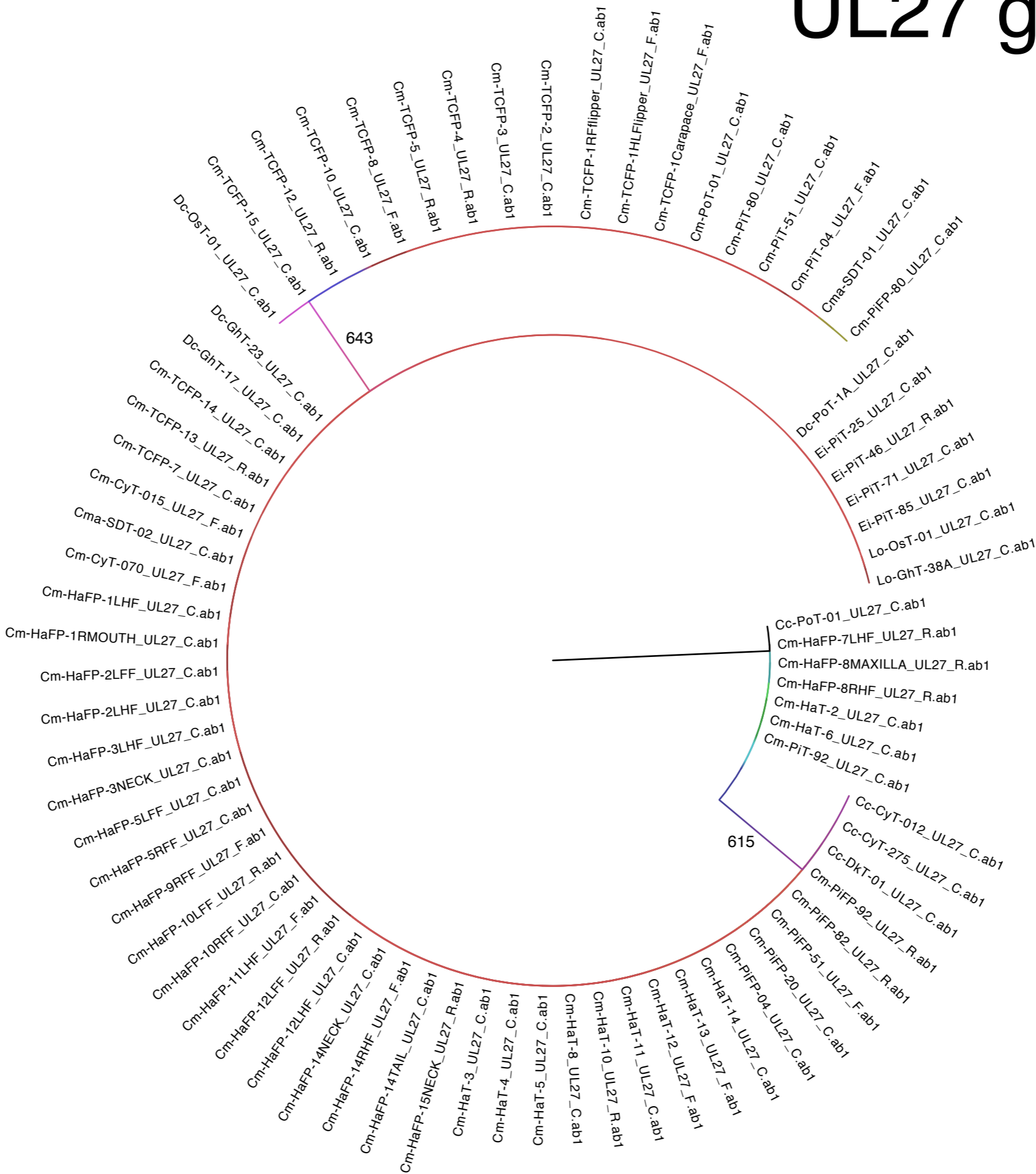

Supplement: Additional file 5: — Circular maximum likelihood phylogenetic trees; and Description of data. Circular maximum likelihood phylogenetic trees for (A) UL18, (B) UL22 and (C) UL27 sequence alignments generated in this study. [file 12862_2014_206_MOESM5_ESM.pdf]

# UL27 global + GenBank

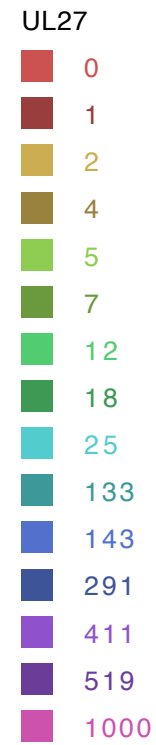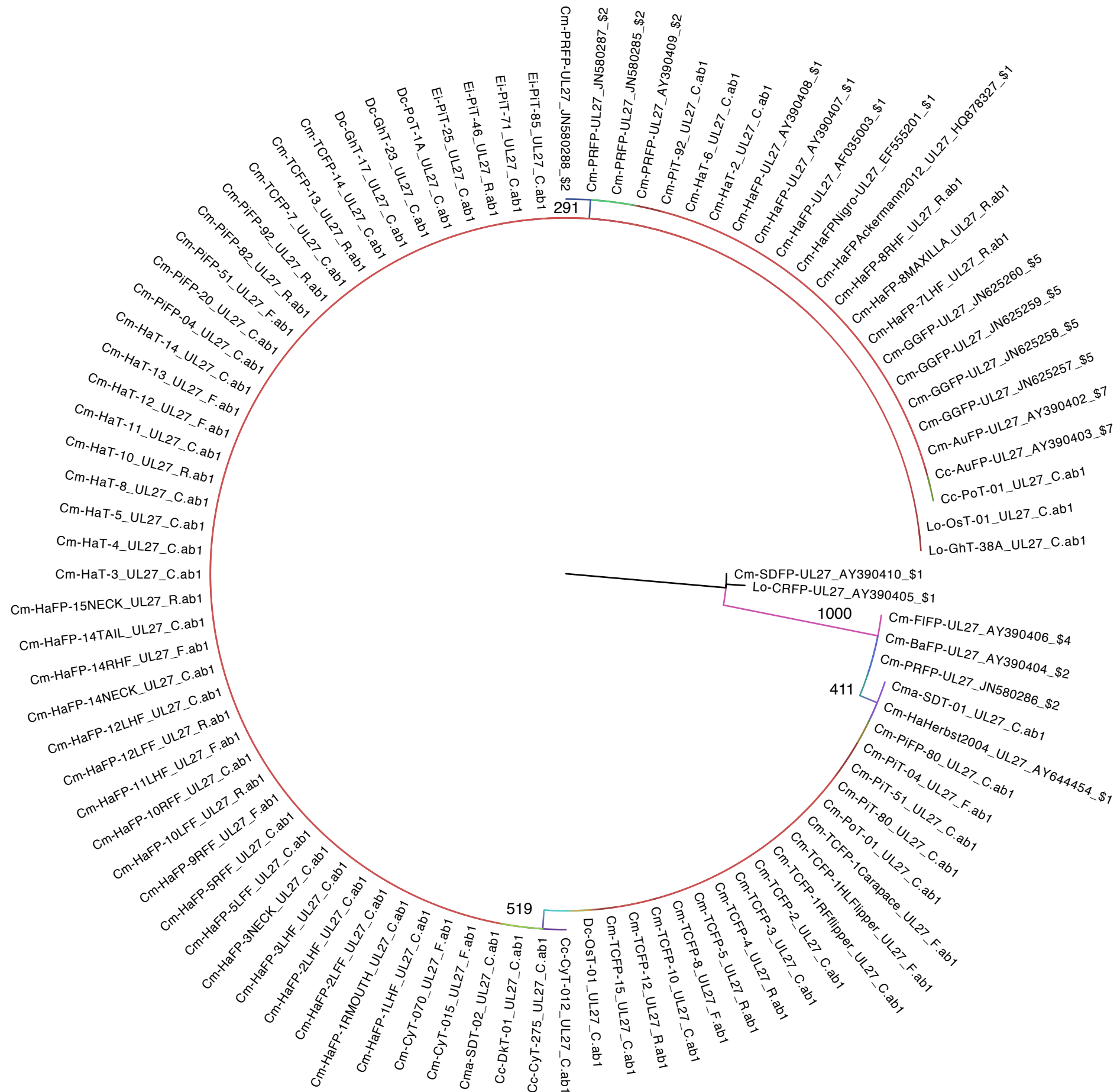

Supplement: Additional file 6: — Circular maximum likelihood phylogenetic tree for UL27 + GenBank; and Description of data. Circular maximum likelihood phylogenetic tree of combined alignment generated from UL27 sequences produced in this study and available sequences found at GenBank database. [file 12862_2014_206_MOESM6_ESM.pdf]
