# Supplementary material for: Global distribution of Chelonid fibropapilloma-associated herpesvirus among clinically healthy sea turtles
Source: BMC Evol Biol. 2014 Oct 25;14:206. doi: 10.1186/s12862-014-0206-z (PMC4219010; doi:10.1186/s12862-014-0206-z)
Supplement: Additional file 7: — Detailed sample material analysed for CFPHV detection; and Description of data. List of DNA extracts analysed for DNA viral detection of the Chelonid fibropapilloma-associated herpesvirus (CFPHV) including, sample ID, species, type of tissue, and population origin. Moreover, PCR assay results by individual marker and confirmation of CFPHV DNA by Sanger sequencing. [file 12862_2014_206_MOESM7_ESM.pdf]

Sample Code	Species	Type of sample	Sample origen site (population)	Viral detection by PCR assays					
				Singleplex primer set UL18 (140bp)	Confirmed DNA sequence (F= forward, R=reverse, C=consensus of both F and R)	Singleplex primer set UL22 (179bp)	Confirmed DNA sequence (F= forward, R=reverse, C=consensus of both F and R)	Singleplex primer set UL27 (143bp)	Confirmed DNA sequence (F= forward, R=reverse, C=consensus of both F and R)
Cc-DkT-01	loggerhead (*Caretta caretta*)	tissue	Denmark-Portugal, North Atlantic	+	C	-		+	C
Ah-DkT-1	*Agroniemus horsfieldii* (non sea turtle)	tissue	Denmark, Copenhagen Zoo	-		-		-	
Ca-DkT-1	*Cuora amboinensis* (non sea turtle)	tissue	Denmark, Copenhagen Zoo	-		-		-	
Ci-DkT-1	*Carettochelys insculpta* (non sea turtle)	tissue	Denmark, Copenhagen Zoo	-		-		-	
Gc-DkLe-1	*Geochelone carbonaria* (non sea turtle)	tissue lever	Denmark, Copenhagen Zoo	-		-		-	
Gp-DkT-1	*Geochelone pardalis* (non sea turtle)	tissue	Denmark, Copenhagen Zoo	-		-		-	
Gr-DtT-1	*Geochelone radiata* (non sea turtle)	tissue	Denmark, Copenhagen Zoo	-		-		-	
Ie-DkT-1	*Indotestudo elongata* (non sea turtle)	tissue	Denmark, Copenhagen Zoo	-		-		-	
Ie-DkT-2	*Indotestudo elongata* (non sea turtle)	tissue	Denmark, Copenhagen Zoo	-		-		-	
Pu-DkLe-2	*Podocnemis unifilis* (non sea turtle)	tissue lever	Denmark, Copenhagen Zoo	-		-		-	
Pu-DkT-1	*Podocnemis unifilis* (non sea turtle)	tissue	Denmark, Copenhagen Zoo	-		-		-	
Tg-DkT-1	*Testudo greca* (non sea turtle)	tissue	Denmark, Copenhagen Zoo	-		-		-	
Cm-DkT-01cyststom	green (*Chelonia mydas*)	tissue of cyst in stomach	Denmark, Danish aquarium	-		-		-	
Cm-DkT-01liver	green (*Chelonia mydas*)	tissue very bloody	Denmark, Danish aquarium	-		-		-	
Cm-DkT-01mouth	green (*Chelonia mydas*)	tissue	Denmark, Danish aquarium	-		-		-	
Cm-DkT-01neck	green (*Chelonia mydas*)	tissue	Denmark, Danish aquarium	-		-		-	
Cm-DkT-01rearLF	green (*Chelonia mydas*)	tissue	Denmark, Danish aquarium	-		-		-	
Cm-DbT-01	green (*Chelonia mydas*)	tissue front flipper	Dubai Aquarium, EAU	-		-		-	
Cm-DbT-02	green (*Chelonia mydas*)	tissue front flipper	Dubai Aquarium, EAU	-		-		-	
Ei-DbT-01	hawksbill (*Eretmochelys imbricata*)	tissue front flipper	Dubai Aquarium, EAU	-		-		-	
Ei-DbT-02	hawksbill (*Eretmochelys imbricata*)	tissue front flipper	Dubai Aquarium, EAU	-		-		-	
Ei-DbT-03	hawksbill (*Eretmochelys imbricata*)	tissue front flipper	Dubai Aquarium, EAU	-		-		-	
Ei-DbT-04	hawksbill (*Eretmochelys imbricata*)	tissue front flipper	Dubai Aquarium, EAU	-		-		-	
Ei-DbT-05	hawksbill (*Eretmochelys imbricata*)	tissue front flipper	Dubai Aquarium, EAU	-		-		-	
Ei-DbT-06	hawksbill (*Eretmochelys imbricata*)	tissue front flipper	Dubai Aquarium, EAU	-		-		-	
Ei-DbT-07	hawksbill (*Eretmochelys imbricata*)	tissue front flipper	Dubai Aquarium, EAU	-		-		-	
Ei-DbT-08	hawksbill (*Eretmochelys imbricata*)	tissue front flipper	Dubai Aquarium, EAU	-		-		-	
Ei-DbT-09	hawksbill (*Eretmochelys imbricata*)	tissue front flipper	Dubai Aquarium, EAU	-		-		-	
Lo-DbT-01	olive ridley (*Lepidochelys olivacea*)	tissue front flipper	Dubai Aquarium, EAU	-		-		-	
Dc-GhT-01	leatherback (*Dermochelys coriacea*)	tissue rear flipper	Ghana, West coast of Africa	-		-		-	
Dc-GhT-02	leatherback (*Dermochelys coriacea*)	tissue rear flipper	Ghana, West coast of Africa	-		-		-	
Dc-GhT-03	leatherback (*Dermochelys coriacea*)	tissue rear flipper	Ghana, West coast of Africa	-		-		-	
Dc-GhT-04	leatherback (*Dermochelys coriacea*)	tissue rear flipper	Ghana, West coast of Africa	-		-		-	
Dc-GhT-05	leatherback (*Dermochelys coriacea*)	tissue rear flipper	Ghana, West coast of Africa	-		-		-	
Dc-GhT-08	leatherback (*Dermochelys coriacea*)	tissue rear flipper	Ghana, West coast of Africa	-		-		-	
Dc-GhT-09	leatherback (*Dermochelys coriacea*)	tissue rear flipper	Ghana, West coast of Africa	-		-		-	
Dc-GhT-11	leatherback (*Dermochelys coriacea*)	tissue rear flipper	Ghana, West coast of Africa	-		-		-	
Dc-GhT-13B	leatherback (*Dermochelys coriacea*)	tissue rear flipper	Ghana, West coast of Africa	-		-		-	
Dc-GhT-14A	leatherback (*Dermochelys coriacea*)	tissue rear flipper	Ghana, West coast of Africa	-		-		-	
Dc-GhT-17	leatherback (*Dermochelys coriacea*)	tissue rear flipper	Ghana, West coast of Africa	-		-		+	C
Dc-GhT-18	leatherback (*Dermochelys coriacea*)	tissue rear flipper	Ghana, West coast of Africa	-		-		-	
Dc-GhT-21	leatherback (*Dermochelys coriacea*)	tissue rear flipper	Ghana, West coast of Africa	-		-		-	
Dc-GhT-21X	leatherback (*Dermochelys coriacea*)	tissue rear flipper	Ghana, West coast of Africa	-		-		-	
Dc-GhT-22	leatherback (*Dermochelys coriacea*)	tissue rear flipper	Ghana, West coast of Africa	-		-		-	
Dc-GhT-23	leatherback (*Dermochelys coriacea*)	tissue rear flipper	Ghana, West coast of Africa	-		-		+	C
Dc-GhT-24A	leatherback (*Dermochelys coriacea*)	tissue rear flipper	Ghana, West coast of Africa	-		-		-	
Lo-GhT-37	olive ridley (*Lepidochelys olivacea*)	tissue	Ghana, West coast of Africa	-		-		-	
Lo-GhT-38A	olive ridley (*Lepidochelys olivacea*)	tissue	Ghana, West coast of Africa	-		-		+	C
Lo-GhT-38B28	olive ridley (*Lepidochelys olivacea*)	tissue	Ghana, West coast of Africa	-		-		-	
Lo-GhT-39	olive ridley (*Lepidochelys olivacea*)	tissue	Ghana, West coast of Africa	-		-		-	
Lo-GhT-40	olive ridley (*Lepidochelys olivacea*)	tissue	Ghana, West coast of Africa	-		-		-	
Lo-GhT-41	olive ridley (*Lepidochelys olivacea*)	tissue	Ghana, West coast of Africa	-		-		-	
Lo-GhT-46	olive ridley (*Lepidochelys olivacea*)	tissue	Ghana, West coast of Africa	-		-		-	
Lo-GhT-47	olive ridley (*Lepidochelys olivacea*)	tissue	Ghana, West coast of Africa	-		-		-	
Lo-GhT-49	olive ridley (*Lepidochelys olivacea*)	tissue	Ghana, West coast of Africa	-		-		-	
Cma-HaFP-10LFF	green (*Chelonia mydas/agassizi*)	tumour left front flipper	Hawaii, North Pacific	+	R	+	R	+	R
Cma-HaFP-10LHF	green (*Chelonia mydas/agassizi*)	tumour left hind flipper	Hawaii, North Pacific	+	R	+	R	-	
Cma-HaFP-10RFF	green (*Chelonia mydas/agassizi*)	tumour right front flipper	Hawaii, North Pacific	+	R	+	R	+	C
Cma-HaFP-11LHF	green (*Chelonia mydas/agassizi*)	tumour left hind flipper	Hawaii, North Pacific	-		+	R	+	F
Cma-HaFP-11neck	green (*Chelonia mydas/agassizi*)	tumour neck	Hawaii, North Pacific	+	R	+	R	-	
Cma-HaFP-11RHF	green (*Chelonia mydas/agassizi*)	tumour right hind flipper	Hawaii, North Pacific	+	R	+	R		
Cma-HaFP-12LFF	green (*Chelonia mydas/agassizi*)	tumour left front flipper	Hawaii, North Pacific	+	R	+	R	+	R
Cma-HaFP-12LHF	green (*Chelonia mydas/agassizi*)	tumour left hind flipper	Hawaii, North Pacific	+	R	-		+	C
Cma-HaFP-12tail	green (*Chelonia mydas/agassizi*)	tumour tail	Hawaii, North Pacific	+	R	+	R	-	
Cma-HaFP-14neck	green (*Chelonia mydas/agassizi*)	tumour neck	Hawaii, North Pacific	-		+	R	+	C
Cma-HaFP-14RHF	green (*Chelonia mydas/agassizi*)	tumour right hind flipper	Hawaii, North Pacific	+	R	+	R	+	F
Cma-HaFP-14tail	green (*Chelonia mydas/agassizi*)	tumour tail	Hawaii, North Pacific	+	R	+	R	+	C
Cma-HaFP-15neck	green (*Chelonia mydas/agassizi*)	tumour neck	Hawaii, North Pacific	+	R	+	R	+	R
Cma-HaFP-15Reye	green (*Chelonia mydas/agassizi*)	tumour right eye	Hawaii, North Pacific	+	R	+	R	-	

Sample Code	Species	Type of sample	Sample origen site (population)	Viral detection by PCR assays					
				Singleplex primer set UL18 (140bp)	Confirmed DNA sequence (F= forward, R=reverse, C=consensus of both F and R)	Singleplex primer set UL22 (179bp)	Confirmed DNA sequence (F= forward, R=reverse, C=consensus of both F and R)	Singleplex primer set UL27 (143bp)	Confirmed DNA sequence (F= forward, R=reverse, C=consensus of both F and R)
Cma-HaFP-15RHF	green (*Chelonia mydas/agassizi*)	tumour right hind flipper	Hawaii, North Pacific	-		+	R	-	
Cma-HaFP-1LHF	green (*Chelonia mydas/agassizi*)	tumour left hind flipper	Hawaii, North Pacific	-		-		+	C
Cma-HaFP-1Rmouth	green (*Chelonia mydas/agassizi*)	tumour R mouth	Hawaii, North Pacific	+	R	-		+	C
Cma-HaFP-2LFF	green (*Chelonia mydas/agassizi*)	tumour left front flipper	Hawaii, North Pacific	+	R			+	C
Cma-HaFP-2LHF	green (*Chelonia mydas/agassizi*)	tumour left hind flipper	Hawaii, North Pacific	+	R			+	C
Cma-HaFP-3LHF	green (*Chelonia mydas/agassizi*)	tumour left hind flipper	Hawaii, North Pacific	-		+	R	+	C
Cma-HaFP-3neck	green (*Chelonia mydas/agassizi*)	tumour neck	Hawaii, North Pacific	+	R	-		+	C
Cma-HaFP-4neck	green (*Chelonia mydas/agassizi*)	tumour neck	Hawaii, North Pacific	+	R	-		-	
Cma-HaFP-5LFF	green (*Chelonia mydas/agassizi*)	tumour left front flipper	Hawaii, North Pacific	+	R	+	R	+	C
Cma-HaFP-5RFF	green (*Chelonia mydas/agassizi*)	tumour right front flipper	Hawaii, North Pacific	-		-		+	C
Cma-HaFP-6neck	green (*Chelonia mydas/agassizi*)	tumour neck	Hawaii, North Pacific	+	R	+	R	-	
Cma-HaFP-6neck1	green (*Chelonia mydas/agassizi*)	tumour neck1	Hawaii, North Pacific	+	R	+	R	-	
Cma-HaFP-6RFlipper	green (*Chelonia mydas/agassizi*)	tumour right flipper	Hawaii, North Pacific	+	R	+	R	-	
Cma-HaFP-7Leye	green (*Chelonia mydas/agassizi*)	tumour left eye	Hawaii, North Pacific	+	R	+	R	-	
Cma-HaFP-7LHF	green (*Chelonia mydas/agassizi*)	tumour left hind flipper	Hawaii, North Pacific	+	R	+	R	+	R
Cma-HaFP-7Reye	green (*Chelonia mydas/agassizi*)	tumour right eye	Hawaii, North Pacific	+	R	+	R	-	
Cma-HaFP-8LFF	green (*Chelonia mydas/agassizi*)	tumour left front flipper	Hawaii, North Pacific	+	B	+	R	-	
Cma-HaFP-8maxilla	green (*Chelonia mydas/agassizi*)	tumour maxilla	Hawaii, North Pacific	+	R	+	R	+	R
Cma-HaFP-8RHF	green (*Chelonia mydas/agassizi*)	tumour right hind flipper	Hawaii, North Pacific	+	R	+	R	+	R
Cma-HaFP-9cloaca	green (*Chelonia mydas/agassizi*)	tumour cloaca	Hawaii, North Pacific	-		+	R	-	
Cma-HaFP-9LFF	green (*Chelonia mydas/agassizi*)	tumour left front flipper	Hawaii, North Pacific	+	R	+	R	-	
Cma-HaFP-9RFF	green (*Chelonia mydas/agassizi*)	tumour right front flipper	Hawaii, North Pacific	+	R	+	R	+	F
Cma-HaT-1	green (*Chelonia mydas/agassizi*)	tissue	Hawaii, North Pacific	-		-		-	
Cma-HaT-10	green (*Chelonia mydas/agassizi*)	tissue	Hawaii, North Pacific	-		+	R	+	R
Cma-HaT-11	green (*Chelonia mydas/agassizi*)	tissue	Hawaii, North Pacific	+	R	+	R	+	C
Cma-HaT-12	green (*Chelonia mydas/agassizi*)	tissue	Hawaii, North Pacific	-		-		+	F
Cma-HaT-13	green (*Chelonia mydas/agassizi*)	tissue	Hawaii, North Pacific	-		+	R	+	F
Cma-HaT-14	green (*Chelonia mydas/agassizi*)	tissue	Hawaii, North Pacific	+	R	+	R	+	C
Cma-HaT-15	green (*Chelonia mydas/agassizi*)	tissue	Hawaii, North Pacific	-		+	R	-	
Cma-HaT-2	green (*Chelonia mydas/agassizi*)	tissue	Hawaii, North Pacific	+	R	-		+	C
Cma-HaT-3	green (*Chelonia mydas/agassizi*)	tissue	Hawaii, North Pacific	-		-		+	C
Cma-HaT-4	green (*Chelonia mydas/agassizi*)	tissue	Hawaii, North Pacific	-		-		+	C
Cma-HaT-5	green (*Chelonia mydas/agassizi*)	tissue	Hawaii, North Pacific	-		-		+	C
Cma-HaT-6	green (*Chelonia mydas/agassizi*)	tissue	Hawaii, North Pacific	-		+	R	+	C
Cma-HaT-7	green (*Chelonia mydas/agassizi*)	tissue	Hawaii, North Pacific	-		+	R	-	
Cma-HaT-8	green (*Chelonia mydas/agassizi*)	tissue	Hawaii, North Pacific	+	B	+	R	+	C
Cma-HaT-9	green (*Chelonia mydas/agassizi*)	tissue	Hawaii, North Pacific	-		+	R	-	
Cc-MaT-01	loggerhead (*Caretta caretta*)	tissue	Masirah, Oman	-		-		-	
Cc-MaT-02	loggerhead (*Caretta caretta*)	tissue	Masirah, Oman	-		-		-	
Cc-MaT-03	loggerhead (*Caretta caretta*)	tissue	Masirah, Oman	-		-		-	
Cc-MaT-04	loggerhead (*Caretta caretta*)	tissue	Masirah, Oman	-		-		-	
Cc-MaT-05	loggerhead (*Caretta caretta*)	tissue	Masirah, Oman	-		-		-	
Cc-MaT-06	loggerhead (*Caretta caretta*)	tissue	Masirah, Oman	-		-		-	
Cc-MaT-07	loggerhead (*Caretta caretta*)	tissue	Masirah, Oman	-		-		-	
Cc-MaT-07ab	loggerhead (*Caretta caretta*)	abnormal tissue	Masirah, Oman	-		-		-	
Cc-MaT-08	loggerhead (*Caretta caretta*)	tissue	Masirah, Oman	-		-		-	
Cc-MaT-08ab	loggerhead (*Caretta caretta*)	abnormal tissue	Masirah, Oman	-		-		-	
Cc-MaT-09	loggerhead (*Caretta caretta*)	tissue	Masirah, Oman	-		-		-	
Cc-MaT-10	loggerhead (*Caretta caretta*)	tissue	Masirah, Oman	-		-		-	
Cc-MaT-11	loggerhead (*Caretta caretta*)	tissue	Masirah, Oman	-		-		-	
Cc-MaT-12	loggerhead (*Caretta caretta*)	tissue	Masirah, Oman	-		-		-	
Cc-MaT-13	loggerhead (*Caretta caretta*)	tissue	Masirah, Oman	-		-		-	
Cc-MaT-14	loggerhead (*Caretta caretta*)	tissue	Masirah, Oman	-		-		-	
Cc-MaT-14ab	loggerhead (*Caretta caretta*)	abnormal tissue	Masirah, Oman	-		-		-	
Cc-MaT-15	loggerhead (*Caretta caretta*)	tissue	Masirah, Oman	-		-		-	
Cc-MaT-16	loggerhead (*Caretta caretta*)	tissue	Masirah, Oman	-		-		-	
Cc-MaT-17	loggerhead (*Caretta caretta*)	tissue	Masirah, Oman	-		-		-	
Cc-MaT-18	loggerhead (*Caretta caretta*)	tissue	Masirah, Oman	-		-		-	
Cc-MaT-19	loggerhead (*Caretta caretta*)	tissue	Masirah, Oman	-		-		-	
Cc-MaT-20k15	loggerhead (*Caretta caretta*)	tissue	Masirah, Oman	-		-		-	
Cc-MaT-20k15ab	loggerhead (*Caretta caretta*)	abnormal tissue	Masirah, Oman	-		-		-	
Cc-MaT-20k9	loggerhead (*Caretta caretta*)	tissue	Masirah, Oman	-		-		-	
Cc-MaT-21	loggerhead (*Caretta caretta*)	tissue	Masirah, Oman	-		-		-	
Cc-MaT-21ab	loggerhead (*Caretta caretta*)	abnormal tissue	Masirah, Oman	-		-		-	
Cc-MaT-22	loggerhead (*Caretta caretta*)	tissue	Masirah, Oman	-		-		-	
Cc-MaT-23	loggerhead (*Caretta caretta*)	tissue	Masirah, Oman	-		-		-	
Cc-MaT-25	loggerhead (*Caretta caretta*)	tissue	Masirah, Oman	-		-		-	
Cc-MaT-26	loggerhead (*Caretta caretta*)	tissue	Masirah, Oman	-		-		-	
Cc-MaT-27	loggerhead (*Caretta caretta*)	tissue	Masirah, Oman	-		-		-	
Cc-MaT-28	loggerhead (*Caretta caretta*)	tissue	Masirah, Oman	-		-		-	
Cc-MaT-29	loggerhead (*Caretta caretta*)	tissue	Masirah, Oman	-		-		-	
Cc-MaT-30	loggerhead (*Caretta caretta*)	tissue	Masirah, Oman	-		-		-	

				Viral detection by PCR assays					
Sample Code	Species	Type of sample	Sample origen site (population)	Singleplex primer set UL18 (140bp)	Confirmed DNA sequence (F= forward, R=reverse, C=consensus of both F and R)	Singleplex primer set UL22 (179bp)	Confirmed DNA sequence (F= forward, R=reverse, C=consensus of both F and R)	Singleplex primer set UL27 (143bp)	Confirmed DNA sequence (F= forward, R=reverse, C=consensus of both F and R)
Cc-MaT-31	loggerhead (*Caretta caretta*)	tissue	Masirah, Oman	-		-		-	
Cc-MaT-32	loggerhead (*Caretta caretta*)	tissue	Masirah, Oman	-		-		-	
Lo-MaT-01	olive ridley (*Lepidochelys olivacea*)	tissue	Masirah, Oman	-		-		-	
Cc-CyT-012	loggerhead (*Caretta caretta*)	tissue	Northen Cyprus, Mediterranean Sea	-		-		+	C
Cc-CyT-111	loggerhead (*Caretta caretta*)	tissue	Northen Cyprus, Mediterranean Sea	-		-		-	
Cc-CyT-192	loggerhead (*Caretta caretta*)	tissue	Northen Cyprus, Mediterranean Sea	-		-		-	
Cc-CyT-240	loggerhead (*Caretta caretta*)	tissue	Northen Cyprus, Mediterranean Sea	-		-		-	
Cc-CyT-255	loggerhead (*Caretta caretta*)	tissue	Northen Cyprus, Mediterranean Sea	-		-		-	
Cc-CyT-275	loggerhead (*Caretta caretta*)	tissue	Northen Cyprus, Mediterranean Sea	-		-		+	C
Cc-CyT-276	loggerhead (*Caretta caretta*)	tissue	Northen Cyprus, Mediterranean Sea	-		-		-	
Cc-CyT-281	loggerhead (*Caretta caretta*)	tissue	Northen Cyprus, Mediterranean Sea	-		+	R	-	
Cc-CyT-288	loggerhead (*Caretta caretta*)	tissue	Northen Cyprus, Mediterranean Sea	-		+	R	-	
Cc-CyT-327	loggerhead (*Caretta caretta*)	tissue	Northen Cyprus, Mediterranean Sea	-		-		-	
Cc-CyT-329	loggerhead (*Caretta caretta*)	tissue	Northen Cyprus, Mediterranean Sea	-		-		-	
Cc-CyT-331	loggerhead (*Caretta caretta*)	tissue	Northen Cyprus, Mediterranean Sea	-		-		-	
Cc-CyT-334	loggerhead (*Caretta caretta*)	tissue	Northen Cyprus, Mediterranean Sea	-		-		-	
Cc-CyT-335	loggerhead (*Caretta caretta*)	tissue	Northen Cyprus, Mediterranean Sea	-		-		-	
Cc-CyT-336	loggerhead (*Caretta caretta*)	tissue	Northen Cyprus, Mediterranean Sea	-		-		-	
Cc-CyT-338	loggerhead (*Caretta caretta*)	tissue	Northen Cyprus, Mediterranean Sea	-		-		-	
Cc-CyT-339	loggerhead (*Caretta caretta*)	tissue	Northen Cyprus, Mediterranean Sea	-		-		-	
Cc-CyT-340	loggerhead (*Caretta caretta*)	tissue	Northen Cyprus, Mediterranean Sea	-		-		-	
Cc-CyT-341	loggerhead (*Caretta caretta*)	tissue	Northen Cyprus, Mediterranean Sea	-		-		-	
Cc-CyT-342	loggerhead (*Caretta caretta*)	tissue	Northen Cyprus, Mediterranean Sea	-		-		-	
Cc-CyT-343	loggerhead (*Caretta caretta*)	tissue	Northen Cyprus, Mediterranean Sea	-		-		-	
Cc-CyT-345	loggerhead (*Caretta caretta*)	tissue	Northen Cyprus, Mediterranean Sea	-		-		-	
Cc-CyT-346	loggerhead (*Caretta caretta*)	tissue	Northen Cyprus, Mediterranean Sea	-		+	R	-	
Cc-CyT-349	loggerhead (*Caretta caretta*)	tissue	Northen Cyprus, Mediterranean Sea	-		-		-	
Cm-CyT-015	green (*Chelonia mydas*)	tissue	Northen Cyprus, Mediterranean Sea	-		-		+	F
Cm-CyT-070	green (*Chelonia mydas*)	tissue	Northen Cyprus, Mediterranean Sea	-		-		+	F
Cm-CyT-071	green (*Chelonia mydas*)	tissue	Northen Cyprus, Mediterranean Sea	-		-		-	
Cm-CyT-156	green (*Chelonia mydas*)	tissue	Northen Cyprus, Mediterranean Sea	-		+	R	-	
Cm-CyT-157	green (*Chelonia mydas*)	tissue	Northen Cyprus, Mediterranean Sea	-		-		-	
Cm-CyT-166	green (*Chelonia mydas*)	tissue	Northen Cyprus, Mediterranean Sea	-		-		-	
Cm-CyT-183	green (*Chelonia mydas*)	tissue	Northen Cyprus, Mediterranean Sea	-		-		-	
Cm-CyT-185	green (*Chelonia mydas*)	tissue	Northen Cyprus, Mediterranean Sea	-		-		-	
Cm-CyT-191	green (*Chelonia mydas*)	tissue	Northen Cyprus, Mediterranean Sea	-		-		-	
Cm-CyT-205	green (*Chelonia mydas*)	tissue	Northen Cyprus, Mediterranean Sea	-		-		-	
Cm-CyT-206	green (*Chelonia mydas*)	tissue	Northen Cyprus, Mediterranean Sea	-		-		-	
Cm-CyT-208	green (*Chelonia mydas*)	tissue	Northen Cyprus, Mediterranean Sea	-		-		-	
Cm-CyT-209	green (*Chelonia mydas*)	tissue	Northen Cyprus, Mediterranean Sea	-		-		-	
Cm-CyT-210	green (*Chelonia mydas*)	tissue	Northen Cyprus, Mediterranean Sea	-		-		-	
Cm-CyT-211	green (*Chelonia mydas*)	tissue	Northen Cyprus, Mediterranean Sea	-		-		-	
Cm-CyT-212	green (*Chelonia mydas*)	tissue	Northen Cyprus, Mediterranean Sea	-		-		-	
Cm-CyT-213	green (*Chelonia mydas*)	tissue	Northen Cyprus, Mediterranean Sea	-		-		-	
Cm-CyT-214	green (*Chelonia mydas*)	tissue	Northen Cyprus, Mediterranean Sea	-		-		-	
Cm-CyT-216	green (*Chelonia mydas*)	tissue	Northen Cyprus, Mediterranean Sea	-		-		-	
Cm-CyT-218	green (*Chelonia mydas*)	tissue	Northen Cyprus, Mediterranean Sea	-		-		-	
Cm-CyT-221	green (*Chelonia mydas*)	tissue	Northen Cyprus, Mediterranean Sea	-		-		-	
Cm-CyT-222	green (*Chelonia mydas*)	tissue	Northen Cyprus, Mediterranean Sea	-		-		-	
Cm-CyT-223	green (*Chelonia mydas*)	tissue	Northen Cyprus, Mediterranean Sea	-		-		-	
Cm-CyT-225	green (*Chelonia mydas*)	tissue	Northen Cyprus, Mediterranean Sea	-		-		-	
Cm-CyT-226	green (*Chelonia mydas*)	tissue	Northen Cyprus, Mediterranean Sea	-		+	R	-	
Cm-CyT-228	green (*Chelonia mydas*)	tissue	Northen Cyprus, Mediterranean Sea	-		+	R	-	
Cc-CaT-01	loggerhead (*Caretta caretta*)	DNA-assumed taken from normal tissue	Noth Pacific, California	-		-		-	
Dc-OsT-01	leatherback (*Dermochelys coriacea*)	tissue rear flipper	Ostional, Pacific of Costa Rica	+	C	-		+	C
Lo-OsT-01	olive ridley (*Lepidochelys olivacea*)	tissue rear flipper	Ostional, Pacific of Costa Rica	-		-		+	C
Lo-OsT-02	olive ridley (*Lepidochelys olivacea*)	tissue rear flipper	Ostional, Pacific of Costa Rica	-		-		-	
Lo-OsT-03	olive ridley (*Lepidochelys olivacea*)	tissue rear flipper	Ostional, Pacific of Costa Rica	-		-		-	
Lo-OsT-04	olive ridley (*Lepidochelys olivacea*)	tissue rear flipper	Ostional, Pacific of Costa Rica	-		-		-	
Lo-OsT-05	olive ridley (*Lepidochelys olivacea*)	tissue rear flipper	Ostional, Pacific of Costa Rica	-		-		-	
Lo-OsT-06	olive ridley (*Lepidochelys olivacea*)	tissue rear flipper	Ostional, Pacific of Costa Rica	-		-		-	
Lo-OsT-07	olive ridley (*Lepidochelys olivacea*)	tissue rear flipper	Ostional, Pacific of Costa Rica	-		+	R	-	
Lo-OsT-08	olive ridley (*Lepidochelys olivacea*)	tissue rear flipper	Ostional, Pacific of Costa Rica	-		-		-	
Lo-OsT-09	olive ridley (*Lepidochelys olivacea*)	tissue rear flipper	Ostional, Pacific of Costa Rica	-		-		-	
Dc-PoT-1A	leatherback (*Dermochelys coriacea*)	tissue	Portugal, North Atlantic	-		-		+	C
Dc-PoT-1B	leatherback (*Dermochelys coriacea*)	tissue	Portugal, North Atlantic	-		-		-	
Cc-PoT-01	loggerhead (*Caretta caretta*)	tissue and fat	Portugal, Zoomarine	-		-		+	C
Cm-PoT-01	green (*Chelonia mydas*)	tissue and fat	Portugal, Zoomarine	+	C	-		+	C
Cm-PoT-02	green (*Chelonia mydas*)	tissue and fat	Portugal, Zoomarine	-		+	R	-	
Cm-PiFP-04	green (*Chelonia mydas*)	tumour FP	Príncipe Island, West coast of Africa	+	R	+	R	+	C
Cm-PiFP-20	green (*Chelonia mydas*)	tumour FP	Príncipe Island, West coast of Africa	-		+	R	+	C
Cm-PiFP-51	green (*Chelonia mydas*)	tumour FP	Príncipe Island, West coast of Africa	+	R	+	R	+	F

				Viral detection by PCR assays					
Sample Code	Species	Type of sample	Sample origen site (population)	Singleplex primer set UL18 (140bp)	Confirmed DNA sequence (F= forward, R=reverse, C=consensus of both F and R)	Singleplex primer set UL22 (179bp)	Confirmed DNA sequence (F= forward, R=reverse, C=consensus of both F and R)	Singleplex primer set UL27 (143bp)	Confirmed DNA sequence (F= forward, R=reverse, C=consensus of both F and R)
Cm-PiFP-80	green (*Chelonia mydas*)	tumour FP	Príncipe Island, West coast of Africa	+	C	-		+	C
Cm-PiFP-82	green (*Chelonia mydas*)	tumour FP	Príncipe Island, West coast of Africa	-		+	R	+	R
Cm-PiFP-86	green (*Chelonia mydas*)	tumour FP	Príncipe Island, West coast of Africa	+	R	+	R	-	
Cm-PiFP-92	green (*Chelonia mydas*)	tumour FP	Príncipe Island, West coast of Africa	+	R	+	R	+	R
Cm-PiT-04	green (*Chelonia mydas*)	tissue	Príncipe Island, West coast of Africa	-		-		+	F
Cm-PiT-20	green (*Chelonia mydas*)	tissue	Príncipe Island, West coast of Africa	+	R	+	R	-	
Cm-PiT-51	green (*Chelonia mydas*)	tissue	Príncipe Island, West coast of Africa	+	R	+	R	+	C
Cm-PiT-80	green (*Chelonia mydas*)	tissue	Príncipe Island, West coast of Africa	-		-		+	C
Cm-PiT-82	green (*Chelonia mydas*)	tissue	Príncipe Island, West coast of Africa	-		+	R	-	
Cm-PiT-86	green (*Chelonia mydas*)	tissue	Príncipe Island, West coast of Africa	-		+	R	-	
Cm-PiT-92	green (*Chelonia mydas*)	tissue	Príncipe Island, West coast of Africa	-		-		+	C
Ei-PiT-25	hawksbill (*Eretmochelys imbricata*)	tissue ID 380	Príncipe Island, West coast of Africa	-		-		+	C
Ei-PiT-46	hawksbill (*Eretmochelys imbricata*)	tissue	Príncipe Island, West coast of Africa	+	R	+	R	+	R
Ei-PiT-71	hawksbill (*Eretmochelys imbricata*)	tissue ID 430	Príncipe Island, West coast of Africa	+	R	+	R	+	C
Ei-PiT-85	hawksbill (*Eretmochelys imbricata*)	tissue ID 200	Príncipe Island, West coast of Africa	+	C	-		+	C
Cm-PRT-01	green (*Chelonia mydas*)	tissue	Puerto Rico, Caribbean	-		-		-	
Cm-PRT-02	green (*Chelonia mydas*)	tissue	Puerto Rico, Caribbean	-		-		-	
Cm-PRT-03	green (*Chelonia mydas*)	tissue	Puerto Rico, Caribbean	-		-		-	
Cm-PRT-04	green (*Chelonia mydas*)	tissue	Puerto Rico, Caribbean	-		+	R	-	
Cm-PRT-05	green (*Chelonia mydas*)	tissue	Puerto Rico, Caribbean	-		-		-	
Cm-PRT-06	green (*Chelonia mydas*)	tissue	Puerto Rico, Caribbean	-		+	R	-	
Ei-PRT-01	hawksbill (*Eretmochelys imbricata*)	tissue	Puerto Rico, Caribbean	-		-		-	
Ei-PRT-02	hawksbill (*Eretmochelys imbricata*)	tissue	Puerto Rico, Caribbean	-		-		-	
Ei-PRT-03	hawksbill (*Eretmochelys imbricata*)	tissue	Puerto Rico, Caribbean	-		-		-	
Ei-PRT-04	hawksbill (*Eretmochelys imbricata*)	tissue	Puerto Rico, Caribbean	-		-		-	
Ei-PRT-05	hawksbill (*Eretmochelys imbricata*)	tissue	Puerto Rico, Caribbean	-		-		-	
Cm-KuB-04	green (*Chelonia mydas*)	blood	Qaru Island, Kuwait	-		-		-	
Cm-KuT-01	green (*Chelonia mydas*)	tissue front flipper	Qaru Island, Kuwait	-		-		-	
Cm-KuT-02	green (*Chelonia mydas*)	tissue front flipper	Qaru Island, Kuwait	-		-		-	
Cm-KuT-03	green (*Chelonia mydas*)	tissue front flipper	Qaru Island, Kuwait	-		-		-	
Cm-KuT-04 (5)	green (*Chelonia mydas*)	tissue front flipper	Qaru Island, Kuwait	-		-		-	
Cm-KuT-05	green (*Chelonia mydas*)	tissue front flipper	Qaru Island, Kuwait	-		-		-	
Cm-KuT-07	green (*Chelonia mydas*)	tissue front flipper	Qaru Island, Kuwait	-		-		-	
Cm-KuT-08 (6)	green (*Chelonia mydas*)	tissue front flipper	Qaru Island, Kuwait	-		-		-	
Ei-KuT-10	hawksbill (*Eretmochelys imbricata*)	tissue	Qaru Island, Kuwait	-		-		-	
Ei-KuT-11	hawksbill (*Eretmochelys imbricata*)	tissue	Qaru Island, Kuwait	-		-		-	
Ei-KuT-12	hawksbill (*Eretmochelys imbricata*)	tissue	Qaru Island, Kuwait	-		-		-	
Ei-KuT-13	hawksbill (*Eretmochelys imbricata*)	tissue	Qaru Island, Kuwait	-		-		-	
Ei-KuT-14	hawksbill (*Eretmochelys imbricata*)	tissue	Qaru Island, Kuwait	-		+	R	-	
Ei-KuT-15	hawksbill (*Eretmochelys imbricata*)	tissue	Qaru Island, Kuwait	-		-		-	
Ei-KuT-16	hawksbill (*Eretmochelys imbricata*)	tissue	Qaru Island, Kuwait	-		+	R	-	
Ei-KuT-17	hawksbill (*Eretmochelys imbricata*)	tissue	Qaru Island, Kuwait	-		-		-	
Ei-KuT-18	hawksbill (*Eretmochelys imbricata*)	tissue	Qaru Island, Kuwait	-		-		-	
Ei-KuT-19	hawksbill (*Eretmochelys imbricata*)	tissue	Qaru Island, Kuwait	+	R	+	R	-	
Cma-SDT-01	green (*Chelonia mydas/agassizi*)	DNA-assumed taken from normal tissue	San Diego Bay, California, Pacific	-		-		+	C
Cma-SDT-01a	green (*Chelonia mydas/agassizi*)	DNA-assumed taken from normal tissue	San Diego Bay, California, Pacific	-		-		-	
Cma-SDT-02	green (*Chelonia mydas/agassizi*)	DNA-assumed taken from normal tissue	San Diego Bay, California, Pacific	-		-		+	C
Cma-SDT-03	green (*Chelonia mydas/agassizi*)	DNA-assumed taken from normal tissue	San Diego Bay, California, Pacific	-		-		-	
Cma-SDT-04	green (*Chelonia mydas/agassizi*)	DNA-assumed taken from normal tissue	San Diego Bay, California, Pacific	-		-		-	
Cma-SDT-05	green (*Chelonia mydas/agassizi*)	DNA-assumed taken from normal tissue	San Diego Bay, California, Pacific	-		+	R	-	
Cma-SDT-06	green (*Chelonia mydas/agassizi*)	DNA-assumed taken from normal tissue	San Diego Bay, California, Pacific	-		+	R	-	
Cma-SDT-07	green (*Chelonia mydas/agassizi*)	DNA-assumed taken from normal tissue	San Diego Bay, California, Pacific	-		+	R	-	
Cma-SDT-08	green (*Chelonia mydas/agassizi*)	DNA-assumed taken from normal tissue	San Diego Bay, California, Pacific	-		+	R	-	
Cma-SDT-09	green (*Chelonia mydas/agassizi*)	DNA-assumed taken from normal tissue	San Diego Bay, California, Pacific	-		-		-	
Cma-SDT-10	green (*Chelonia mydas/agassizi*)	DNA-assumed taken from normal tissue	San Diego Bay, California, Pacific	-		+	R	-	
Cma-SDT-11	green (*Chelonia mydas/agassizi*)	DNA-assumed taken from normal tissue	San Diego Bay, California, Pacific	-		+	R	-	
Cma-SDT-12	green (*Chelonia mydas/agassizi*)	DNA-assumed taken from normal tissue	San Diego Bay, California, Pacific	-		-		-	
Cma-SDT-13	green (*Chelonia mydas/agassizi*)	DNA-assumed taken from normal tissue	San Diego Bay, California, Pacific	-		-		-	
Cma-SDT-14	green (*Chelonia mydas/agassizi*)	DNA-assumed taken from normal tissue	San Diego Bay, California, Pacific	-		-		-	
Cma-SDT-15	green (*Chelonia mydas/agassizi*)	DNA-assumed taken from normal tissue	San Diego Bay, California, Pacific	-		-		-	
Cma-SDT-16	green (*Chelonia mydas/agassizi*)	DNA-assumed taken from normal tissue	San Diego Bay, California, Pacific	-		-		-	
Cma-SDT-17	green (*Chelonia mydas/agassizi*)	DNA-assumed taken from normal tissue	San Diego Bay, California, Pacific	-		-		-	
Cma-SDT-18	green (*Chelonia mydas/agassizi*)	DNA-assumed taken from normal tissue	San Diego Bay, California, Pacific	-		-		-	
Cma-SDT-19	green (*Chelonia mydas/agassizi*)	DNA-assumed taken from normal tissue	San Diego Bay, California, Pacific	-		-		-	
Cma-SDT-20	green (*Chelonia mydas/agassizi*)	DNA-assumed taken from normal tissue	San Diego Bay, California, Pacific	-		-		-	
Cma-SDT-21	green (*Chelonia mydas/agassizi*)	DNA-assumed taken from normal tissue	San Diego Bay, California, Pacific	-		-		-	
Cma-SDT-22	green (*Chelonia mydas/agassizi*)	DNA-assumed taken from normal tissue	San Diego Bay, California, Pacific	-		-		-	
Cma-SDT-23	green (*Chelonia mydas/agassizi*)	DNA-assumed taken from normal tissue	San Diego Bay, California, Pacific	-		-		-	
Cma-SDT-24	green (*Chelonia mydas/agassizi*)	DNA-assumed taken from normal tissue	San Diego Bay, California, Pacific	-		-		-	
Cma-SDT-25	green (*Chelonia mydas/agassizi*)	DNA-assumed taken from normal tissue	San Diego Bay, California, Pacific	-		-		-	
Cma-SDT-26	green (*Chelonia mydas/agassizi*)	DNA-assumed taken from normal tissue	San Diego Bay, California, Pacific	-		-		-	

				Viral detection by PCR assays					
**Sample Code**	**Species**	**Type of sample**	**Sample origen site (population)**	**Singleplex primer set UL18 (140bp)**	**Confirmed DNA sequence (F= forward, R=reverse, C=consensus of both F and R)**	**Singleplex primer set UL22 (179bp)**	**Confirmed DNA sequence (F= forward, R=reverse, C=consensus of both F and R)**	**Singleplex primer set UL27 (143bp)**	**Confirmed DNA sequence (F= forward, R=reverse, C=consensus of both F and R)**
Cma-SDT-27	green (*Chelonia mydas/agassizi*)	DNA-assumed taken from normal tissue	San Diego Bay, California, Pacific	-		-		-	
Cm-ToT-01	green (*Chelonia mydas*)	tissue rear flipper	Tortuguero, Caribbean of Costa Rica	-		-		-	
Cm-ToT-01_1	green (*Chelonia mydas*)	carapace soft tissue of hatchling	Tortuguero, Caribbean of Costa Rica	-		-		-	
Cm-ToT-01_10	green (*Chelonia mydas*)	carapace soft tissue of hatchling	Tortuguero, Caribbean of Costa Rica	-		-		-	
Cm-ToT-01_2	green (*Chelonia mydas*)	carapace soft tissue of hatchling	Tortuguero, Caribbean of Costa Rica	-		-		-	
Cm-ToT-01_3	green (*Chelonia mydas*)	carapace soft tissue of hatchling	Tortuguero, Caribbean of Costa Rica	-		-		-	
Cm-ToT-01_4	green (*Chelonia mydas*)	carapace soft tissue of hatchling	Tortuguero, Caribbean of Costa Rica	-		-		-	
Cm-ToT-01_5	green (*Chelonia mydas*)	carapace soft tissue of hatchling	Tortuguero, Caribbean of Costa Rica	-		-		-	
Cm-ToT-01_6	green (*Chelonia mydas*)	carapace soft tissue of hatchling	Tortuguero, Caribbean of Costa Rica	-		-		-	
Cm-ToT-01_7	green (*Chelonia mydas*)	carapace soft tissue of hatchling	Tortuguero, Caribbean of Costa Rica	-		-		-	
Cm-ToT-01_8	green (*Chelonia mydas*)	carapace soft tissue of hatchling	Tortuguero, Caribbean of Costa Rica	-		-		-	
Cm-ToT-01_9	green (*Chelonia mydas*)	carapace soft tissue of hatchling	Tortuguero, Caribbean of Costa Rica	-		-		-	
Cm-ToT-02	green (*Chelonia mydas*)	tissue rear flipper	Tortuguero, Caribbean of Costa Rica	-		-		-	
Cm-ToT-03	green (*Chelonia mydas*)	tissue rear flipper	Tortuguero, Caribbean of Costa Rica	-		-		-	
Cm-ToT-04	green (*Chelonia mydas*)	tissue rear flipper	Tortuguero, Caribbean of Costa Rica	-		-		-	
Cm-ToT-04_1	green (*Chelonia mydas*)	carapace soft tissue of hatchling	Tortuguero, Caribbean of Costa Rica	-		-		-	
Cm-ToT-04_10	green (*Chelonia mydas*)	carapace soft tissue of hatchling	Tortuguero, Caribbean of Costa Rica	-		-		-	
Cm-ToT-04_2	green (*Chelonia mydas*)	carapace soft tissue of hatchling	Tortuguero, Caribbean of Costa Rica	-		-		-	
Cm-ToT-04_3	green (*Chelonia mydas*)	carapace soft tissue of hatchling	Tortuguero, Caribbean of Costa Rica	-		-		-	
Cm-ToT-04_5	green (*Chelonia mydas*)	carapace soft tissue of hatchling	Tortuguero, Caribbean of Costa Rica	-		-		-	
Cm-ToT-04_6	green (*Chelonia mydas*)	carapace soft tissue of hatchling	Tortuguero, Caribbean of Costa Rica	-		-		-	
Cm-ToT-04_7	green (*Chelonia mydas*)	carapace soft tissue of hatchling	Tortuguero, Caribbean of Costa Rica	-		-		-	
Cm-ToT-04_8	green (*Chelonia mydas*)	carapace soft tissue of hatchling	Tortuguero, Caribbean of Costa Rica	-		-		-	
Cm-ToT-04_9	green (*Chelonia mydas*)	carapace soft tissue of hatchling	Tortuguero, Caribbean of Costa Rica	-		-		-	
Cm-ToT-05	green (*Chelonia mydas*)	tissue rear flipper	Tortuguero, Caribbean of Costa Rica	-		-		-	
Cm-ToT-05_1	green (*Chelonia mydas*)	carapace soft tissue of hatchling	Tortuguero, Caribbean of Costa Rica	-		-		-	
Cm-ToT-05_10	green (*Chelonia mydas*)	carapace soft tissue of hatchling	Tortuguero, Caribbean of Costa Rica	-		-		-	
Cm-ToT-05_2	green (*Chelonia mydas*)	carapace soft tissue of hatchling	Tortuguero, Caribbean of Costa Rica	-		-		-	
Cm-ToT-05_3	green (*Chelonia mydas*)	carapace soft tissue of hatchling	Tortuguero, Caribbean of Costa Rica	-		-		-	
Cm-ToT-05_4	green (*Chelonia mydas*)	carapace soft tissue of hatchling	Tortuguero, Caribbean of Costa Rica	-		-		-	
Cm-ToT-05_5	green (*Chelonia mydas*)	carapace soft tissue of hatchling	Tortuguero, Caribbean of Costa Rica	-		-		-	
Cm-ToT-05_6	green (*Chelonia mydas*)	carapace soft tissue of hatchling	Tortuguero, Caribbean of Costa Rica	-		-		-	
Cm-ToT-05_7	green (*Chelonia mydas*)	carapace soft tissue of hatchling	Tortuguero, Caribbean of Costa Rica	-		-		-	
Cm-ToT-05_8	green (*Chelonia mydas*)	carapace soft tissue of hatchling	Tortuguero, Caribbean of Costa Rica	-		-		-	
Cm-ToT-05_9	green (*Chelonia mydas*)	carapace soft tissue of hatchling	Tortuguero, Caribbean of Costa Rica	-		-		-	
Cm-ToT-06	green (*Chelonia mydas*)	tissue rear flipper	Tortuguero, Caribbean of Costa Rica	-		-		-	
Cm-ToT-07	green (*Chelonia mydas*)	tissue rear flipper	Tortuguero, Caribbean of Costa Rica	-		-		-	
Cm-ToT-08	green (*Chelonia mydas*)	tissue rear flipper	Tortuguero, Caribbean of Costa Rica	-		-		-	
Cm-ToT-08_1	green (*Chelonia mydas*)	carapace soft tissue of hatchling	Tortuguero, Caribbean of Costa Rica	-		-		-	
Cm-ToT-08_10	green (*Chelonia mydas*)	carapace soft tissue of hatchling	Tortuguero, Caribbean of Costa Rica	-		-		-	
Cm-ToT-08_2	green (*Chelonia mydas*)	carapace soft tissue of hatchling	Tortuguero, Caribbean of Costa Rica	-		-		-	
Cm-ToT-08_3	green (*Chelonia mydas*)	carapace soft tissue of hatchling	Tortuguero, Caribbean of Costa Rica	-		-		-	
Cm-ToT-08_4	green (*Chelonia mydas*)	carapace soft tissue of hatchling	Tortuguero, Caribbean of Costa Rica	-		-		-	
Cm-ToT-08_5	green (*Chelonia mydas*)	carapace soft tissue of hatchling	Tortuguero, Caribbean of Costa Rica	-		-		-	
Cm-ToT-08_6	green (*Chelonia mydas*)	carapace soft tissue of hatchling	Tortuguero, Caribbean of Costa Rica	-		-		-	
Cm-ToT-08_7	green (*Chelonia mydas*)	carapace soft tissue of hatchling	Tortuguero, Caribbean of Costa Rica	-		-		-	
Cm-ToT-08_8	green (*Chelonia mydas*)	carapace soft tissue of hatchling	Tortuguero, Caribbean of Costa Rica	-		-		-	
Cm-ToT-08_9	green (*Chelonia mydas*)	carapace soft tissue of hatchling	Tortuguero, Caribbean of Costa Rica	-		-		-	
Cm-ToT-09	green (*Chelonia mydas*)	tissue rear flipper	Tortuguero, Caribbean of Costa Rica	-		+	R	-	
Cm-ToT-09_1	green (*Chelonia mydas*)	carapace soft tissue of hatchling	Tortuguero, Caribbean of Costa Rica	-		-		-	
Cm-ToT-09_10	green (*Chelonia mydas*)	carapace soft tissue of hatchling	Tortuguero, Caribbean of Costa Rica	-		-		-	
Cm-ToT-09_2	green (*Chelonia mydas*)	carapace soft tissue of hatchling	Tortuguero, Caribbean of Costa Rica	-		-		-	
Cm-ToT-09_3	green (*Chelonia mydas*)	carapace soft tissue of hatchling	Tortuguero, Caribbean of Costa Rica	-		-		-	
Cm-ToT-09_4	green (*Chelonia mydas*)	carapace soft tissue of hatchling	Tortuguero, Caribbean of Costa Rica	-		-		-	
Cm-ToT-09_5	green (*Chelonia mydas*)	carapace soft tissue of hatchling	Tortuguero, Caribbean of Costa Rica	-		-		-	
Cm-ToT-09_6	green (*Chelonia mydas*)	carapace soft tissue of hatchling	Tortuguero, Caribbean of Costa Rica	-		-		-	
Cm-ToT-09_7	green (*Chelonia mydas*)	carapace soft tissue of hatchling	Tortuguero, Caribbean of Costa Rica	-		-		-	
Cm-ToT-09_8	green (*Chelonia mydas*)	carapace soft tissue of hatchling	Tortuguero, Caribbean of Costa Rica	-		-		-	
Cm-ToT-09_9	green (*Chelonia mydas*)	carapace soft tissue of hatchling	Tortuguero, Caribbean of Costa Rica	-		-		-	
Cm-ToT-10	green (*Chelonia mydas*)	tissue rear flipper	Tortuguero, Caribbean of Costa Rica	-		-		-	
Cm-ToT-11	green (*Chelonia mydas*)	tissue rear flipper	Tortuguero, Caribbean of Costa Rica	-		-		-	
Cm-ToT-11_1	green (*Chelonia mydas*)	carapace soft tissue of hatchling	Tortuguero, Caribbean of Costa Rica	-		-		-	
Cm-ToT-11_10	green (*Chelonia mydas*)	carapace soft tissue of hatchling	Tortuguero, Caribbean of Costa Rica	-		-		-	
Cm-ToT-11_2	green (*Chelonia mydas*)	carapace soft tissue of hatchling	Tortuguero, Caribbean of Costa Rica	-		-		-	
Cm-ToT-11_3	green (*Chelonia mydas*)	carapace soft tissue of hatchling	Tortuguero, Caribbean of Costa Rica	-		-		-	
Cm-ToT-11_4	green (*Chelonia mydas*)	carapace soft tissue of hatchling	Tortuguero, Caribbean of Costa Rica	-		-		-	
Cm-ToT-11_5	green (*Chelonia mydas*)	carapace soft tissue of hatchling	Tortuguero, Caribbean of Costa Rica	-		-		-	
Cm-ToT-11_6	green (*Chelonia mydas*)	carapace soft tissue of hatchling	Tortuguero, Caribbean of Costa Rica	-		-		-	
Cm-ToT-11_7	green (*Chelonia mydas*)	carapace soft tissue of hatchling	Tortuguero, Caribbean of Costa Rica	-		-		-	
Cm-ToT-11_8	green (*Chelonia mydas*)	carapace soft tissue of hatchling	Tortuguero, Caribbean of Costa Rica	-		-		-	
Cm-ToT-11_9	green (*Chelonia mydas*)	carapace soft tissue of hatchling	Tortuguero, Caribbean of Costa Rica	-		-		-	
Cm-ToT-12	green (*Chelonia mydas*)	tissue rear flipper	Tortuguero, Caribbean of Costa Rica	-		-		-	

				Viral detection by PCR assays					
**Sample Code**	**Species**	**Type of sample**	**Sample origen site (population)**	**Singleplex primer set UL18 (140bp)**	**Confirmed DNA sequence (F= forward, R=reverse, C=consensus of both F and R)**	**Singleplex primer set UL22 (179bp)**	**Confirmed DNA sequence (F= forward, R=reverse, C=consensus of both F and R)**	**Singleplex primer set UL27 (143bp)**	**Confirmed DNA sequence (F= forward, R=reverse, C=consensus of both F and R)**
Cm-ToT-13	green (*Chelonia mydas*)	tissue rear flipper	Tortuguero, Caribbean of Costa Rica	-		-		-	
Cm-ToT-14	green (*Chelonia mydas*)	tissue rear flipper	Tortuguero, Caribbean of Costa Rica	-		-		-	
Cm-ToT-15	green (*Chelonia mydas*)	tissue rear flipper	Tortuguero, Caribbean of Costa Rica	-		-		-	
Cm-ToT-16	green (*Chelonia mydas*)	tissue rear flipper	Tortuguero, Caribbean of Costa Rica	-		-		-	
Cm-ToT-17	green (*Chelonia mydas*)	tissue rear flipper	Tortuguero, Caribbean of Costa Rica	-		-		-	
Cm-ToT-18	green (*Chelonia mydas*)	tissue rear flipper	Tortuguero, Caribbean of Costa Rica	-		-		-	
Cm-ToT-19	green (*Chelonia mydas*)	tissue rear flipper	Tortuguero, Caribbean of Costa Rica	-		-		-	
Cm-ToT-20	green (*Chelonia mydas*)	tissue rear flipper	Tortuguero, Caribbean of Costa Rica	-		-		-	
Cm-ToT-21	green (*Chelonia mydas*)	tissue rear flipper	Tortuguero, Caribbean of Costa Rica	-		-		-	
Cm-ToT-22	green (*Chelonia mydas*)	tissue rear flipper	Tortuguero, Caribbean of Costa Rica	-		-		-	
Cm-ToT-23	green (*Chelonia mydas*)	tissue rear flipper	Tortuguero, Caribbean of Costa Rica	-		-		-	
Cm-ToT-25	green (*Chelonia mydas*)	tissue rear flipper	Tortuguero, Caribbean of Costa Rica	-		-		-	
Cm-ToT-26	green (*Chelonia mydas*)	tissue rear flipper	Tortuguero, Caribbean of Costa Rica	-		-		-	
Cm-ToT-27	green (*Chelonia mydas*)	tissue rear flipper	Tortuguero, Caribbean of Costa Rica	-		-		-	
Cm-ToT-28	green (*Chelonia mydas*)	tissue rear flipper	Tortuguero, Caribbean of Costa Rica	-		-		-	
Cm-ToT-29	green (*Chelonia mydas*)	tissue rear flipper	Tortuguero, Caribbean of Costa Rica	-		-		-	
Cm-ToT-30	green (*Chelonia mydas*)	tissue rear flipper	Tortuguero, Caribbean of Costa Rica	-		-		-	
Cm-ToT-31	green (*Chelonia mydas*)	tissue rear flipper	Tortuguero, Caribbean of Costa Rica	-		-		-	
Cm-ToT-32	green (*Chelonia mydas*)	tissue rear flipper	Tortuguero, Caribbean of Costa Rica	-		-		-	
Cm-ToT-33	green (*Chelonia mydas*)	tissue rear flipper	Tortuguero, Caribbean of Costa Rica	-		-		-	
Cm-ToT-34	green (*Chelonia mydas*)	tissue rear flipper	Tortuguero, Caribbean of Costa Rica	-		-		-	
Cm-ToT-35	green (*Chelonia mydas*)	tissue rear flipper	Tortuguero, Caribbean of Costa Rica	-		+	R	-	
Cm-ToT-36	green (*Chelonia mydas*)	tissue rear flipper	Tortuguero, Caribbean of Costa Rica	-		+	R	-	
Cm-ToT-37	green (*Chelonia mydas*)	tissue rear flipper	Tortuguero, Caribbean of Costa Rica	-		+	R	-	
Cm-ToT-38	green (*Chelonia mydas*)	tissue rear flipper	Tortuguero, Caribbean of Costa Rica	-		+	R	-	
Cm-ToT-39	green (*Chelonia mydas*)	tissue rear flipper	Tortuguero, Caribbean of Costa Rica	-		+	R	-	
Cm-ToT-40	green (*Chelonia mydas*)	tissue rear flipper	Tortuguero, Caribbean of Costa Rica	-		+	R	-	
Cm-ToT-41	green (*Chelonia mydas*)	tissue rear flipper	Tortuguero, Caribbean of Costa Rica	-		-		-	
Cm-TCFP-10	green (*Chelonia mydas*)	tumours FP	Turks & Caicos Islands, Caribbean	-		-		+	C
Cm-TCFP-11	green (*Chelonia mydas*)	tumours FP	Turks & Caicos Islands, Caribbean	-		+	R	-	
Cm-TCFP-12	green (*Chelonia mydas*)	tumours FP	Turks & Caicos Islands, Caribbean	+	R	+	R	+	R
Cm-TCFP-13	green (*Chelonia mydas*)	tumours FP	Turks & Caicos Islands, Caribbean	+	R	+	R	+	R
Cm-TCFP-14	green (*Chelonia mydas*)	tumours FP	Turks & Caicos Islands, Caribbean	+	R	+	R	+	C
Cm-TCFP-15	green (*Chelonia mydas*)	tumours FP	Turks & Caicos Islands, Caribbean	+	R	+	R	+	C
Cm-TCFP-1Carapace	green (*Chelonia mydas*)	tumours FP	Turks & Caicos Islands, Caribbean	-		-		+	F
Cm-TCFP-1cloaca1	green (*Chelonia mydas*)	tumours FP	Turks & Caicos Islands, Caribbean	-		+	R	-	
Cm-TCFP-1cloaca2	green (*Chelonia mydas*)	tumours FP	Turks & Caicos Islands, Caribbean	+	R	-		-	
Cm-TCFP-1cloaca4	green (*Chelonia mydas*)	tumours FP	Turks & Caicos Islands, Caribbean	+	R	-		-	
Cm-TCFP-1HL flipper	green (*Chelonia mydas*)	tumours FP	Turks & Caicos Islands, Caribbean	-		+	R	+	F
Cm-TCFP-1L flipper1	green (*Chelonia mydas*)	tumours FP	Turks & Caicos Islands, Caribbean	-		+	R	-	
Cm-TCFP-1L flipper2	green (*Chelonia mydas*)	tumours FP	Turks & Caicos Islands, Caribbean	-		+	R	-	
Cm-TCFP-1RF flipper	green (*Chelonia mydas*)	tumours FP	Turks & Caicos Islands, Caribbean	-		+	R	+	C
Cm-TCFP-1Rteye	green (*Chelonia mydas*)	tumours FP	Turks & Caicos Islands, Caribbean	-		+	R	-	
Cm-TCFP-2	green (*Chelonia mydas*)	tumours FP	Turks & Caicos Islands, Caribbean	-		+	R	+	C
Cm-TCFP-3	green (*Chelonia mydas*)	tumours FP	Turks & Caicos Islands, Caribbean	+	R	+	R	+	C
Cm-TCFP-4	green (*Chelonia mydas*)	tumours FP	Turks & Caicos Islands, Caribbean	+	R	+	R	+	R
Cm-TCFP-5	green (*Chelonia mydas*)	tumours FP	Turks & Caicos Islands, Caribbean	+	R	+	R	+	R
Cm-TCFP-6	green (*Chelonia mydas*)	tumours FP	Turks & Caicos Islands, Caribbean	-		+	R	-	
Cm-TCFP-7	green (*Chelonia mydas*)	tumours FP	Turks & Caicos Islands, Caribbean	-		-		+	C
Cm-TCFP-8	green (*Chelonia mydas*)	tumours FP	Turks & Caicos Islands, Caribbean	+	R	+	R	+	F
Cm-TCFP-9	green (*Chelonia mydas*)	tumours FP	Turks & Caicos Islands, Caribbean	+	R	+	R	-	
Cm-TCT-1RF flipper1	green (*Chelonia mydas*)	tissue- suspected tumours FP	Turks & Caicos Islands, Caribbean	-		-		-	
Cm-TCT-8leeches	green (*Chelonia mydas*)	tissue- suspected tumours FP	Turks & Caicos Islands, Caribbean	-		-		-	
