# Supplementary material for: Global distribution of Chelonid fibropapilloma-associated herpesvirus among clinically healthy sea turtles
Source: BMC Evol Biol. 2014 Oct 25;14:206. doi: 10.1186/s12862-014-0206-z (PMC4219010; doi:10.1186/s12862-014-0206-z)
Supplement: Additional file 8: — Primer sequences; and Description of data. Singleplex primer sequences designed to target highly conserved regions for three different genes in the herpesvirus genome [18]. [file 12862_2014_206_MOESM8_ESM.pdf]

Primer set	Targeted gene	Primer sequence (5’ to ’3)	Length of amplification (nucleotide bases)
UL18	Capsid protein gene UL18	**F:** GTGGAACCCCGCCGGGTAAT<br>**R:** TGATCCGGGCCGAGTAGCGG	140
UL22	Glycoprotein H gene UL22	**F:** AACGCCCTTTCCTCCGACCCATATT<br>**R:** GCTGGGGGAGCATCGTGCAAA	179
UL27	Glycoprotein B gene UL27	**F:** CTAGATACATACTGGCCRTGCTCGTC<br>**R:** GCCAGCGACCATCCGGAG	143
